# Synthesis and Investigations of Building Blocks with Dibenzo[*b*,*f*] Oxepine for Use in Photopharmacology

**DOI:** 10.3390/ijms222011033

**Published:** 2021-10-13

**Authors:** Piotr Tobiasz, Filip Borys, Marta Borecka, Hanna Krawczyk

**Affiliations:** 1 Department of Organic Chemistry, Faculty of Chemistry, Warsaw University of Technology, Noakowskiego 3, 00-664 Warsaw, Poland; ptobiasz@ch.pw.edu.pl (P.T.); fborys@gmail.com (F.B.); marta.borecka.stud@pw.edu.pl (M.B.); 2 Laboratory of Cytoskeleton and Cilia Biology, Nencki Institute of Experimental Biology of Polish Academy of Sciences, 3 Pasteur Street, 02-093 Warsaw, Poland

**Keywords:** *azo*-dibenzo[*b*,*f*]oxepine derivatives, photoisomerisation, tubulin inhibitor

## Abstract

The synthesis of photoswitchable *azo*-dibenzo[*b*,*f*]oxepine derivatives and microtubule inhibitors were described. Subsequently, we examined the reaction of methoxy derivative 3-nitrodibenzo[*b*,*f*]oxepine with different aldehydes and in the presence of BF_3_·OEt_2_ as a catalyst. Our study provided a very concise method for the construction of the *azo*-dibenzo[*b*,*f*]oxepine skeleton. The analysis of products was run using experimental and theoretical methods. Next, we evaluated the *E*/*Z* isomerization of *azo*-dibenzo[*b*,*f*]oxepine derivatives, which could be photochemically controlled using visible-wavelength light.

## 1. Introduction

Microtubules, dynamic intracellular polymers of tubulin, form part of the cytoskeleton and perform key functions in important processes such as mitosis, intracellular transport, and migration. Microtubule-directed small molecules, which inhibit the dynamics of a microtubule, are often used clinically as valuable tools in cell biology and chemotherapeutics, due to their ability to perturb mitosis [1,2,3]. Tumor cells have a greater capacity for cell proliferation and are also more susceptible to damage from microtubule inhibitors. However, due to the wealth of tubulin and the importance of microtubules, not only during mitosis but also in the interphase, the treatment with microtubule-directed drugs often leads to systemic side effects such as disease affecting the peripheral nerves (peripheral neuropathies) [4]. Therefore, the introduction of the spatially and temporally controlled activity of such drugs which could provide a significant advancement in tolerability, increasing their overall clinical value, is considered.

The use of light is one means to target therapy at the tumor site. The following, most well-known therapies are used: photodynamic therapy (PDT) [5,6,7] where the light-induced production of a singlet of oxygen is employed for tissue ablation, or optogenetics, where light is used to modulate the activity of genetically engineered ion channels, which are usually derived from photoresponsive rhodopsins [8,9]. These methods for targeting drug delivery are promising but, in some cases, the drug activation is irreversible upon illumination and, in the case of optogenetics, the clinical relevance is limited by the need for challenging genetic manipulation. One major problem for the clinical application of each of these light-activated drug systems is that the activated drug can diffuse away from the illuminated site of the tumor. This effect can cause unwanted damage to the tissues it encounters. It is important to search for the light-activated drugs which would automatically revert to less potent form over time to limit this off-target toxicity. A new area that is now intensively developing is photopharmacology [10,11,12]. Photopharmacological agents are bioactive molecules modified with photoswitches. Photoswitchable drugs are a group of compounds currently being intensively studied. Many research groups recently designed and synthesized molecular photoswitches based on the azobenzene scaffold [13,14], which could be operated in or near the therapeutic window of −650 nm–900 nm [15,16], (the light reaches deepest into the tissue, without being limited by the absorption by hemoglobin: λ < 650 nm and water λ > 900 nm).

Extrinsic factors such as microtubule-targeting agents (MTAs) can affect microtubules dynamics. The surface of the globular part of tubulins contains several pockets that can be intercalation sites for MTAs. The tubulin heterodimer contains at least six distinct drug binding sites: taxane, laulimalide/peloruside, vinca, maytansine, pironetin, and colchicine sites. Colchicine itself binds to tubulin very tightly, but its severe toxicity to normal tissues hampers its use in the clinic [17]. Combretastatins (CA-1 and CA-4, are a class of naturally occurring stilbene derivatives) [18,19,20] is a colchicine-domain microtubule inhibitor that binds to tubulins and thus inhibits their polymerization to form microtubules [21,22,23]. The scaffold of the CA-1 and CA-4 structure contain *Z*-stilbene, and it is known that the *E*-isomer is significantly about 60-fold less potent [24].

**Figure 1:**
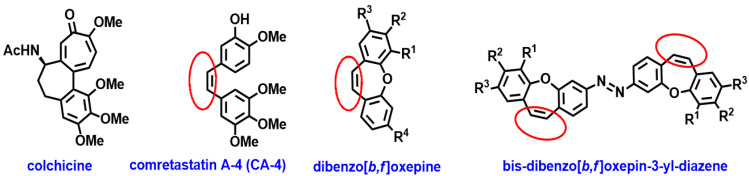
Structure of colchicine, combretastatin A-4, dibenzo[*b*,*f*]oxepine and 1,2-bis(dibenzo[*b*,*f*]oxepin-3-yl)diazene.

Continuing our study [25,26,27,28,29,30,31] of microtubule inhibitors, we analyzed the structure of active colchicine, active combretastatin A-4, and derivative dibenzo[*b*,*f*]oxepine (Figure 1). It can be observed that the substituents and backbone arrangements were similar. Additionally, the dibenzo[*b*,*f*]oxepine showed the strongest cytotoxic effect against HeLa and U87 cancerous cells [27] and had (*Z*)-stilbene motif in their skeleton. Additionally, their aromatic rings were connected by oxygen. Moreover, dibenzo[*b*,*f*]oxepine was an important scaffold in medicinal chemistry and its derivatives occurred in several medicinally important plants [32,33,34,35,36]. The molecules with this skeleton exhibited *anti*-depressive [37], antipsychotic [38,39], *anti*-estrogenic [40], antitumor [41] and *anti*-inflammatory [42] properties. Their activity as photoswitchable compounds was not investigated.

## 2. Results and Discussion

To study the activity of the photoswitchable compounds of dibenzo[*b*,*f*]oxepine we introduced an *azo* bond to the skeleton that could occur in configuration with *E* or *Z*. Before the synthesis of *azo*-dibenzo[*b*,*f*]oxepine derivatives, we performed a computational study (see Supporting Information) because 1,2-bis(dibenzo[*b*,*f*]oxepin-3-yl)diazene (Figure 1) also had a similar spatial structure to colchicine or combretastatins. 

**Scheme 1:**
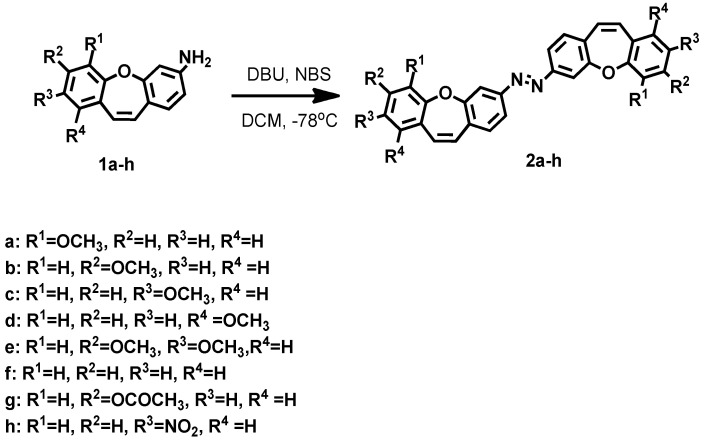
Synthesis of 1,2-bis(dibenzo[*b*,*f*]oxepin-3-yl)diazene (**2a–h**).

**Computational aspects**. We analyzed the geometry of the E/Z isomers in the *azo*-molecules (Scheme 1) using the density functional theory (DFT) calculations (**2f**). The optimum ground-state geometry for (**1a**, **1c**, **1e**, **1f**, **1h**, ***E***, **and *Z* 2f**) compounds was calculated using the density functional theory (DFT). In the calculations, the B3LYP functional and 6-311++g(2d,p) (***E*** and ***Z* 2f** basis set was employed and the continuum model (PCM; Gaussian 03W, see Supporting Information) was used to simulate the effects of the solvent, DMSO. The SCF energy for the *E*-**2f** isomer was 69.5 kJ/mol greater than for the *Z-***2f** isomer.

**Figure 2:**
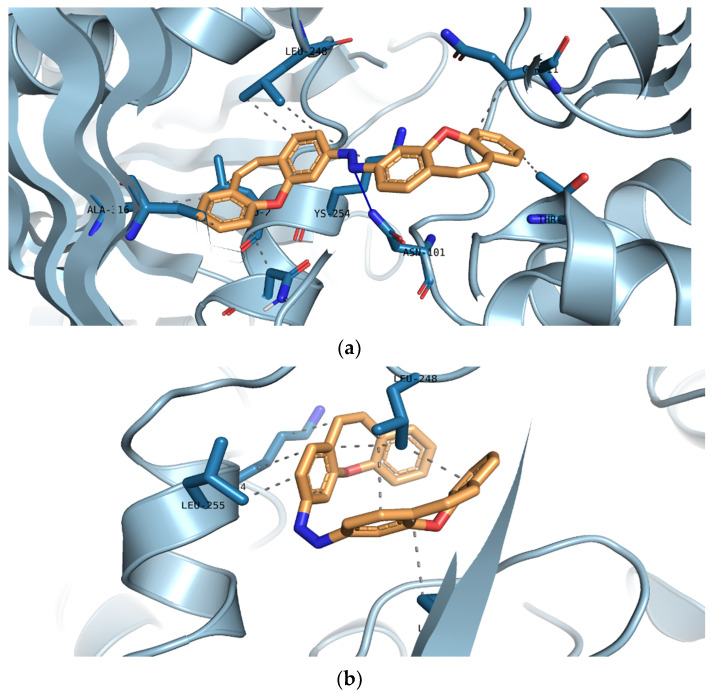
3D model of the interaction between the isomers, ***E*** (**a**) and ***Z*** (**b**), of (**2f**) and the colchicine binding site of α and β tubulin (crystal structure from PDB code: 1SA0).

**Molecular docking**. In the next step, we modeled the interaction between the isomers, *E*-**2f** and *Z*-**2f,** and the colchicine binding site of α and β-tubulin (Table 1 and Figure 2). The molecular docking of the compounds of the **2a–2c and 2e–2f** isomers, ***E*** and ***Z***, into the 3D X-ray structure of tubulin (PDB code: 1SA0) [43] was carried out using the Auto-Dock Vina software (the Broyden–Fletcher–Goldfarb–Shanno (BFGS) method) [44].

The configurations of the protein/dimethoxydibenzo[*b*,*f*]oxepine complex were created using UCSF Chimera software [45]. The graphical user interface, ADT, was employed to set up the enzyme and all the hydrogens were added. For macromolecules, the generated pdbqt files were saved. The 3D structures of ligand molecules were built, optimized (a B3LYP functional and 6–31* basis set level for the **2f *E***, ***Z* isomers**; for **2a–2c and 2e**, the methoxy groups were added to the calculated scaffold for the **2f *E***, ***Z* isomers**), and saved in Mol2 format. The graphical user interface, ADT, was also employed to set up the ligand and the pdbqt file was saved. The Auto-Dock Vina software was employed for all docking calculations. The AutoDockTools program was used to generate the docking input files. During docking, a grid box of size 34 × 34 × 34 points in the *x*, *y*, and *z* directions was built and the maps were center located (44.91, 54.25, −11.18) in the catalytic site of the protein. A grid spacing of 0.375 Å (approximately one–fourth of the length of a carbon–carbon covalent bond) was used for the calculation of the energetic map.

The structures of 1,2-bis(dibenzo[*b*,*f*]oxepin-3-yl)diazene **2a–2c**, **and the 2e** isomers, ***E*** and ***Z***, had an estimated binding free energy presented in Table 1 (the binding free energy of the control compounds, colchicine and CA-4, are −8.6 kcal/mol and −7.62 kcal/mol, respectively [46,47]). The model was similar to the models between colchicine, CA-4, and the colchicine binding site [48,49]. In the isomers, ***E*** and ***Z***, of the **2f** binding models; more details revealed that they played some key roles in the interaction between compounds and tubulin (Figure 2). The compound **2f** was embedded in the hydrophobic pocket occupied by the A ring of the colchicine binding site (hydrophobic interactions with Leu248, Lys254, Leu255, Lys352 for the ***Z*** isomer and hydrophobic interactions with Gly11, Thr145, Leu248, Lys254, Leu255, Asn258, Ala316, Lys352, and the hydrogen bond with Asn101 for the ***E*** isomer) of α and β tubulin (crystal structure from PDB code: 1SA0). 

Based on the data from the calculations, we would expect that *azo*-dibenzo[*b*,*f*]oxepine derivatives would be a potent tubulin inhibitor; therefore, we synthesized and investigated a set of the *azo* compounds (Table 2 and Scheme 1). These compounds may find applications in photopharmacology. [12,50] Firstly, we explored the various synthetic methods to obtain the *azo* compounds (**2a–2h**). We focused on one-step methods because of the short reaction time, although different varieties of azo-product reduction (two-step from the amino group) are also currently under investigation [51,52,53,54,55]. The results are summarized in Table 2. The methods with the *tert*-Butyl nitrite [56]; NBS, NCS, NIS with DBU, DBN; KO*^t^*Bu in −78 °C [57]; or various variants of KMnO_4_ [57] with oxone synthesis [58], or KOH in DMF at 150 °C [59], were examined, respectively. The compound (**2a**) was obtained in eight cases. The limited yields in the preparation of *azo*-dibenzo[*b*,*f*]oxepines may result from the spatial structure of the molecules. We made additional calculations which showed that the dibenzo[*b*,*f*]oxepin-3-amine scaffold was not planar and that it adopted a basket conformation. The dihedral angles between the aromatic rings connected with oxygen and a double bond (for example, the two dihedral angles: C=C-O-C (one ring) and C=C-O-C (second ring), for dibenzo[*b*,*f*]oxepine **1f** were 64.9°/65.9° (the geometry and dihedral angles for **1a**, **1c**, **1e**, **1f** and **1h** are found in the Supporting Information). Although the geometry shows that the amino group is in a plane with the aromatic ring, the lack of planarity of the further part of the molecule may weaken the diazotization reaction during the process of creating an N–N bond [57]. Based on these results, we concluded that the first method was optimal for obtaining the *azo*-product. We synthesized a series of the *azo* compounds(**2a–2h**) in one synthetic step (Scheme 1).

Complete ^1^H, ^13^C NMR, and UV spectroscopic data for all the products (**2a–2h**) are shown in the Experimental section (see Supporting Information). The ^1^H and ^13^C NMR resonances were assigned unequivocally based on the combined information from 1D to 2D NMR (gs-COSY, gs-HSQC, and gs-HMBC) experiments. Coupling constants (^1^H–^1^H) were measured directly from resolution-enhanced 1D spectra and confirmed, when necessary, by homo-decoupling. 

**Scheme 2:**
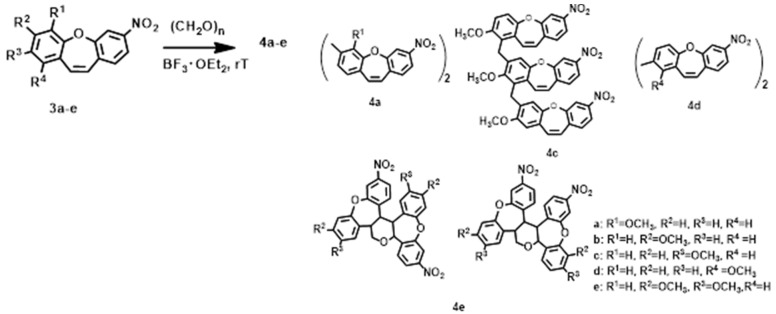
Synthesis of compounds (**4a–4e**).

In search of new photo-switching connections, we next examined the reaction of methoxy derivative 3-nitrodibenzo[*b*,*f*]oxepines with paraformaldehyde and in the presence of a Lewis acid as the catalyst. The following Lewis acids were explored: ZnCl_2_, AlCl_3_, TiCl_4_, scandium triflate, and BF_3_·OEt_2_ in room temperature, DCM, and under argon. The best yield was for the BF_3_·OEt_2_ and so we chose it for further studies (Table S1, see Supporting Information). The results are summarized in Scheme 2. In the case of (**3a**), (**3d**) the products are dimmers. In the reaction with (**3b**) instead of a product (**4b**), a mixture of difficult to separate compounds was formed. Five products were possible to obtain in this reaction with (**3c**) (Figure S1, see Supporting Information). Based on the two-dimensional NMR spectra we concluded that this was the structure of the product (**4c4**, see Supporting Information): a compound formed from the substitution of two dibenzo[*b*,*f*]oxepines with the substrate (**3c**). The Products of Prins reaction was observed for the substrate (**3e**) and the mixing of two products (**4e**) was visible in the NMR spectra (see the spectra in the Supporting Information).

As well as the differences in the course of the reaction, methoxy 3-nitrodibenzo[*b*,*f*]oxepines with paraformaldehyde were also related to the differences in electron densities (**3a–3d**) and steric hindrance (**3e**). To obtain a clearer picture of the changes induced by a substituent, we used the SCS [60] parameter (substituent-induced chemical shifts, Table 3) for the ^13^C NMR chemical shifts. The interpretation of the SCS in the ^13^C NMR spectra was a well-established method for investigating the electronic interactions in various molecular systems despite the absence of any simple and general relationship between the chemical shifts and the electron density at a given nucleus [61,62,63]. The data in Table 3 were collected in the form of substituent-induced chemical shifts. For the carbon-labeled i, SCS was defined as the difference between the chemical shift in the parent compound HX: SCSi (RX) = δi(RX) − δi(HX). By analysing these data, we observed that the substituent effect on the ^13^C chemical shift was greatest in its position *ortho* relative to methoxy group: −17.8 for (**3a**), −14.34 and −13.93 (Δ = 0.41) for (**3b**), −14.87 and −15.84 (Δ = 0.97) for (**3c**), and −17.37 for (**3d**) which justified the reactivity (increased of electron density) in this position **to** electrophile attack. On the other hand, for the substrate (**3e**), the Prins reaction and the mixing of products (**4e**) were most likely due to the steric hindrance in the aromatic ring.

To expand the scope of this reaction, we examined the reaction of **3a** in the presence of various aldehydes. The results are summarized in Table 4. The best yield and the shortest reaction time were observed for 2,4-dinitrobenzaldehyde. The remaining aldehydes reacted slowly at room temperature and the product obtained was less in yield. In the case of 2,6-dinitrobenzaldehyde, it was necessary to increase the temperature to 45 °C. In all cases (**5a–5e**) without (**5f**), one product was created: dimmer dibenzo[*b*,*f*]oxepine with an aromatic linker. In the case of (**3f**) aldehyde, created due to the high hindrance in the molecule, the product electrophilic substitution of the olefin bond, one dibenzo[*b*,*f*]oxepine, with the aromatic ring second molecule of dibenzo[*b*,*f*]oxepine was observed (**5f**). To analyze **the structures** of the molecules from the compounds (**5a–5e**), the optimum structure was calculated using the DFT B3LYP/6-31g*) method (see Supporting Information).

**Scheme 3:**
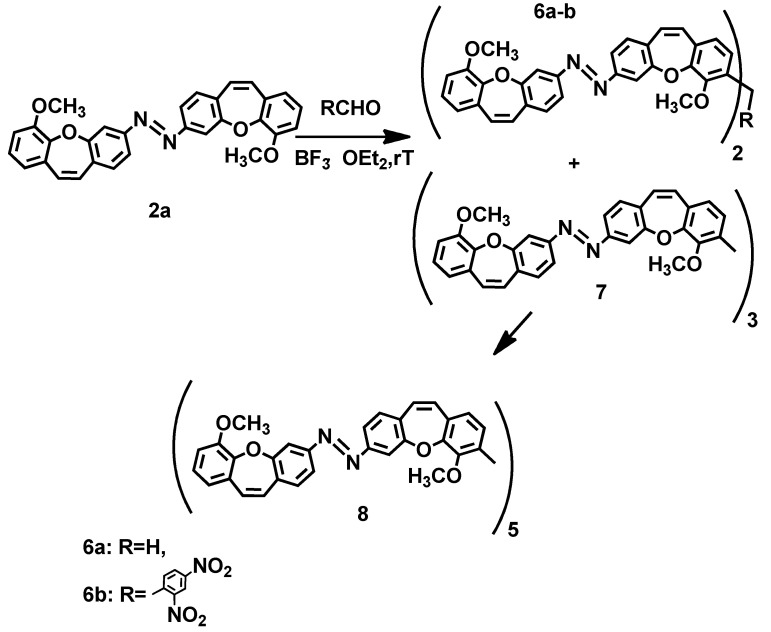
Synthesis of compounds (**6a–6b**) and (**8**).

In the next step, we applied these reaction conditions to create new *azo*-compounds. We examined the reaction of aldehydes with the compound (**2a**) (Scheme 3). Only in the case of using paraformaldehyde and 2,4-dinitrobenzaldehyde were the products obtained (**6a**, **6b**). Moreover, in the case of the compound (**6a**), the compound (**7**, 5% yield) was also observed, which, with the compound (**6a**, 49% yield), most likely in a subsequent reaction, formed a pentamer (**8**, 40% yield). In the reaction with the next benzaldehyde, only one product (**6b**, 17% yield) could be obtained.

**Figure 3:**
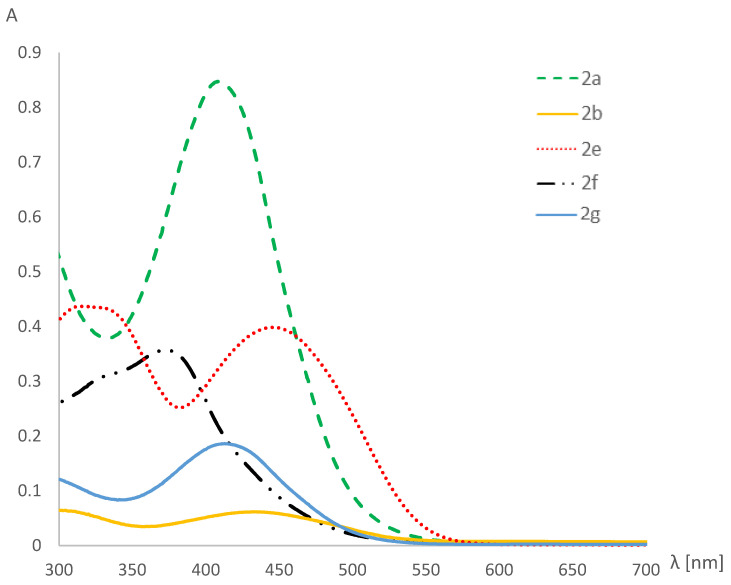
UV/VIS spectra of compounds (**2a**, **2b**, **2e–2g**).

**Photoisomerisation in DMSO, studied by UV-VIS and NMR**. The light-induced isomerization of the *azo*-dibenzo[*b*,*f*]oxepines photoswitch from the trans (*E*) form to the thermodynamically less stable cis (*Z*) form was associated with significant changes in geometry and polarity. Therefore, for the obtained *azo*-compounds (**2a**, **2b**, **2e–2g**), we measured the UV/VIS spectra (Figure 3 and Table S2, see Supporting Information). The examination of the UV/VIS spectra of the *trans* -(**2a**, **2b**, **2e–2g**) spectra allowed for the determination of the maximum for the transitions π−π*: 409 nm; 434 nm; 446 nm; 371 nm; 400 nm, respectively.

**Figure 4:**
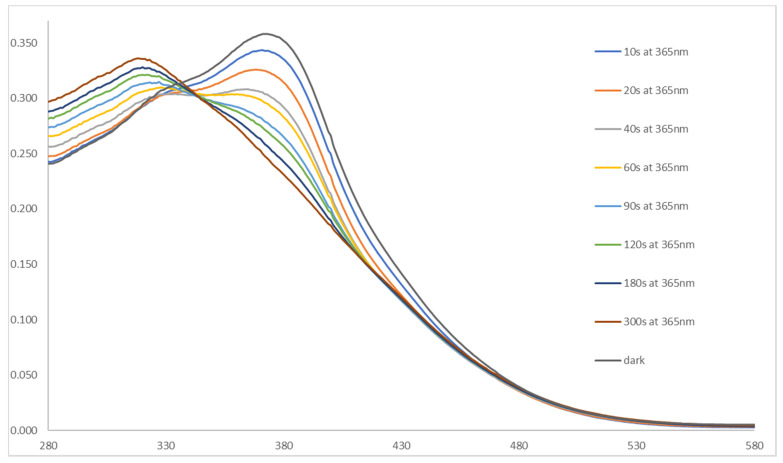
The hypsochromic shift in UV/VIS spectra of compound (**2f**) upon irradiation with 365 nm.

**Figure 5:**
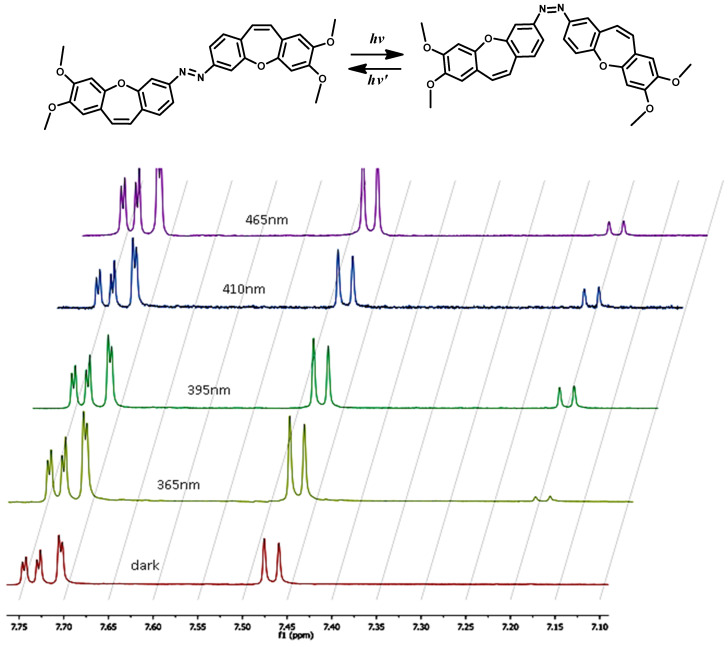
Photoisomerization of compound (**2e**) measured by ^1^H NMR. Aromatic ^1^H NMR spectra in the dark-adapted state and at various PSSs measured under constant illumination at the indicated wavelengths.

Next, we analyzed the photoswitching properties of **2f** (Figure 4). The photoisomerization was measured for 10 µM **2f** solutions in DMSO upon 365 nm irradiation. After the illumination, other spectra were obtained (Figure 4) and the compound’s absorption pattern dramatically shifted toward an absorption profile that was characteristic of the form, *Z.* In general, it was very difficult, if not impossible, to isolate the *Z* isomer of (**2a**, **2b**, **2e–2g**) which thermally relaxed on a timescale faster than several hours. Thus it was difficult to predict the optimal wavelengths without a spectrum of the pure *Z* isomer. The UV/VIS spectra of (**2a**, **2b**, **2e–2g**) generated at various wavelengths were sufficient to determine the optimal wavelengths for the photoconversion between the isomers’ conversions. However, this information did not reveal the ratio of photoisomers. Because the absorbance spectra of both isomers overlapped substantially, it was very difficult to predict the *E/Z* isomeric content using the UV/VIS spectra, particularly at intermediate wavelengths. The use of ^1^H NMR spectroscopy simplified matters considerably (Table 5, Figure 5). Thus, the maximal extent of the photoconversion between the isomers that could be achieved was readily determined. By providing a direct measurement of the photostationary states consisting of different ratios of photoisomers, the estimations, as used in UV/VIS experiments, were not required.

One serious restriction in the development of functional photoswitching systems is the necessity to trigger switching in at least one direction by UV light, which is often damaging, e.g., short-and longwave ultraviolet light damages DNA. For humans, sunburn is the familiar effect of exposure of the skin to UV light, along with an increased risk of skin cancer [64]. Scientists are searching for compounds that can be switched in visible light. During the exposure to different wavelengths (from dark to 465 nm, Figure 5) of (2a, 2b, 2e, 2g) the isomers’ new signals can be observed. There is a photoisomerization of the isomer *E* to the desirable isomer *Z* (skeleton (*Z*)- stilbene motif). *Z* isomers of investigated compounds have a lifetime of approximately 1 day. We can conclude that the obtained compounds convert from the *E* to the *Z* isomers over a safe wavelength range (410 nm and 465 nm) and can be potentially photochemically controlled using visible wavelength light.

## 3. The Application in Photopharmacology

Considering medical applications, we anticipate that the *Z* isomers of azo- dibenzo[*b*,*f*]oxepine derivatives will share many of the features that especially suit the colchicine binding site of α and β tubulin for tumor chemotherapy (Figure 2). It is known for many molecules that the scaffold structure containing the Z-compound is about 60-fold more potent than the *E* isomer[24]. Crucially, the *azo*- dibenzo[*b*,*f*]oxepine isomers may avoid the therapeutically limiting side effects of the current MT inhibitors, as the compounds applied globally may be activated locally by the precise irradiation in vivo. This can be used in light delivery methods, such as in photodynamic therapy [65], and also in optogenetics [12,66,67]. It may be possible to use dual-wavelength irradiation to actively restrain the Z form inside the organism at the destination. We also anticipate that the azo- dibenzo[*b*,*f*]oxepine derivatives’ spontaneous *Z*/*E* relaxation, on the scale of 1 day, can passively reduce the systemic exposure to bioactive *Z*-isomers. Obtained by us, these compounds may be used to deliver stronger on-target effects than those achieved with the current, globally active MT inhibitors, while simultaneously reducing the accompanying side effects. Therefore, we anticipate that *azo*- dibenzo[*b*,*f*]oxepine derivatives will be useful tools for cell biology and cancer chemotherapy, using the spatiotemporal precision of photopharmacology.

## 4. Conclusions

In summary, we synthesized *azo*-dibenzo[*b*,*f*]oxepine derivatives, using the NBS method and with DBU in dichloromethane. Next, we examined the dibenzo[*b*,*f*]oxepine scaffold in the reaction with aldehydes and BF_3_·OEt_2_. Later, we applied these reaction conditions to create new *azo*-compounds. The reactions proceeded under very simple reaction conditions. Our study provided a very concise method for the construction of the *azo*-dibenzo[*b*,*f*]oxepine skeleton. Furthermore, we explored the switchable properties of obtained compounds. To our knowledge, this is the first time a system utilizing dibenzo[*b*,*f*]oxepine with an azo moiety could be photocontrolled using only visible light (410 nm, 465 nm). The further investigation of the reaction to the synthesis of various *azo*-dibnzo[*b*,*f*]oxepine, could mean that this compound is considered a potential molecular substitute for use in photopharmacology, and studies are ongoing.

## Figures and Tables

**Table 1:** Estimated binding free energy (**kJ/mol**) of **2a–2c and 2e–2f**, isomers ***E*** and ***Z***.

Compound	The Affinity of Isomer *E* (kJ/mol)	The Affinity of Isomer *Z* (kJ/mol)	Δ Affinity (kJ/mol)
**2a**	–13.9	–12.3	1.6
**2b**	–13.5	–14.2	–0.7
**2c**	–14.2	–13.5	0.7
**2e**	–12.8	–12.4	0.4
**2f**	–13.4	–12.4	1

**Table 2:** Screening of reaction methods.

Method	Conditions	Yield
**1** [57]	NBS, DBU, CH_2_Cl_2_, Ar, −78 °C	45%
**2** [56]	*t*-BuONO (1.2 eq.), EtOH, rt, 48 h	0%
**3** [57]	KMnO_4_, CuSO_4_·5H_2_O, CH_2_Cl_2_,24 h, rt	20%
**4** [57]	KMnO_4_, CuSO_4_·5H_2_O, CH_2_Cl_2_, grinding	10%
**5** [58]	(1) oxone, H_2_O, CH_2_Cl_2_(2) AcOH, TFA, toluene	5%
**6** [57]	NCS, DBU, CH_2_Cl_2_, Ar, −78 °C	45%
**7** [57]	NIS, DBU, CH_2_Cl_2_, Ar, −78 °C	37%
**8** [57]	NCS, DBN, CH_2_Cl_2_, Ar, −78 °C	10%
**9** [57]	NCS, KO*^t^*Bu, CH_2_Cl_2_, Ar, −78 °C	20%
**10** [59]	KOH, DMF, N_2_, 150 °C	mixture

**Table 3:**
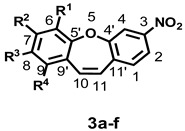
Carbon-13 chemical shifts for 3-nitrodibenzo[*b*,*f*]oxepine, substituent-induced chemical shifts for its methoxy substituted derivatives.

R	C1	C2	C3	C4	C4′	C5′	C6	C7	C8	C9	C9′	C10	C11	C11′
R^1^–R^4^ = H, *	**131.12**	**121.26**	**149.05**	**117.46**	**156.93**	**156.86**	**122.35**	**132.06**	**126.73**	**130.80**	**130.43**	**134.36**	**129.10**	**138.01**
**3(a); R_1_ = OCH_3_,** **R^2^–R^4^ = H**	**–0.05**	**0.02**	**−0.19**	**−0.27**	**−0.2**	**−12.4**	**29.91**	**−17.08**	**−4.98**	**−3.99**	**1.11**	**0.03**	**0.09**	**0.3**
**3(b); R_2_ = OCH_3_,** **R^1^, R^3^,R^4^ = H**	**–0.27**	**0.07**	**−0.29**	**0.23**	**−0.66**	**1.09**	**–14.34**	**30.97**	**−13.93**	**0.82**	**−7.34**	**−0.08**	**−2.49**	**0.54**
**3(c); R_3_ = OCH_3_,** **R^1^, R^2^,R^4^ = H**	**0.08**	**−0.13**	**0.01**	**–0.18**	**0.35**	**−6.34**	**0.75**	**−14.87**	**30.84**	**−15.84**	**0.69**	**−0.06**	**0.32**	**−0.03**
**3(d); R_4_ = OCH_3_,** **R^1^–R^3^ = H**	**−0.25**	**0.12**	**−0.05**	**0.03**	**0.04**	**1.72**	**−7.89**	**0.51**	**−17.37**	**27.18**	**−11.26**	**−5.41**	**−0.73**	**0.58**
**3(e); R_2_,R_3_ = OCH_3_,** **R^1^, R^4^ = H**	**−0.37**	**−0.13**	**−0.25**	**0.04**	**−0.33**	**−6.38**	**−15.64**	**19.98**	**20.33**	**−18.02**	**−8.43**	**0.12**	**−1.99**	**0.55**
3(f) *; R_1_, R_3_ = OCH_3_, **R^2^, R^4^ = H**	**−0.01**	**−0.14**	**−0.21**	**−0.49**	**0.13**	**−18.31**	**30.42**	**−29.70**	**31.00**	**−25.95**	**1.12**	**0.15**	**0.36**	**0.26**

* Data from: H. Krawczyk, M. Wrzesiński, D. Mielecki, P. Szczeciński E. Grzesiuk, *Tetrahedron* **2016**, *72*, 3877–3884.

**Table 4:**
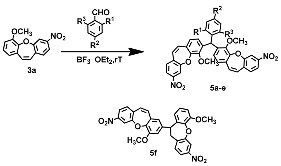
Synthesis of (**5a–5f**) from **3a**.

Compound 3 or 5	R	Yield (%) of 5
**a**	R^1^=NO_2_, R^2^=H, R^3^=H	26%
**b**	R^1^=H, R^2^=NO_2_, R^3^=H	36%
**c**	R^1^=NO_2_, R^2^=NO_2_, R^3^=H	66%
**d**	R^1^=Cl, R^2^=H, R^3^=H	9%
**e**	R^1^=Br, R^2^=Cl, R^3^=H	14%
**f**	R^1^=NO_2_, R^2^=H, R^3^=NO_2_	43%

**Table 5:** Percentages of (***Z***) (**2a**, **2b**, **2e**, **2g**) isomers obtained from NMR spectra and at various PSSs (photostationary state) measured under constant illumination at the indicated wavelengths.

Compound		Percent of (*Z*) Isomer at PSS at			
		365 [nm]	395 [nm]	410 [nm]	465 [nm]
**2a**	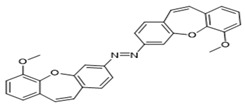	44	51	46	48
**2b**	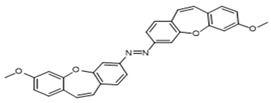	28	47	50	48
**2e**	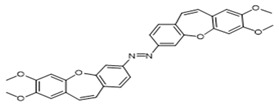	15	40	45	42
**2g**	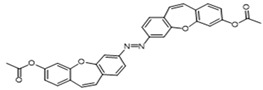	38	49	51	49

## Data Availability

Supporting Information.

## Supplementary Materials

The following are available online at https://www.mdpi.com/article/10.3390/ijms222011033/s1.
