# Supplementary material for: Synthesis and Investigations of Building Blocks with Dibenzo[b,f] Oxepine for Use in Photopharmacology
Source: Int J Mol Sci. 2021 Oct 13;22(20):11033. doi: 10.3390/ijms222011033 (PMC8539288; doi:10.3390/ijms222011033)
Supplement: Supplementary file 1 [file ijms-22-11033-s001.zip › ijms-1373508-supplementary.pdf]

Synthesis and investigations of building blocks with dibenzo[*b,f*] oxepine for use in photopharmacology

Piotr Tobiasz,[1] Filip Borys, [1,2] Marta Borecka,[1] and Hanna Krawczyk,*[1]

1 Department of Organic Chemistry, Faculty of Chemistry, Warsaw University of Technology, Noakowskiego 3,00-664 Warsaw, Poland. hkraw@ch.pw.edu.pl

2 Laboratory of Cytoskeleton and Cilia Biology Nencki Institute of Experimental Biology of Polish Academy of Sciences, 3 Pasteur Street, 02-093Warsaw, Poland.

* Correspondence: hkraw@ch.pw.edu.pl (H.K.)

Supporting Information

Table of Contents:

1. **Computational aspects** 2
2. **Experimental section** 27
3. **NMR spectra of obtained compounds** 35
4. **Table 2S** 95

References 96

1. Computational aspects

The optimum ground-state geometry for (**1a**,**1c**, **1e**, **1f**, **1h**, ***E* and *Z* 2f, 3e, and 5a-5e**) compounds was calculated using the density functional theory (DFT). In calculation, the B3LYP functional and 6-311++g(2d,p) for (***E* and *Z* 2f**) and /6-31G* for (**3e and 5a-5e**) basis set was employed and the continuum model (PCM; Gaussian 03W) [1,2] was used to simulate the effects of the solvent. All the calculations were performed on a server equipped with a 16 quad-core XEON (R) CPU E7310 processor operating at 1.60 GHz. The operating system was Open SUSE 10.3. in DMSO as a solvent.

- The calculated coordinates of (**1a**) (the part of calculated log file) -

Standard orientation:

Center Number	Atomic Number	Atomic Type	Coordinates (Angstroms) X	Y	Z
1	6	0	-3.278899	0.697497	-0.161357
2	6	0	-3.637068	-0.612732	-0.535229
3	6	0	-2.728121	-1.651760	-0.397850
4	6	0	-1.423456	-1.457357	0.096675
5	6	0	-1.090580	-0.140855	0.472591
6	6	0	-1.983918	0.913838	0.347841
7	6	0	-0.504551	-2.583824	0.208374
8	6	0	0.846777	-2.558040	0.220614
9	6	0	1.710606	-1.386086	0.073210
10	6	0	1.295170	-0.085050	0.419184
11	8	0	0.113496	0.137188	1.111961
12	6	0	3.001235	-1.543697	-0.465965
13	6	0	3.841162	-0.452452	-0.671149
14	6	0	3.407807	0.831563	-0.341806
15	6	0	2.134192	1.018118	0.203252
16	7	0	-4.198006	1.734211	-0.219841
17	8	0	1.724860	2.274486	0.594443
18	6	0	1.226382	3.086179	-0.472046
19	1	0	-4.634177	-0.806803	-0.922532
20	1	0	-3.026312	-2.656295	-0.689854
21	1	0	-1.676996	1.899387	0.685693
22	1	0	-0.980559	-3.563597	0.218739
23	1	0	1.366740	-3.513966	0.240304

24	1	0	3.336827	-2.544217	-0.727133
25	1	0	4.835546	-0.600917	-1.082775
26	1	0	4.051104	1.696801	-0.475110
27	1	0	-3.812029	2.670922	-0.263055
28	1	0	-4.966887	1.608358	-0.868966
29	1	0	0.969827	4.052353	-0.031135
30	1	0	1.985080	3.232953	-1.251755
31	1	0	0.330603	2.640097	-0.922792

After PCM corrections, the SCF energy is -784.596325046 a.u.

Visualization of calculated geometry of (**1a**) :

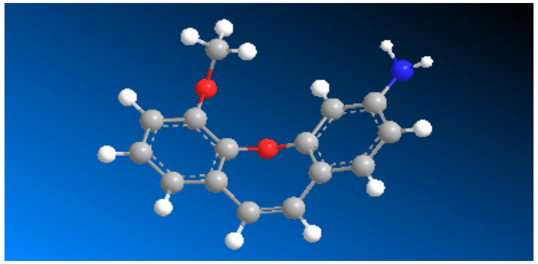

- The calculated coordinates of (**1c**) (the part of calculated log file) -

Standard orientation:

Center Number	Atomic Number	Atomic Type	Coordinates (Angstroms) X	Y	Z
1	6	0	-3.896988	-0.633507	0.425285
2	6	0	-4.027770	0.735629	0.730084
3	6	0	-2.999106	1.618159	0.433640
4	6	0	-1.792939	1.202988	-0.163857
5	6	0	-1.691783	-0.167850	-0.477260
6	6	0	-2.707611	-1.068413	-0.189547
7	6	0	-0.736828	2.171981	-0.432578
8	6	0	0.589971	1.944110	-0.556077
9	6	0	1.278847	0.661369	-0.411595
10	6	0	0.653704	-0.574332	-0.646080

11	8	0	-0.617639	-0.654655	-1.213985
12	6	0	2.625944	0.651922	0.007193
13	6	0	3.315589	-0.546290	0.202466
14	6	0	2.662341	-1.767525	-0.021883
15	6	0	1.337454	-1.772490	-0.442929
16	7	0	-4.891966	-1.539973	0.762197
17	8	0	4.618717	-0.635348	0.606939
18	6	0	5.334155	0.571166	0.840575
19	1	0	-4.941352	1.097514	1.195165
20	1	0	-3.121784	2.672093	0.673530
21	1	0	-2.578056	-2.109562	-0.471661
22	1	0	-1.065440	3.210029	-0.467274
23	1	0	1.238948	2.808737	-0.682393
24	1	0	3.110909	1.605838	0.183767
25	1	0	3.209029	-2.693690	0.126313
26	1	0	0.822748	-2.706966	-0.645420
27	1	0	-4.896950	-2.408145	0.237858
28	1	0	-5.824095	-1.154211	0.866889
29	1	0	6.337262	0.269716	1.147722
30	1	0	5.401402	1.182505	-0.068662
31	1	0	4.872629	1.164474	1.640533

After PCM corrections, the SCF energy is -784.604019661 a.u.

Visualization of calculated geometry of (**1c**) :

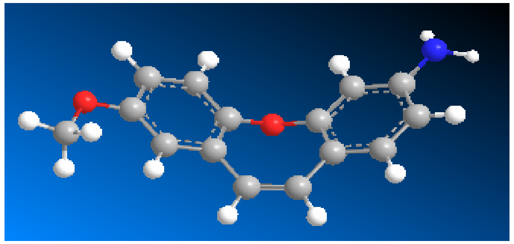

- The calculated coordinates of (**1e**) (the part of calculated log file) -

Standard orientation:

Center Number	Atomic Number	Atomic Type	Coordinates (Angstroms) X	Y	Z

1	6	0	4.249356	-0.939630	-0.439081
2	6	0	4.603264	0.404977	-0.664291
3	6	0	3.723492	1.424257	-0.329321
4	6	0	2.455105	1.177603	0.231621
5	6	0	2.130696	-0.174038	0.466017
6	6	0	2.993751	-1.209836	0.136826
7	6	0	1.562232	2.289000	0.539682
8	6	0	0.213664	2.269837	0.632291
9	6	0	-0.666041	1.121684	0.418344
10	6	0	-0.252358	-0.207771	0.593261
11	8	0	0.978845	-0.523655	1.164117
12	6	0	-1.991589	1.340402	-0.013566
13	6	0	-2.867384	0.289470	-0.281378
14	6	0	-2.420578	-1.039431	-0.099537
15	6	0	-1.117466	-1.268745	0.332955
16	7	0	5.093270	-1.975377	-0.816026
17	8	0	-4.150248	0.437438	-0.727827
18	6	0	-4.651742	1.758016	-0.899346
19	8	0	-3.222968	-2.117555	-0.386870
20	6	0	-4.333882	-2.315616	0.497807
21	1	0	5.572059	0.640005	-1.098145
22	1	0	4.017727	2.456055	-0.508784
23	1	0	2.693273	-2.230192	0.358226
24	1	0	2.051339	3.257786	0.633763
25	1	0	-0.292497	3.219717	0.794972
26	1	0	-2.315531	2.365815	-0.154408
27	1	0	-0.783379	-2.288958	0.490818
28	1	0	4.949710	-2.858333	-0.337961
29	1	0	6.077047	-1.738456	-0.885165
30	1	0	-5.679944	1.642894	-1.246447
31	1	0	-4.645780	2.316183	0.045462
32	1	0	-4.073831	2.311384	-1.650445
33	1	0	-4.831954	-3.226386	0.157383
34	1	0	-3.986692	-2.455483	1.529602
35	1	0	-5.032590	-1.475286	0.454240

After PCM corrections, the SCF energy is -899.119880495 a.u.

Visualization of calculated geometry of (**1e**) :

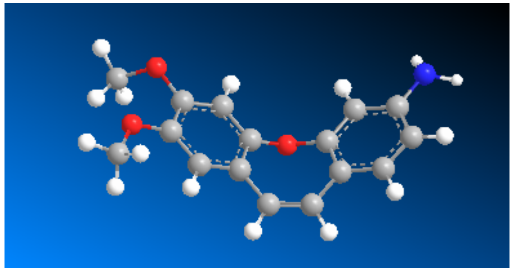

- The calculated coordinates of (**1f**) (the part of calculated log file) -

Standard orientation:

Center Number	Atomic Number	Atomic Type	Coordinates (Angstroms) X	Y	Z
1	6	0	3.180548	-0.696558	-0.212032
2	6	0	3.376480	0.663961	-0.519840
3	6	0	2.345744	1.575566	-0.341794
4	6	0	1.075025	1.199709	0.135883
5	6	0	0.908741	-0.162678	0.456729
6	6	0	1.924426	-1.092358	0.285158
7	6	0	0.020321	2.196773	0.276453
8	6	0	-1.316608	1.997844	0.267362
9	6	0	-2.019289	0.728394	0.082663
10	6	0	-1.454106	-0.521896	0.403111
11	8	0	-0.245701	-0.616498	1.087932
12	6	0	-3.320338	0.724425	-0.456028
13	6	0	-4.016239	-0.459855	-0.687599
14	6	0	-3.422003	-1.685455	-0.379097
15	6	0	-2.137759	-1.712111	0.167783
16	7	0	4.180522	-1.632552	-0.434107
17	1	0	4.341302	0.996505	-0.894575
18	1	0	2.518551	2.622006	-0.583488
19	1	0	1.740699	-2.125127	0.567818
20	1	0	0.367323	3.228271	0.323127
21	1	0	-1.955535	2.878137	0.308354
22	1	0	-3.781954	1.678761	-0.699205
23	1	0	-5.019056	-0.425983	-1.104310
24	1	0	-3.956601	-2.615967	-0.548912
25	1	0	-1.660207	-2.647704	0.443208
26	1	0	4.109681	-2.492216	0.099327
27	1	0	5.128096	-1.271410	-0.448565

After PCM corrections, the SCF energy is -670.081543145 a.u.

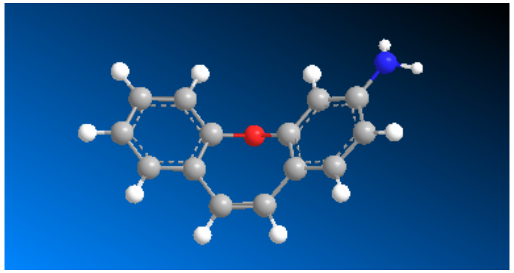

Visualization of calculated geometry of (**1f**) :

- The calculated coordinates of (**1h**) (the part of calculated log file) -

Standard orientation:

Center Number	Atomic Number	Atomic Type	Coordinates (Angstroms) X	Y	Z
1	6	0	4.116657	-0.785623	-0.402910
2	6	0	4.333612	0.568050	-0.729872
3	6	0	3.355619	1.514930	-0.467522
4	6	0	2.116414	1.184696	0.117040
5	6	0	1.932039	-0.170584	0.451201
6	6	0	2.892571	-1.136849	0.200679
7	6	0	1.117147	2.219633	0.338921
8	6	0	-0.220691	2.077942	0.467415
9	6	0	-0.988120	0.836800	0.381761
10	6	0	-0.446344	-0.440611	0.655321
11	8	0	0.818123	-0.597940	1.179141
12	6	0	-2.340365	0.891926	0.014717
13	6	0	-3.094922	-0.273602	-0.093406
14	6	0	-2.549399	-1.532341	0.160636
15	6	0	-1.213140	-1.602108	0.536109
16	7	0	5.057471	-1.753505	-0.702186
17	7	0	-4.501639	-0.173034	-0.477383
18	8	0	-5.155787	-1.215918	-0.565406
19	8	0	-4.968755	0.948247	-0.695527
20	1	0	5.274259	0.866217	-1.185634
21	1	0	3.543534	2.554492	-0.726024
22	1	0	2.697064	-2.161853	0.502197
23	1	0	1.503898	3.237619	0.332003
24	1	0	-0.818131	2.982589	0.555740
25	1	0	-2.808671	1.848069	-0.187674
26	1	0	-3.161147	-2.421212	0.073021
27	1	0	-0.747792	-2.554925	0.766373
28	1	0	4.998710	-2.620307	-0.179516

```
   29          1             0        6.012323   -1.433575   -0.819695
 ---------------------------------------------------------------------
After PCM corrections, the SCF energy is -874.587173635     a.u.
```

Visualization of calculated geometry of (**1h**) :

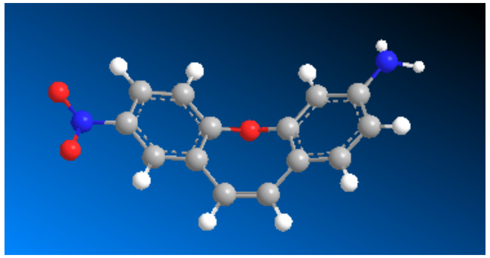

- The plane and dihedral angles in amines **1a**,**1c**, **1e**, **1f**, **1h**.

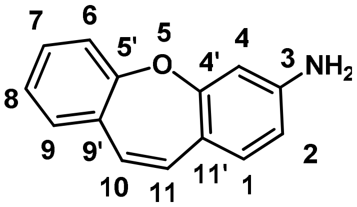

Compound	Angle [°]										
	**NC3C2**	**NC3C4**	**NC3C2C1**	**NC3C4C4'**	**O5C4'C4C3**	**O5C4'C11'C1**	**O5C4'C11'C11**	**C11C11'C4'C4**	**C11C11'C1C2**	**C5'O5C4'C11'**	**C4'O5C5'C9'**
**1f**	121.1	120.7	177.4	-177.4	-173.2	172.1	-7.8	178.6	-178.7	**64.9**	**65.9**
**1a**	121.0	120.7	176.3	-176.5	-173.5	172.7	-6.8	179.6	-179.4	**64.4**	**67.3**
**1c**	121.1	120.7	177.4	-177.4	-173.2	172.1	-7.6	179.0	-179.0	**64.9**	**66.7**
**1e**	121.1	120.7	177.4	-177.5	-173.1	172.1	-7.8	178.7	-178.8	**64.7**	**66.2**
**1h**	121.2	120.6	177.5	-177.5	-173.4	172.2	-7.9	178.6	-178.6	**62.6**	**62.9**
**aniline**	NC1C2<br>120.9	NC1C6<br>120.9	NC1C2C3<br>180	NC1C6C5<br>0.0	-	-	-	-	-		

- The calculated coordinates of (***E*-2f**) (the part of calculated log file) -

```
Standard orientation:
 ---------------------------------------------------------------------
 Center     Atomic     Atomic              Coordinates (Angstroms)
 Number     Number      Type              X           Y           Z
 ---------------------------------------------------------------------
    1          6           0       -7.328920   -2.043166    1.862103
    2          6           0       -8.138204   -0.943905    2.134217
    3          6           0       -7.943091    0.243592    1.443265
    4          6           0       -6.937024    0.373413    0.473154
    5          6           0       -6.147704   -0.756060    0.209864
    6          6           0       -6.332478   -1.946913    0.895740
    7          6           0       -6.751496    1.652090   -0.204459
    8          6           0       -5.617355    2.135324   -0.744814
    9          6           0       -4.313139    1.488910   -0.774150
   10          6           0       -4.144246    0.096531   -0.738371
   11          8           0       -5.232408   -0.759959   -0.838184
   12          6           0       -3.147526    2.277287   -0.835634
   13          6           0       -1.883782    1.722725   -0.831632
   14          6           0       -1.743235    0.328836   -0.773107
   15          6           0       -2.884727   -0.476236   -0.730466
   16          7           0       -0.512876   -0.364081   -0.771336
   17          7           0        0.512658    0.360958   -0.772457
   18          6           0        1.743225   -0.331581   -0.773310
   19          6           0        2.884331    0.474057   -0.731318
   20          6           0        4.144138   -0.098042   -0.738240
   21          6           0        4.313757   -1.490380   -0.772399
   22          6           0        3.148460   -2.279442   -0.833574
   23          6           0        1.884418   -1.725475   -0.830468
   24          8           0        5.231759    0.759017   -0.838752
   25          6           0        6.147228    0.756880    0.209084
   26          6           0        6.937021   -0.371802    0.474031
   27          6           0        6.752131   -1.651565   -0.201632
   28          6           0        5.618398   -2.136023   -0.741688
   29          6           0        6.331598    1.948885    0.893137
   30          6           0        7.328077    2.047071    1.859208
```

31	6	0	8.137855	0.948544	2.132952
32	6	0	7.943133	-0.240060	1.443880
33	1	0	-8.922524	-1.013804	2.878097
34	1	0	-8.574746	1.099848	1.652819
35	1	0	-5.706924	-2.795650	0.647518
36	1	0	-7.612666	2.312965	-0.185639
37	1	0	-5.638654	3.152695	-1.122753
38	1	0	-3.257684	3.355131	-0.882534
39	1	0	-1.003011	2.347893	-0.878183
40	1	0	-2.781346	-1.553931	-0.715059
41	1	0	2.780417	1.551717	-0.717121
42	1	0	3.259156	-3.357280	-0.879357
43	1	0	1.003958	-2.351111	-0.876650
44	1	0	7.613588	-2.312031	-0.181667
45	1	0	5.640331	-3.153892	-1.118254
46	1	0	5.705591	2.796924	0.643684
47	1	0	8.922238	1.019941	2.876618
48	1	0	8.575141	-1.095761	1.654634
49	1	0	-7.478487	-2.977930	2.388998
50	1	0	7.477287	2.982714	2.384645

After PCM corrections, the SCF energy is -1338.08051424 a.u.

Visualization of calculated geometry of (***E*-2f**)

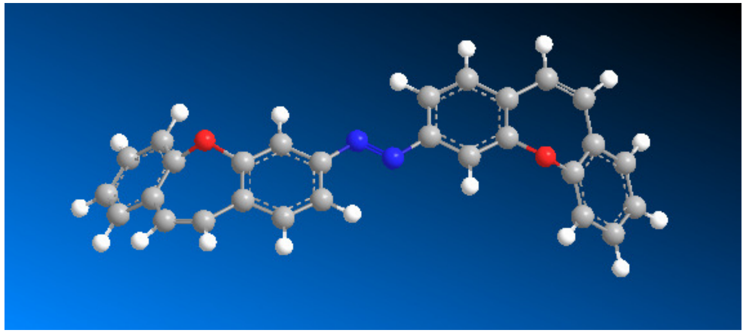

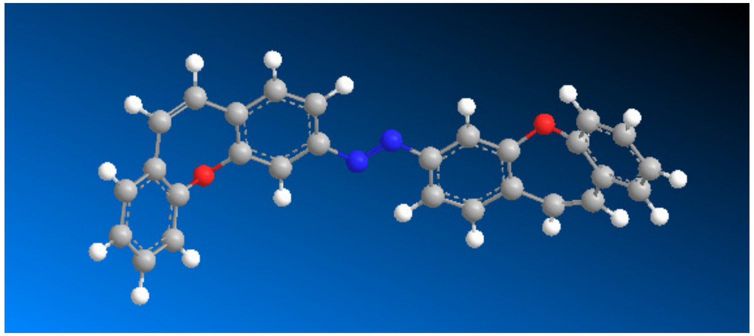

- The calculated coordinates of (**Z-2f**) (the part of calculated log file) -

Standard orientation:

Center Number	Atomic Number	Atomic Type	Coordinates (Angstroms) X	Y	Z
1	6	0	7.169710	0.365357	-1.367856
2	6	0	7.541249	1.192695	-0.312150
3	6	0	6.723969	1.294168	0.804743
4	6	0	5.516308	0.585713	0.900336
5	6	0	5.176772	-0.251802	-0.171952
6	6	0	5.983975	-0.359054	-1.294224
7	6	0	4.676945	0.751776	2.082795
8	6	0	3.343691	0.597384	2.167597
9	6	0	2.428472	0.222534	1.096681
10	6	0	2.818898	-0.531849	-0.021810
11	8	0	4.079025	-1.105466	-0.096658
12	6	0	1.087994	0.636116	1.152585
13	6	0	0.176417	0.315369	0.162101
14	6	0	0.597081	-0.432624	-0.940243
15	6	0	1.932396	-0.823034	-1.043587
16	7	0	-0.232942	-0.689898	-2.078556
17	7	0	-1.401714	-1.107645	-2.006181
18	6	0	-1.986145	-1.600856	-0.792785
19	6	0	-1.402715	-2.662407	-0.096521
20	6	0	-2.092276	-3.264370	0.946008
21	6	0	-3.348535	-2.800811	1.310538
22	6	0	-3.957176	-1.734590	0.632662
23	6	0	-3.269066	-1.167054	-0.447784
24	6	0	-5.262450	-1.250435	1.075733
25	6	0	-5.755750	-0.006845	0.945375
26	6	0	-5.110753	1.142145	0.316611
27	6	0	-4.152913	1.018513	-0.700070
28	8	0	-3.872597	-0.225060	-1.261866
29	6	0	-5.447634	2.441279	0.726387
30	6	0	-4.845192	3.559086	0.166479

31	6	0	-3.889707	3.403592	-0.833703
32	6	0	-3.545021	2.127423	-1.268667
33	1	0	8.467335	1.752859	-0.356863
34	1	0	7.013672	1.937146	1.628608
35	1	0	5.685213	-1.030105	-2.090320
36	1	0	5.190519	1.125539	2.963247
37	1	0	2.869827	0.858969	3.108570
38	1	0	0.762487	1.226176	2.002035
39	1	0	-0.846384	0.658152	0.236517
40	1	0	2.271865	-1.387937	-1.903110
41	1	0	-0.430004	-3.029952	-0.397820
42	1	0	-1.647848	-4.099309	1.473763
43	1	0	-3.878853	-3.265369	2.133939
44	1	0	-5.845481	-1.961990	1.651874
45	1	0	-6.706231	0.203996	1.425883
46	1	0	-6.195895	2.563107	1.501827
47	1	0	-5.125062	4.549591	0.504181
48	1	0	-2.826981	1.980074	-2.066044
49	1	0	-3.422273	4.270311	-1.285400
50	1	0	7.803984	0.271226	-2.241211

After PCM corrections, the SCF energy is -1338.05417174 a.u.

Visualization of calculated geometry of (***Z*-2f**)

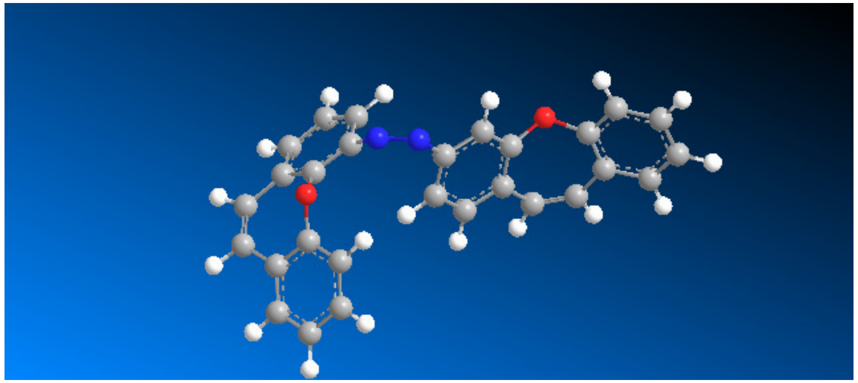

- The calculated coordinates for (**3a-3d**) compounds are in our publication - M. Wrzesiński, D. Mielecki, P. Szczeciński, E. Grzesiuk, H. Krawczyk, *Tetrahedron* 72 (2016) 3877-3884 and the calculated coordinates of (**3e)** is presented:

- The calculated coordinates of (**3e**)(the part of calculated log file) -

Standard orientation:

Center Number	Atomic Number	Atomic Type	Coordinates (Angstroms) X	Y	Z
1	6	0	-3.781824	-0.285578	0.192762
2	6	0	-4.038498	1.056305	0.474751
3	6	0	-3.044170	1.987932	0.209395
4	6	0	-1.794998	1.615408	-0.326999
5	6	0	-1.591931	0.247332	-0.618264
6	6	0	-2.566867	-0.703673	-0.351743
7	6	0	-0.777290	2.636441	-0.550739
8	6	0	0.564060	2.463020	-0.563870
9	6	0	1.307469	1.219954	-0.384488
10	6	0	0.769127	-0.042961	-0.669651
11	8	0	-0.467036	-0.178854	-1.302449
12	6	0	2.626398	1.273156	0.114034
13	6	0	3.375089	0.120036	0.341881
14	6	0	2.805007	-1.140352	0.039674
15	6	0	1.506594	-1.203927	-0.461016
16	8	0	4.637747	0.100392	0.854958
17	6	0	5.263317	1.344961	1.153342
18	8	0	3.470843	-2.313228	0.276574
19	6	0	4.636957	-2.554619	-0.526373
20	7	0	-4.815214	-1.282656	0.459212
21	8	0	-5.883818	-0.896722	0.941728
22	8	0	-4.569076	-2.462346	0.192030
23	1	0	-4.994545	1.350666	0.888760
24	1	0	-3.226359	3.037022	0.425190
25	1	0	-2.393132	-1.745031	-0.591046
26	1	0	-1.151297	3.656016	-0.613897

27	1	0	1.176048	3.360139	-0.639026
28	1	0	3.045303	2.247195	0.341545
29	1	0	1.081161	-2.170462	-0.709352
30	1	0	6.253317	1.094095	1.537073
31	1	0	5.366028	1.966844	0.255369
32	1	0	4.705103	1.898448	1.918672
33	1	0	4.999939	-3.542394	-0.234889
34	1	0	4.374311	-2.562917	-1.591567
35	1	0	5.410707	-1.805564	-0.340229

```
After PCM corrections, the SCF energy is  -1048.26621520     a.u.
```

Visualization of calculated geometry of (**3e)**

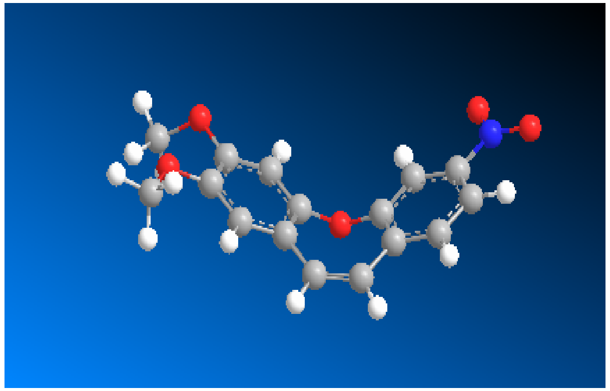

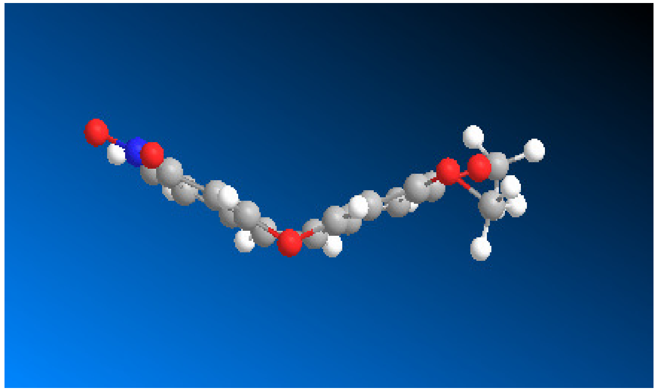

- The calculated coordinates of (**5a**)(the part of calculated log file) -

Standard orientation:

Center Number	Atomic Number	Atomic Type	Coordinates (Angstroms) X	Y	Z
1	6	0	5.853200	2.802880	0.446189
2	6	0	6.632332	2.920973	-0.701616
3	6	0	6.098585	2.465174	-1.899221
4	6	0	4.818885	1.880454	-1.984950
5	6	0	4.074589	1.754526	-0.788368

6	6	0	4.582010	2.229546	0.415623
7	6	0	4.323093	1.489038	-3.298460
8	6	0	3.329272	0.625186	-3.590555
9	6	0	2.508267	-0.168647	-2.682978
10	6	0	2.342468	0.114136	-1.315428
11	8	0	2.764096	1.315615	-0.751101
12	6	0	1.835669	-1.286227	-3.203367
13	6	0	1.021469	-2.073605	-2.403858
14	6	0	0.853364	-1.801549	-1.041776
15	6	0	1.559560	-0.714091	-0.489225
16	6	0	-0.150790	-2.683158	-0.284062
17	6	0	0.163448	-2.999382	1.184164
18	6	0	-1.615831	-2.290979	-0.533588
19	6	0	-0.718605	-2.648201	2.214455
20	6	0	-0.471104	-2.962651	3.550303
21	6	0	0.689983	-3.646284	3.911881
22	6	0	1.592406	-4.011669	2.920552
23	6	0	1.313836	-3.708067	1.585256
24	6	0	-2.510408	-3.334480	-0.812080
25	6	0	-3.841331	-3.093400	-1.117633
26	6	0	-4.345627	-1.784270	-1.190389
27	6	0	-3.455812	-0.730605	-0.922357
28	6	0	-2.113621	-0.971029	-0.562062
29	6	0	-5.747476	-1.593634	-1.552114
30	6	0	-6.540965	-0.529334	-1.312137
31	6	0	-6.188634	0.715224	-0.638119
32	6	0	-4.866195	1.204287	-0.537621
33	8	0	-3.795695	0.594862	-1.167754
34	6	0	-7.218642	1.498362	-0.080292
35	6	0	-6.970985	2.709833	0.551365
36	6	0	-5.653855	3.157594	0.615829
37	6	0	-4.598667	2.421609	0.076825
38	8	0	1.378892	-0.407802	0.834868
39	6	0	2.527717	-0.547761	1.680594
40	7	0	6.368993	3.303072	1.719914
41	8	0	7.498864	3.797750	1.730956
42	8	0	5.649667	3.201785	2.717081
43	8	0	-1.275418	0.090601	-0.361051
44	6	0	-1.420522	0.795943	0.878633
45	7	0	-5.362689	4.441507	1.255125
46	8	0	-6.307079	5.076405	1.730425
47	8	0	-4.189126	4.818731	1.288578
48	7	0	2.317520	-4.188894	0.620621
49	8	0	3.501944	-4.126639	0.957342
50	8	0	1.933859	-4.660479	-0.450658
51	1	0	7.615807	3.370398	-0.650256
52	1	0	6.678992	2.568966	-2.811621

53	1	0	3.982790	2.191327	1.314929
54	1	0	4.868366	1.920013	-4.135143
55	1	0	3.148466	0.428569	-4.645442
56	1	0	1.954418	-1.525125	-4.256624
57	1	0	0.494565	-2.915800	-2.843684
58	1	0	-0.059444	-3.650812	-0.782339
59	1	0	-1.629665	-2.120037	1.959798
60	1	0	-1.191862	-2.671073	4.309075
61	1	0	0.890383	-3.896338	4.949029
62	1	0	2.506497	-4.542925	3.158876
63	1	0	-2.146763	-4.358673	-0.796098
64	1	0	-4.507833	-3.925317	-1.328078
65	1	0	-6.216392	-2.461575	-2.011447
66	1	0	-7.588805	-0.615927	-1.590973
67	1	0	-8.240710	1.138146	-0.156112
68	1	0	-7.769877	3.304883	0.975133
69	1	0	-3.589566	2.809646	0.105110
70	1	0	2.175292	-0.359429	2.696671
71	1	0	2.942734	-1.558914	1.619984
72	1	0	3.306275	0.172617	1.425459
73	1	0	-0.847018	1.717919	0.769050
74	1	0	-2.468234	1.033671	1.084281
75	1	0	-1.001708	0.214906	1.704848

After PCM corrections, the SCF energy is -2341.12490420 a.u.

Visualization of calculated geometry of (**5a**):

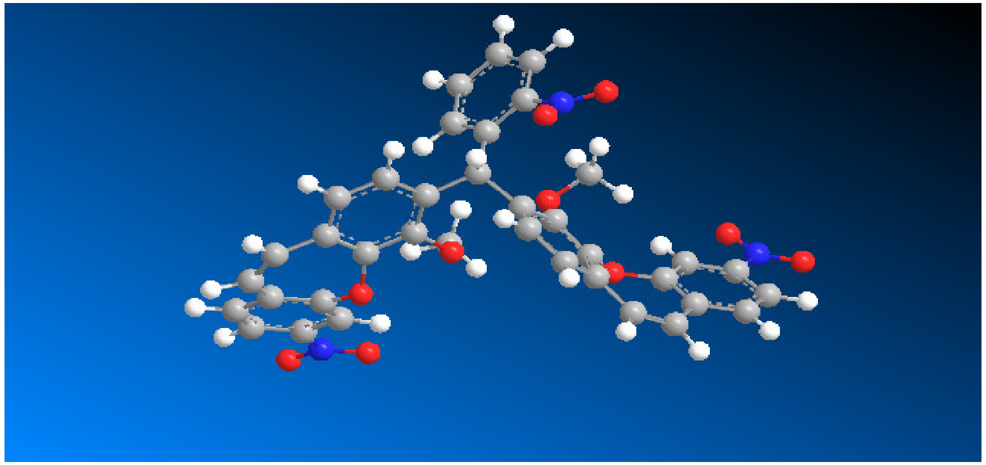

- The calculated coordinates of (**5b**) (the part of calculated log file) -

Standard orientation:

Center Number	Atomic Number	Atomic Type	Coordinates (Angstroms) X	Y	Z
1	6	0	6.126427	-1.635286	-0.283147
2	6	0	6.697047	-2.556714	0.594969
3	6	0	5.916004	-3.622367	1.029036
4	6	0	4.579834	-3.786843	0.612703
5	6	0	4.046889	-2.816641	-0.263135
6	6	0	4.806798	-1.746740	-0.713333
7	6	0	3.816933	-4.947175	1.073564
8	6	0	2.474354	-5.050309	1.198378
9	6	0	1.465201	-4.031136	0.909701
10	6	0	1.673000	-3.001134	-0.022731
11	8	0	2.798723	-2.976011	-0.833785
12	6	0	0.205734	-4.095756	1.534500
13	6	0	-0.804259	-3.182605	1.235092
14	6	0	-0.586551	-2.150094	0.316122
15	6	0	0.674608	-2.068809	-0.307540
16	6	0	-1.654743	-1.161732	-0.152321
17	6	0	-3.010022	-1.851151	-0.360770
18	6	0	-1.739975	0.075878	0.743858
19	6	0	-3.221209	-2.563351	-1.553715
20	6	0	-4.418216	-3.226410	-1.798380
21	6	0	-5.417756	-3.177254	-0.826715
22	6	0	-5.246703	-2.481052	0.367518
23	6	0	-4.042080	-1.817266	0.589765
24	6	0	-1.646229	0.009913	2.141430
25	6	0	-1.742899	1.151060	2.929574
26	6	0	-1.896647	2.424184	2.353878
27	6	0	-1.976033	2.490191	0.954096
28	6	0	-1.907041	1.342089	0.153172
29	6	0	-1.954804	3.616239	3.201797
30	6	0	-1.543832	4.863325	2.884327

31	6	0	-0.922391	5.271062	1.626695
32	6	0	-1.191355	4.633138	0.396975
33	8	0	-2.213496	3.695043	0.310072
34	6	0	-0.011486	6.344606	1.619682
35	6	0	0.644970	6.739319	0.462512
36	6	0	0.386564	6.042497	-0.717869
37	6	0	-0.528419	4.989103	-0.771122
38	7	0	6.936037	-0.521179	-0.787957
39	8	0	8.124851	-0.475539	-0.459447
40	8	0	6.367554	0.348021	-1.452525
41	8	0	0.896195	-1.138979	-1.290761
42	6	0	1.512434	0.078232	-0.858670
43	8	0	-1.938333	1.487070	-1.211800
44	6	0	-3.246869	1.572507	-1.794993
45	7	0	1.106611	6.426923	-1.935418
46	8	0	1.879555	7.385087	-1.864724
47	8	0	0.909513	5.767663	-2.956530
48	7	0	-6.684248	-3.874495	-1.070957
49	8	0	-7.533073	-3.864719	-0.176575
50	8	0	-6.836709	-4.432919	-2.159375
51	1	0	7.724534	-2.437803	0.911067
52	1	0	6.344836	-4.357516	1.703689
53	1	0	4.397622	-1.040660	-1.423399
54	1	0	4.418364	-5.786169	1.415385
55	1	0	2.085699	-5.968607	1.633314
56	1	0	0.015654	-4.895792	2.244295
57	1	0	-1.778117	-3.294270	1.701409
58	1	0	-1.337526	-0.809846	-1.134315
59	1	0	-2.433462	-2.599011	-2.301174
60	1	0	-4.586787	-3.771210	-2.718459
61	1	0	-6.045449	-2.461343	1.097883
62	1	0	-3.915419	-1.256709	1.509169
63	1	0	-1.490120	-0.951372	2.620336
64	1	0	-1.677084	1.066548	4.010659
65	1	0	-2.293460	3.444777	4.221499
66	1	0	-1.572392	5.622242	3.662336
67	1	0	0.190103	6.864312	2.551816
68	1	0	1.347638	7.561473	0.460788

69	1	0	-0.742076	4.473749	-1.697878
70	1	0	1.543637	0.731691	-1.732225
71	1	0	0.930975	0.558628	-0.064719
72	1	0	2.530806	-0.101761	-0.495892
73	1	0	-3.092483	1.705813	-2.867402
74	1	0	-3.818110	0.654415	-1.618356
75	1	0	-3.798088	2.430848	-1.395317

After PCM corrections, the SCF energy is -2341.14529953 a.u.

Visualization of calculated geometry of (**5b**):

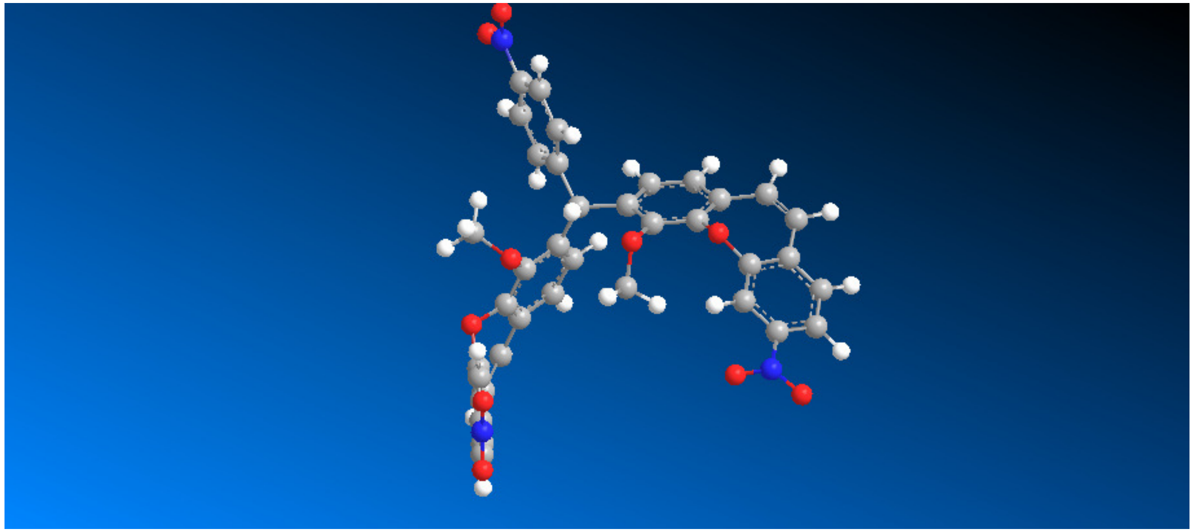

- The calculated coordinates of (**5c**) (the part of calculated log file)

Standard orientation:

Center Number	Atomic Number	Atomic Type	Coordinates (Angstroms) X	Y	Z
1	6	0	8.109562	-0.253596	0.593617
2	6	0	8.691121	-1.255469	-0.179466
3	6	0	7.947387	-1.792539	-1.222128

4	6	0	6.634640	-1.367135	-1.506505
5	6	0	6.085394	-0.353497	-0.689800
6	6	0	6.816419	0.207786	0.349150
7	6	0	5.927670	-1.969694	-2.631897
8	6	0	4.594684	-2.038505	-2.829555
9	6	0	3.524509	-1.542019	-1.967772
10	6	0	3.700244	-0.511565	-1.024729
11	8	0	4.864686	0.239803	-0.964222
12	6	0	2.247598	-2.115210	-2.074067
13	6	0	1.184229	-1.681922	-1.288080
14	6	0	1.352970	-0.655315	-0.355959
15	6	0	2.636207	-0.087321	-0.215855
16	6	0	0.226002	-0.129641	0.537780
17	6	0	-0.201223	1.308240	0.208221
18	6	0	-0.942028	-1.111632	0.672543
19	6	0	0.148860	1.901493	-1.015234
20	6	0	-0.216982	3.197118	-1.352642
21	6	0	-0.977286	3.935560	-0.447639
22	6	0	-1.369471	3.400225	0.768395
23	6	0	-0.964004	2.106052	1.086564
24	6	0	-0.846129	-2.112655	1.650975
25	6	0	-1.841130	-3.063454	1.823072
26	6	0	-3.010244	-3.035717	1.042565
27	6	0	-3.109993	-2.034817	0.064995
28	6	0	-2.090995	-1.088178	-0.140421
29	6	0	-4.072549	-4.010770	1.287654
30	6	0	-5.398728	-3.857605	1.078326
31	6	0	-6.073010	-2.666646	0.569315
32	6	0	-5.435555	-1.733689	-0.275153
33	8	0	-4.186928	-2.010504	-0.811991
34	6	0	-7.409691	-2.408474	0.931065
35	6	0	-8.076732	-1.267606	0.503801
36	6	0	-7.392705	-0.363114	-0.307701
37	6	0	-6.073469	-0.579287	-0.703983
38	8	0	2.805135	0.961026	0.656449
39	6	0	3.414194	0.622227	1.909734
40	7	0	8.877740	0.348310	1.685364
41	8	0	10.009950	-0.088636	1.902503

42	8	0	8.350340	1.256258	2.331688
43	8	0	-2.252463	-0.095967	-1.074456
44	6	0	-2.229842	-0.477890	-2.459119
45	7	0	-8.080051	0.842672	-0.774664
46	8	0	-9.242326	1.023922	-0.405241
47	8	0	-7.459554	1.613018	-1.510386
48	7	0	-1.375540	5.308032	-0.781340
49	8	0	-2.039293	5.930899	0.047199
50	8	0	-1.023415	5.754646	-1.873506
51	7	0	-1.392452	1.643961	2.421889
52	8	0	-0.658222	0.876585	3.045834
53	8	0	-2.455689	2.083096	2.855625
54	1	0	9.698655	-1.590211	0.031885
55	1	0	8.388033	-2.564962	-1.845999
56	1	0	6.400580	1.016212	0.935507
57	1	0	6.565008	-2.478026	-3.351983
58	1	0	4.253731	-2.597338	-3.698669
59	1	0	2.092485	-2.916032	-2.791845
60	1	0	0.217644	-2.161785	-1.391730
61	1	0	0.648690	-0.063244	1.541151
62	1	0	0.726268	1.322301	-1.725451
63	1	0	0.075165	3.636047	-2.298797
64	1	0	-1.966057	3.976071	1.462477
65	1	0	0.034337	-2.142816	2.286599
66	1	0	-1.734599	-3.826401	2.589210
67	1	0	-3.749235	-4.928007	1.775970
68	1	0	-6.055933	-4.658678	1.409533
69	1	0	-7.926010	-3.121213	1.567853
70	1	0	-9.103759	-1.072408	0.785083
71	1	0	-5.560319	0.123615	-1.346686
72	1	0	4.374772	0.118282	1.765755
73	1	0	3.569109	1.565299	2.437180
74	1	0	2.758046	-0.024763	2.505950
75	1	0	-2.417155	0.440005	-3.019819
76	1	0	-1.247304	-0.876407	-2.736408
77	1	0	-3.007345	-1.211987	-2.681888

After PCM corrections, the SCF energy is -2545.63225077 a.u.

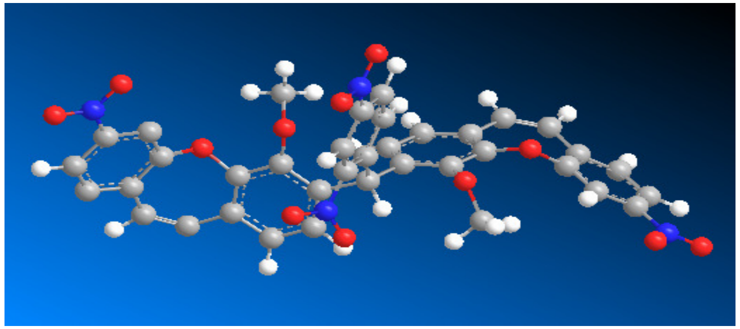

Visualization of calculated geometry of (**5c**):

- The calculated coordinates of (**5d**) (the part of calculated log file)

Standard orientation:

Center Number	Atomic Number	Atomic Type	Coordinates (Angstroms) X	Y	Z
1	6	0	6.205035	-2.460147	-1.033567
2	6	0	7.371274	-1.723179	-1.227011
3	6	0	7.472394	-0.484109	-0.608606
4	6	0	6.438070	0.046391	0.188483
5	6	0	5.270202	-0.733982	0.348267
6	6	0	5.154014	-1.981816	-0.251292
7	6	0	6.633132	1.343627	0.825454
8	6	0	5.684727	2.199475	1.262385
9	6	0	4.233786	2.057657	1.195093
10	6	0	3.574676	0.824620	1.078718
11	8	0	4.257415	-0.379497	1.220135
12	6	0	3.432852	3.211883	1.250247
13	6	0	2.051708	3.130316	1.197745
14	6	0	1.384317	1.902697	1.057292
15	6	0	2.171056	0.737914	0.984744
16	6	0	-0.152954	1.965019	1.147773
17	6	0	-0.829208	2.929062	0.155338
18	6	0	-0.929671	0.665040	1.416370
19	6	0	-1.897353	3.685251	0.669382
20	6	0	-2.600024	4.613092	-0.093872
21	6	0	-2.234701	4.827148	-1.421985
22	6	0	-1.178489	4.100831	-1.965607
23	6	0	-0.495344	3.163288	-1.187699
24	6	0	-0.928784	0.195963	2.736492
25	6	0	-1.639964	-0.930211	3.121329
26	6	0	-2.465257	-1.598065	2.203341

27	6	0	-2.500682	-1.114577	0.884734
28	6	0	-1.704435	-0.033422	0.463646
29	6	0	-3.268963	-2.732488	2.655427
30	6	0	-4.469252	-3.141734	2.187838
31	6	0	-5.247642	-2.503811	1.132795
32	6	0	-4.647878	-1.752866	0.098420
33	8	0	-3.271807	-1.773704	-0.072913
34	6	0	-6.652907	-2.605138	1.137455
35	6	0	-7.436976	-1.963707	0.188088
36	6	0	-6.800601	-1.203472	-0.792707
37	6	0	-5.411434	-1.089226	-0.851253
38	17	0	0.795436	2.281335	-2.007391
39	8	0	1.599490	-0.507579	0.913778
40	6	0	1.565362	-1.079582	-0.397207
41	7	0	6.078011	-3.778947	-1.653620
42	8	0	5.037192	-4.411987	-1.460429
43	8	0	7.016971	-4.186474	-2.341771
44	8	0	-1.676200	0.415095	-0.822768
45	6	0	-1.699326	-0.489699	-1.933584
46	7	0	-7.606484	-0.517239	-1.802185
47	8	0	-8.833405	-0.623394	-1.732226
48	8	0	-7.018045	0.133322	-2.669375
49	1	0	8.171822	-2.121161	-1.837260
50	1	0	8.379456	0.100092	-0.735131
51	1	0	4.273396	-2.586606	-0.082958
52	1	0	7.668363	1.671861	0.886202
53	1	0	6.028262	3.158131	1.646298
54	1	0	3.914344	4.180741	1.352337
55	1	0	1.463166	4.041097	1.267542
56	1	0	-0.285096	2.495294	2.099107
57	1	0	-2.180795	3.530525	1.707924
58	1	0	-3.420429	5.169379	0.350257
59	1	0	-2.761354	5.553507	-2.034232
60	1	0	-0.880910	4.246792	-2.998798
61	1	0	-0.330257	0.722390	3.475751
62	1	0	-1.595831	-1.279753	4.148955
63	1	0	-2.877137	-3.247260	3.530802
64	1	0	-4.965998	-3.955669	2.711292

65	1	0	-7.131071	-3.195339	1.914050
66	1	0	-8.516763	-2.040016	0.197885
67	1	0	-4.941570	-0.511561	-1.635811
68	1	0	1.115184	-2.068595	-0.284283
69	1	0	0.960265	-0.468134	-1.072138
70	1	0	2.571444	-1.184952	-0.817455
71	1	0	-1.123767	0.002537	-2.720661
72	1	0	-1.237114	-1.447769	-1.680994
73	1	0	-2.718158	-0.669452	-2.282655

After PCM corrections, the SCF energy is -2596.21338922 a.u.

Visualization of calculated geometry of (**5d**):

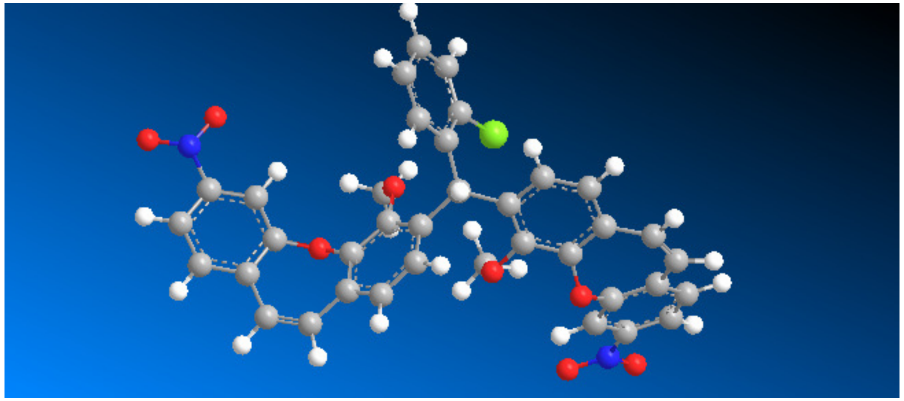

- The calculated coordinates of (**5e**) (the part of calculated log file) -

Standard orientation:

Center Number	Atomic Number	Atomic Type	Coordinates (Angstroms) X	Y	Z
1	6	0	6.954919	-1.848911	0.317117
2	6	0	7.324183	-2.244434	-0.967118
3	6	0	6.757490	-1.580396	-2.046977

4	6	0	5.823190	-0.538879	-1.880797
5	6	0	5.481290	-0.172154	-0.559431
6	6	0	6.040941	-0.817693	0.535190
7	6	0	5.270921	0.115943	-3.061769
8	6	0	4.093149	0.768979	-3.161934
9	6	0	3.088019	0.980788	-2.124380
10	6	0	3.381955	0.969677	-0.751667
11	8	0	4.692562	0.932498	-0.289877
12	6	0	1.751838	1.211099	-2.498893
13	6	0	0.754597	1.415165	-1.556404
14	6	0	1.039300	1.389724	-0.185351
15	6	0	2.374566	1.161875	0.208770
16	6	0	0.012070	1.721402	0.900174
17	6	0	-0.766621	3.014187	0.595888
18	6	0	-0.860158	0.591660	1.477426
19	6	0	-0.225390	4.205036	1.113100
20	6	0	-0.814178	5.451819	0.927476
21	6	0	-1.999736	5.525629	0.201279
22	6	0	-2.577319	4.380585	-0.336512
23	6	0	-1.957031	3.145552	-0.135735
24	6	0	-1.351358	0.804559	2.777930
25	6	0	-2.144429	-0.125436	3.431627
26	6	0	-2.508899	-1.328433	2.801956
27	6	0	-2.005522	-1.553654	1.514020
28	6	0	-1.189199	-0.622967	0.847151
29	6	0	-3.401673	-2.267985	3.478138
30	6	0	-4.275299	-3.128884	2.909408
31	6	0	-4.526769	-3.299716	1.481488
32	6	0	-3.548412	-3.027325	0.501444
33	8	0	-2.239963	-2.766659	0.874843
34	6	0	-5.789882	-3.736548	1.036146
35	6	0	-6.084831	-3.866236	-0.314581
36	6	0	-5.093142	-3.554177	-1.244928
37	6	0	-3.821862	-3.133729	-0.854512
38	35	0	-2.817488	1.650669	-0.962216
39	17	0	-2.780300	7.079579	-0.048083
40	7	0	-5.384814	-3.686253	-2.672967
41	8	0	-6.515790	-4.053348	-3.000420

42	8	0	-4.485804	-3.421966	-3.474691
43	8	0	2.731980	1.239153	1.536504
44	6	0	2.744840	-0.007483	2.241677
45	7	0	7.548021	-2.521201	1.473429
46	8	0	8.347360	-3.436222	1.262484
47	8	0	7.213877	-2.141774	2.598470
48	8	0	-0.801883	-0.882694	-0.441439
49	6	0	0.203730	-1.891454	-0.613621
50	1	0	8.040820	-3.044339	-1.103975
51	1	0	7.039251	-1.868428	-3.055934
52	1	0	5.797766	-0.499827	1.540430
53	1	0	5.849719	-0.016110	-3.973356
54	1	0	3.807136	1.121518	-4.151130
55	1	0	1.502200	1.230100	-3.556594
56	1	0	-0.263910	1.577877	-1.885784
57	1	0	0.636390	2.000215	1.753198
58	1	0	0.699500	4.148979	1.681633
59	1	0	-0.363529	6.345935	1.344753
60	1	0	-3.496559	4.440670	-0.906889
61	1	0	-1.098175	1.732276	3.285038
62	1	0	-2.506244	0.079023	4.435696
63	1	0	-3.418330	-2.187897	4.563609
64	1	0	-4.936324	-3.686390	3.569625
65	1	0	-6.552926	-3.969326	1.773695
66	1	0	-7.058267	-4.197659	-0.653418
67	1	0	-3.056271	-2.910704	-1.585849
68	1	0	3.140398	0.208237	3.236635
69	1	0	1.733278	-0.421182	2.333098
70	1	0	3.390821	-0.739258	1.743439
71	1	0	0.368990	-1.963818	-1.690244
72	1	0	1.139188	-1.592908	-0.126452
73	1	0	-0.130653	-2.856100	-0.223246

After PCM corrections, the SCF energy is -5167.33306634 a.u.

Visualization of calculated geometry of (**5e**):

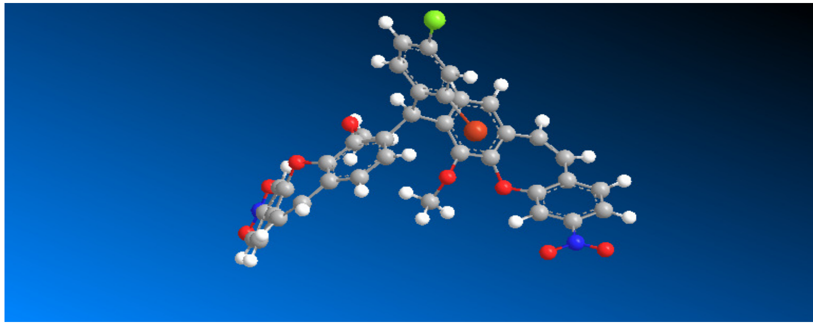

2. Experimental section

EXPERIMENTAL SECTION

General information

All the spectra were recorded using a Varian VNMRS spectrometer operating at 11.7 T and Varian Mercury VX 9.4 T magnetic field. Measurements were performed for ca. 1.0 M solutions of all the compounds in DMSO-d_6 or CDCl3. The residual signals of DMSO-d6 (2.54 ppm) and CDCl3 (7.26 ppm) in ^{1}H NMR and of the DMSO-d_6 signal (40.4 ppm) and of $CDCl_3$ (77.0 ppm) in 13C NMR spectra were used as the chemical shift references. Spin multiplicities are described as s (singlet), d(doublet), t (triplet), q (quartet), m (multiplet), dd (double doublet). Coupling constants are reported in Hertz. All the proton spectra were recorded using the standard spectrometer software and parameters set: acquisition time 3s, pulse angle 30o. The standard measurement parameter set for 13C NMR spectra was: pulse width 7 µs (the 90o pulse width was 12.5 µs), acquisition time 1 s, spectral width 200 ppm, 1000 scans of 32 K data point were accumulated and after zero-filling to 64 K; and the FID signals were subjected to Fourier transformation after applying a 1 Hz line broadening. The ^{1}H-^{13}Cgs-HSQC and ^{1}H-^{13}Cgs-HMBC spectra were also recorded using the standard Varian software.

The dibenzo [*b, f*] oxepines (**3a-3f**) were obtained using the procedure described by us in Tetrahedron (H. Krawczyk, M. Wrzesiński, D. Mielecki, P. Szczeciński, E. Grzesiuk), Synthesis of derivatives of methoxydibenzo[b,f]oxepine in the presence of sodium azide. Tetrahedron 2016, 72, 3877-3884. The compounds (**1a-1h**) were obtained in standard reduction of nitro group with zinc in acetic acid.

General procedure of synthesis of compounds (2a-2h) (Scheme 1 in the text):

To a solution of appropriate aminodibenzo[*b,f*]oxepine (1 mmol) dissolved in dichloromethane (15 mL) DBU was added (2 mmol). The resulting solution was stirred for 5 min at room temperature and next cooled down to a -78°C by acetone/dry ice bath. Afterward, the NBS (2 mmol) was added as a solid to the reaction mixture. The solution turned dark red and was stirred for 10 min at -78°C before quenching by addition of saturated bicarbonate solution. The mixture was transferred to a separation funnel and the organic layer was separated, washed with 50 mL of water and 50 mL of 1M HCl, dried over anhydrous sodium sulfate, and concentrated to dryness in a *vacuum*. The residue was subjected to column chromatography on silica gel using DCM as a mobile phase.

Characterisation Date of (2a-2h)

2a. yield 45%; time of reaction 15 min; dark orange/red powder mp = 193°C; ^{1}H NMR (500 MHz, $CDCl_3$, 298 K): δ (ppm): 7.88 (2H, d, JH_2,H_4 = 2 Hz, H_4), 7.71 (2H, dd, JH_1,H_2 = 8.5 Hz, H_2), 7.30 (2H, d, H_1), 7.07 (2H, t, $JH_8,H_7,9$ = 8.5 Hz, H_8), 6.96 (2H, dd, JH_7,H_9 = 1.5 Hz, H_7), 6.82 (2H, AB spin system, d, JH_{10},H_{11} = 11.5 Hz, H_{10}), 6.79 (2H, dd, H_9), 6.79 (2H, AB spin system, d, H_{11}), 3.98 (6H, s, OCH_3). ^{13}C NMR (125 MHz, $CDCl_3$, 298 K): δ (ppm): 157.74, 153.84, 151.91, 145.06, 133.61, 131.62, 131.36, 129.55, 129.34, 124.97, 120.78, 119.93, 116.24, 112.85, 56.31. HRMS (ESI): m/z calculated for $C_{30}H_{23}N_2O_4$+H 475.16523, found: 475.16524.

2b. yield 27%; time of reaction 15 min; orange powder mp = 250°C; ^{1}H NMR (500 MHz, CDC13, 298 K): 7.72 (2H, dd, JH_8,H_9 = 8 Hz, JH_6,H_8 = 2 Hz, H_8), 7.71 (2H, d, H_6), 7.27 (2H, d, H_9), 7.08 (2H, d, JH_1,H_2 = 8.5 Hz, H_1), 6.80 (2H, d, JH_2,H_4 = 2.5 Hz, H_4), 6.71 (2H, AB spin system, d, JH_{10},H_{11} = 11.5 Hz, H_{11}), 6.70 (2H, dd, H_2), 6.61 (2H, AB spin system, d, H_{11}), 3.84 (6H, s, OCH_3). ^{13}C NMR (125 MHz, CDC13, 298 K): δ (ppm): 161.89, 158.02, 157.28, 153.61, 133.80, 131.31, 130.23, 129.55, 127.04, 123.11, 121.19, 114.58, 111.26, 106.99, 55.57. HRMS (ESI): m/z calculated for $C_{30}H_{23}N_2O_4$+H 475.16523, found: 475.16498.

2c. yield 35%; time of reaction 15 min; orange powder mp = 251.5°C; ^{1}H NMR (500 MHz, $CDCl_3$, 298 K): δ(ppm): 7.72 (2H, dd, $J_{H6,H8}$ = 2 Hz, $J_{H8,H9}$ = 8.5 Hz, H_8), 7.72 (2H, d, H_6), 7.30 (2H, d, H_9), 7.17 (2H, d, $J_{H3,H4}$ = 9 Hz, H_4), 6.85 (2H, dd, $J_{H1,H3}$ = 3 Hz, H_3), 6.77 (2H, AB spin system, d, $J_{H10,H11}$ = 11.5 Hz, H_{11}), 6.74 (2H, AB spin system, d, H_{10}), 6.70 (2H, d, H_1), 3.78 (6H, s, OCH_3). ^{13}C NMR (125 MHz, $CDCl_3$, 298 K): δ(ppm): 158.16, 156.69, 153.83, 147.06, 133.34, 131.36, 130.93, 129.82, 129.75, 123.96, 120.93, 119.09, 115.41, 114.06, 55.68. HRMS (ESI): *m/z* calculated for $C_{30}H_{23}N_2O_4$+H 475.16523, found: 475.16523.

2d. yield 37%; time of reaction 15 min; orange powder, decomposition above 258°C; ^{1}H NMR (500 MHz, $CDCl_3$, 298 K): δ(ppm): 7.97 (2H, d, $J_{H6,H8}$ = 2 Hz, H_6), 7.73 (2H, dd, $J_{H8,H9}$ = 8.5 Hz, H_8), 7.50 (2H, d, H_9), 6.64 (2H, t, $J_{H3,H2,4}$ = 8.5 Hz, H_3), 6.21 (2H, AB spin system, d, $J_{H10,H11}$ = 11.5 Hz, H_{11}), 6.17 (2H, AB spin system, d, H_{10}), 5.76 (2H, d, H_2), 5.65 (2H, d, H_4), 3.93 (6H, s, OCH_3). ^{13}C NMR (125 MHz, $CDCl_3$, 298 K): δ(ppm): 157.44, 157.14, 154.31, 153.74, 134.20, 134.03, 129.69, 129.47, 127.50, 118.63, 118.51, 117.36, 107.72, 107.11, 56.58. HRMS (ESI): *m/z* calculated for $C_{30}H_{23}N_2O_4$+H 475.16523, found: 475.16523.

2e. yield 15 %; time of reaction 15 min; dark orange/red powder mp = 254°C; ^{1}H NMR (500 MHz, $CDCl_3$, 298 K): δ(ppm): δ (ppm): 7.73 (2H, d, $J_{H6,H8}$ = 2 Hz, H_6), 7.71 (2H, dd, $J_{H8,H9}$ = 8 Hz, H_8), 7.28 (1H, d, H_9), 6.81 (2H, s, H_4), 6.70 (2H, AB spin system, d, $J_{H10,H11}$ = 11.5 Hz, H_{11}), 6.67 (2H, AB spin system, d, H_{10}), 6.64 (2H, s, H_1), 3.92 (6H, s, OCH_3), 3.86 (6H, s, OCH_3).^{13}C NMR (125 MHz, $CDCl_3$, 298 K): δ(ppm): 157.64, 153.70, 150.75, 150.64, 146.16, 133.73, 131.31, 129.58, 127.83, 121.97, 121.21, 114.25, 111.27, 105.37, 56.29, 56.14. HRMS (ESI): *m/z* calculated for $C_{32}H_{27}N_2O_6$+H 535.18636, found: 535.18601.

2f. yield 10 %; time of reaction 15 min; orange powder decomposition above 255°C; ^{1}H NMR (500 MHz, $CDCl_3$, 298 K): δ (ppm): 7.74 (2H, m, H_4), 7.71 (2H, dd, $J_{H1,H2}$ = 8.5 Hz, $J_{H2,H4}$ = 2.5 Hz, H_2), 7.32 (2H, ddd, $J_{H6,H7}$ = 8 Hz, $J_{H7,H8}$ = 7.5 Hz, $J_{H7,H9}$ = 2 Hz, H_7), 7.30 (2H, d, H_1), 7.24 (2H, dd, $J_{H6,H8}$ = 1.5 Hz, H_6), 7.19 (2H, dd, $J_{H8,H9}$ = 7.5 Hz, H_9), 7.14 (2H, ddd, H_8), 6.79 (2H, AB spin system, d, $J_{H10,H11}$ = 11.5 Hz, H_{10}), 6.74 (2H, AB spin system, d, H_{11}).^{13}C NMR (125 MHz, $CDCl_3$, 298 K): δ (ppm): 158.09, 157.20, 154.04, 133.70, 131.78, 130.58, 130.53, 129.91, 129.70, 129.57, 125.36, 121.77, 121.29, 114.89. HRMS (ESI): *m/z* calculated for $C_{28}H_{19}N_2O_2$+H 415.14410, found: 415.04201.

2g. yield 25 %; time of reaction 15 min; yellow powder mp = 251.5°C; ^{1}H NMR (500 MHz, $CDCl_3$, 298 K): 7.73 (2H, dd, $J_{H1,H2}$ = 8 Hz, $J_{H2,H4}$ = 2 Hz, H_2), 7.69 (2H, d, H_4), 7.29 (2H, d, H_1), 7.17 (2H, d, $J_{H8,H9}$ = 8.5 Hz, H_9), 7.03 (2H, d, $J_{H6,H8}$ = 2 Hz, H_6), 6.91 (1H, dd, H_8), 6.75 (2H, AB spin system, d, $J_{H10,H11}$ = 11.5 Hz, H_{10}), 6.72 (2H, AB spin system, d, H_{11}), 2.30 (6H, s, CH_3). ^{13}C NMR (125 MHz, DMSO-d6, 298 K): δ (ppm): 168.99,

157.44, 157.29, 153.79, 152.18, 133.29, 130.68, 129.75, 129.70, 129.16, 128.17, 121.51, 118.41, 115.24, 114.50, 21.11. HRMS (ESI): *m/z* calculated for $C_{32}H_{23}N_2O_6$+H 531.15506, found: 531.15505.

2h. yield 23 %; time of reaction 15 min; orange powder mp = 248°C; ^{1}H NMR (500 MHz, $CDCl_3$): δ(ppm): 8.32 (2H, d, $J_{H1,H2}$ = 3 Hz, H_1), 8.28 (2H, dd, $J_{H3,H4}$ = 9 Hz, H_3), 8.23 (2H, d, $J_{H6,H8}$ = 2.5 Hz, H_6), 8.09 (2H, dd, $J_{H8,H9}$ = 8.5 Hz, H_8), 7.67 (2H, d, H_4), 7.62 (2H, d, H_9), 7.16 (2H, AB spin system, d, $J_{H10,H11}$ = 11.5 Hz, H_{11}), 7.08 (2H, AB spin system, d, H_{10}). ^{13}C NMR (125 MHz, $CDCl_3$): δ(ppm): 160.43, 155.24, 148.60, 145.08, 136.35, 131.67, 130.67, 130.62, 130.24, 126.22, 125.28, 123.04, 121.06, 117.00. HRMS (ESI): *m/z* calculated for $C_{28}H_{16}N_4O_6$+H 505.11426, found: 531.11426.

General procedure of synthesis of compounds (4a-4e) (Scheme 2 in the text):

To a solution of appropriate methoxy derivative of nitrodibenzo[*b,f*]oxepine (1 mmol) dissolved in dichloromethane (15 mL) paraformaldehyde (1 mmol) was added. Afterward, the catalyst - $BF_3{\cdot}Et_2O$ (0.3 mL) was added carefully to a reaction mixture. The resulting solution turned orange and was stirred for 7 hours at room temperature. Afterward, the reaction mixture was quenched by the addition of methanol (10 mL). The mixture was transferred to a separation funnel filled with 20 mL of water or was filtered under reduced pressure if a precipitate has formed. The water layer was washed twice with 20 mL of dichloromethane. Organic layers were dried over anhydrous magnesium sulfate and concentrated to dryness in vacuo. The residue was subjected to column chromatography on silica gel using DCM as a mobile phase.

Characterisation Date of (4a-4e)

4a. Characterization and spectra can be found in P. Tobiasz, M. Poterała, E. Jaśkowska, H. Krawczyk, *RSC Adv.*, 2018, 8, 30678-30682.

4b. a mixture of 5 products; time of reaction 3h.

4c4. yield 54 %; time of reaction 2h; dark yellow/orange powder decomposition above 295°C; ^{1}H NMR (500 MHz, $CDCl_3$): δ(ppm): rings A: 8.08 (1H, d, $J_{H6,H8}$ = 2.5 Hz, H_6), 8.01 (1H, dd, $J_{H8,H9}$ = 8.5 Hz, H_8), 7.27 (1H, d, H_9), 7.24 (1H, d, $J_{H3,H4}$ = 8.5 Hz, H_4), 6.99 (1H, d, H_3), 6.92 (1H, AB spin system, d, $J_{H10,H11}$ = 11.5 Hz, H_{11}), 6.72 (1H, AB spin system, d, H_{10}), 3.79 (3H, s, OCH_3), rings B: 7.95 (1H, dd, $J_{H6,H8}$ = 2.5 Hz, $J_{H8,H9}$ = 8.5 Hz, H_8), 7.93 (1H, d, H_6), 7.26 (1H, d, H_9), 6.93 (1H, s, H_4), 6.84 (1H, AB spin system, d, $J_{H10,H11}$ = 11.5 Hz, H_{11}), 6.70 (1H, AB spin system, d, H_{10}), 3.84 (3H, s, OCH_3), rings C: 7.93 (1H, dd, $J_{H6,H8}$ = 2 Hz, $J_{H8,H9}$ = 8.5 Hz, H_8), 7.80 (1H, d, H_6), 7.24 (1H, d, H_9), 6.81 (1H, AB spin system, d, $J_{H10,H11}$ = 11.5 Hz, H_{11}), 6.67 (1H, AB spin system, d, H_{10}), 6.64 (1H, s, H_1), 6.51 (1H, s, H_4), 3.89 (3H, s, OCH_3), 4.02 (4H, s, CH_2). ^{13}C NMR (125 MHz, $CDCl_3$): δ(ppm): 157.91, 157.22, 157.17, 155.44, 154.86, 154.84, 154.45, 151.85, 150.30, 150.07, 148.45, 148.39, 137.38, 137.34, 137.26, 133.66, 133.58, 131.55, 131.50, 131.27, 129.78, 129.47, 129.42, 129.25, 128.47, 128.07, 127.89, 127.62, 126.01, 123.08, 122.05, 121.40, 120.13, 120.04, 119.89, 116.86, 116.71, 115.92, 114.21, 112.64, 110.68, 110.20, 56.15, 55.87, 55.85, 25.55, 25.55. HRMS (ESI): *m/z* calculated for $C_{47}H_{32}N_3O_{12}$ 830.19859, found: 830.19805.

4d. yield 65 %; time of reaction 2,5 h; light yellow powder mp = 236.5°C; ^{1}H NMR (500 MHz, $CDCl_3$, 298 K): δ (ppm): 7.93 (2H, dd, $J_{H8,H9}$ = 8.5 Hz, $J_{H6,H8}$ = 2 Hz, H_8), 7.72 (2H, d, H_6), 7.27 (2H, AB spin system, d, $J_{H10,H11}$ = 11.5 Hz, H_{11}), 7.27 (2H, d, H_9), 7.03 (2H, d, $J_{H3,H4}$ = 8.5 Hz, H_3), 6.76 (2H, AB spin system, d, H_{10}), 6.59 (2H, d, H_4), 4.45 (2H, s, CH_2), 3.81 (6H, s, OCH_3). ^{13}C NMR (125 MHz, $CDCl_3$, 298 K): δ (ppm): 156.72 (C5'), 156.38 (C1), 156.20 (C4'), 148.05 (C7), 138.45 (C9'), 132.06 (C3), 129.14 (C11), 129.14 (C9), 127.08 (C10), 124.61 (C2), 120.07 (C8), 119.42 (C11'), 117.34 (C6), 107.29 (C4), 55.97 (OCH_3), 29.69 (CH_2). HRMS (ESI): *m/z* calculated for $C_{31}H_{22}N_2O_8$ 550.13761, found: 550.13707.

4e. mixture of Prins reaaction products, yield 30 % raw; time of reaction 3,5 h.

General procedure of synthesis of compounds (5a-5f) (Table 2 in the text):

To a solution of 6-methoxy-3-nitrodibenzo[*b,f*]oxepine (1 mmol) dissolved in dichloromethane (C_{max}) a proper aldehyde (0.5 mmol) was added. Afterward, the catalyst - $BF_3{\cdot}Et_2O$ (0.3 mL) was added carefully to a reaction mixture. The resulting solution turned brown and was stirred for 3 weeks at room temperature. Afterward, the reaction mixture was quenched by the addition of methanol (10 mL). The mixture was transferred to a separation funnel filled with 20 mL of water. The water layer was washed twice with 20 mL of dichloromethane. Organic layers were dried over anhydrous magnesium sulfate and concentrated to dryness in vacuo. The residue was subjected to column chromatography on silica gel using ethyl acetate/hexane 1:1 as a mobile phase.

Characterisation Date of (5a-5f)

5a. yield 26 %; time of reaction 3 weeks; light yellow powder mp = 167°C; ^{1}H NMR (500 MHz, CDCl3, 298 K): δ (ppm): 8.19 (2H, d, $J_{H6,H8}$ = 2.5 Hz, H_6), 8.00 (1H, dd, $J_{H14,H15}$ = 8.5 Hz, $J_{H14,H16}$ = 2 Hz, H_{14}), 8.00 (2H, d, $J_{H8,H9}$ = 8.5 Hz, H_8), 7.34-7.32 (1H, m, H_{16}), 7.30 (2H, d, H_9), 7.11 (2H, d, $J_{H1,H2}$ = 8.5 Hz, H_1), 7.03 (2H, AB spin system, d, $J_{H10,H11}$ = 11.5 Hz, H_{11}), 6.93-6.83 (2H, m, H_{15}, H_{17}), 6.82 (2H, AB spin system, d, H_{10}), 6.67 (2H, d, H_2), 6.66 (1H, s, CH), 3.94 (6H, s, OCH_3). ^{13}C NMR of compound (5a): (125 MHz, CDCl3, 298 K): δ (ppm): 157.53 (C5'), 151.51 (C4), 148.56 (C13), 147.18 (C7), 146.10 (C4'), 145.21 (C12), 137.36 (C9'), 135.40 (C16), 133.20 (C17), 130.31 (C10),129.98 (C11) 128.84 (C3), 128.66 (C11'), 128.52 (C9), 126.56 (C15), 124.21 (C2), 120.11 (C14), 120.05 (C8), 117.45 (C6), 112.81 (C1), 56.14 (OCH_3), 40.78 (CH). HRMS (ESI): *m/z* calculated for $C_{37}H_{25}N_3O_{10}$+H 672.16127, found: 672.16127.

5b. yield 36 %; time of reaction 2 weeks; yellow powder mp = 145°C; ^{1}H NMR (500 MHz, $CDCl_3$, 298 K): δ (ppm): 8.19 (2H, d, $J_{H6,H8}$ = 2.5 Hz, H_6), 8.12 (2H, d, $J_{H13,H14}$ = 8.5 Hz, H_{14}), 8.01 (2H, dd, $J_{H8,H9}$ = 8.5 Hz, H_8), 7.30 (2H, d, H_9), 7.13 (2H, d, H_{13}), 6.87 (2H, d, $J_{H1,H2}$ = 8.5 Hz, H_1), 6.79 (2H, AB spin system, d, $J_{H10,H11}$ = 11.5 Hz, H_{11}), 6.75 (2H, AB spin system, d, H_{10}), 6.45 (2H, d, H_2), 5.79 (1H, s, CH), 3.94 (6H, s, OCH_3). ^{13}C NMR (125 MHz, $CDCl_3$, 298 K): δ (ppm): 157.46 (C5'), 151.00 (C4), 150.00 (C12), 148.68 (C7), 146.88 (C15), 146.20 (C4'), 137.06 (C9'), 131.32 (C3), 130.38 (C13), 129.72 (C11), 129.36 (C10), 129.12 (C11'), 129.03 (C9), 127.00 (C2), 123.80 (C14), 120.18 (C8), 117.49 (C6), 112.52 (C1), 56.14 (OCH_3), 49.27 (CH). HRMS (ESI): *m/z* calculated for $C_{37}H_{25}N_3O_{10}$ 671.15399, found: 671.15345.

5c. yield 66%; time of reaction 2 weeks; light yellow powder mp = 185.5°C; ^{1}H NMR (500 MHz, $CDCl_3$, 298 K): δ (ppm): 8.77 (1H, d, $J_{H14,H16}$ = 2.5 Hz, H_{14}), 8.24 (1H, dd, $J_{H16,H17}$ = 8.5 Hz, H_{16}), 8.18 (2H, d, $J_{H6,H8}$ = 2.5 Hz, H_6), 8.01 (2H, dd, $J_{H8,H9}$ = 8.5 Hz, H_8), 7.31 (2H, d, H_9), 7.11 (1H, d, H_{17}), 6.84 (2H, d, $J_{H1,H2}$ = 8.5 Hz, H_1), 6.82 (2H, AB spin system, d, $J_{H10,H11}$ = 11.5 Hz, H_{11}), 6.79 (2H, AB spin system, d, H_{10}), 6.66 (1H, s, CH), 6.36 (2H, d, H_2), 3.94 (6H, s, OCH_3).^{13}C NMR (125 MHz, $CDCl_3$, 298 K): δ (ppm): 157.47 (C5'), 151.44 (C4), 148.91 (C13), 148.74 (C7), 146.87

(C15), 146.36 (C4'), 144.12 (C12), 136.86 (C9'), 133.24 (C17), 129.87 (C10), 129.49 (C3), 129.40 (C11'), 129.19 (C9), 129.04 (C11), 126.69 (C16), 126.51 (C2), 120.75 (C14), 120.26 (C8), 117.47 (C6), 112.51 (C1), 56.14 (OCH_3), 44.42 (CH).HRMS (ESI): *m/z* calculated for $C_{37}H_{23}N_4O_{12}$ 715.13124, found: 715.13070.

5d. yield 9%; time of reaction 3 weeks; light yellow powder mp = 141°C; ^{1}H NMR (500 MHz, $CDCl_3$, 298 K): δ (ppm): 8.19 (2H, d, $J_{H6,H8}$ = 2.5 Hz, H_6), 7.99 (2H, dd, $J_{H8,H9}$ = 8.5 Hz, H_8), 7.53 (1H, dd, $J_{H15,H17}$ = 2 Hz, $J_{H16,H17}$ = 8.5 Hz, H_{17}), 7.28 (2H, d, H_9), 7.23-7.19 (1H, m, H_{16}), 6.92 (1H, ddd, $J_{H14,H15}$ = 8.5 Hz, $J_{H15,H16}$ = 8.5 Hz, H_{15}), 6.83 (2H, d, $J_{H1,H2}$ = 8.5 Hz, H_1), 6.81 (2H, AB spin system, d, $J_{H10,H11}$ = 11.5 Hz, H_{11}), 6.73 (2H, AB spin system, d, H_{10}), 6.46 (1H, dd, $J_{H14,H16}$ = 1.5 Hz, H_{14}) 6.45 (2H, d, H_2), 6.00 (1H, s, CH), 3.93 (6H, s, OCH_3). ^{13}C NMR (125 MHz, $CDCl_3$, 298 K): δ (ppm): 157.46, 150.69, 148.54, 146.06, 140.01, 137.32, 131.66, 131.34, 130.10, 129.41, 128.99, 128.84, 128.35, 126.64, 126.30, 125.08, 120.07, 119.06, 117.46, 112.30, 56.06, 46.49. HRMS (ESI): *m/z* calculated for $C_{37}H_{25}ClN_2O_8$+H 661.13721, found: 661.13722.

5e. yield 14%; time of reaction 3 weeks; light yellow powder mp =133.5°C; ^{1}H NMR (500 MHz, $CDCl_3$, 298 K): δ (ppm): 8.19 (2H, d, $J_{H6,H8}$ = 2.5 Hz, H_6), 8.00 (2H, dd, $J_{H8,H9}$ = 8.5 Hz, H_8), 7.60 (1H, d, $J_{H14,H16}$ = 2 Hz, H_{14}), 7.29 (2H, d, H_9), 7.14 (1H, dd, $J_{H16.H17}$ = 8.5 Hz, H_{16}), 6.84 (2H, d, $J_{H1,H2}$ = 8.5 Hz, H_1), 6.78 (2H, AB spin system, d, $J_{H10,H11}$ = 11.5 Hz, H_{11}), 6.75 (2H, AB spin system, d, H_{10}), 6.65 (1H, d, H_{17}), 6.42 (2H, d, H_2), 5.91 (1H, s, CH), 3.93 (6H, s, OCH_3). ^{13}C NMR (125 MHz, $CDCl_3$, 298 K): δ (ppm): 157.47 (C5'), 150.85 (C4), 148.59 (C7), 146.10 (C4'), 140.44 (C12), 137.19 (C9'), 133.56 (C15), 132.85 (C14), 132.16 (C17), 131.15 (C11'), 129.85 (C11), 129.26 (C3), 129.05 (C9), 129.03 (C10), 127.69 (C16), 126.61 (C2), 125.30 (C13), 120.11 (C8), 117.47 (C6), 112.35 (C1), 56.07 (OCH_3), 48.75 (CH). HRMS (ESI): *m/z* calculated for $C_{37}H_{24}BrClN_2O_8$+H 739.04773, found: 739.04773.

5f. yield 43%; time of reaction 3 weeks; light yellow powder mp =266°C; ^{1}H NMR (500 MHz, $CDCl_3$, 298 K): δ (ppm): 8.20 (1H, d, $J_{H6,H8}$ = 2.5 Hz, H_6), 8.18 (1H, d, $J_{H18,H20}$ = 2.5 Hz, H_{18}), 8.01 (1H, dd, $J_{H8,H9}$ = 8.5 Hz, H_8), 7.85 (1H, dd, $J_{H20,H21}$ = 8.5 Hz, H_{20}), 7.33 (d, 1H, H_9), 7.11 (1H, d, H_{21}), 7.09 (1H, AB spin system, d, $J_{H10,H11}$ = 11.5 Hz, H_{11}), 6.91 (1H, t, $J_{H14,H13,15}$ = 8 Hz, H_{14}), 6.91 (1H, d, $J_{H1,H2}$ = 8.5 Hz, H_1), 6.87 (1H, AB spin system, d, H_{10}), 6.82 (1H, dd, $J_{H13,H15}$ = 1 Hz, H_{15}), 6.79 (1H, d, H_2), 6.27 (1H, dd, H_{13}), 4.95 (1H, dd, $J_{H12,H22}$ = 10 Hz, $J_{H12,H22'}$ = 4 Hz, H_{12}), 3.96 (3H, s, OCH_3), 3.95 (3H, s, OCH_3), 3.48 (1H, dd, $J_{H22,H22'}$ = 15 Hz, H_{22}), 3.41 (1H, dd, $H_{22'}$). ^{13}C NMR (125 MHz, $CDCl_3$, 298 K): δ (ppm): 157.52 (C5'), 157.20 (C17'), 151.50 (C16), 150.58 (C4), 148.54 (C7), 147.16 (C19), 146.09 (C4'), 145.20 (C16'), 138.67 (C21'), 137.36 (C9'), 134.95 (C12'), 133.19 (C3), 131.10 (C21), 130.13 (C11), 128.96 (C9), 128.96 (C10), 128.91 (C11'), 125.46 (C2), 125.00 (C14), 121.43 (C13), 120.10 (C8), 119.14 (C20), 117.43 (C6), 117.06 (C18), 112.67 (C1), 110.59 (C15), 56.13 (OCH_3), 56.13 (OCH_3), 40.76 (C12), 37.15 (C22). HRMS (ESI): *m/z* calculated for $C_{30}H_{22}N_2O_8$+H 539.14489, found: 539.14489.

General procedure of synthesis of compounds (6a-6b) (Scheme 3 in the text):

A stirring solution of (*E*)-bis(6-methoxydibenzo[*b,f*]oksepin-3-yl)diazene (1 mmol) dissolved in dichloromethane (C=5 mM) was flushed with argon. After that, a catalyst - $BF_3 \cdot Et_2O$ (0.3 mL) was added carefully to a reaction mixture. Then a proper aldehyde (2 mmol) was added dropwise over 2 hours to a stirring solution. The solution turned dark blue and was stirred for 6 hours at room temperature. Afterward, the reaction mixture was quenched by the addition of methanol (10 mL). The mixture was transferred to a separation funnel filled with 20 mL of water. The water layer was washed twice with 20 mL of dichloromethane. Organic layers were dried over anhydrous magnesium sulfate and concentrated to dryness in vacuo. The residue was subjected to column chromatography on silica gel using DCM as a mobile phase.

Characterisation Date of (**6a, 6b, 7, 8**)

6a. yield 49%; time of reaction 24h; orange powder mp = 240°C; ^{1}H NMR (500 MHz, $CDCl_3$, 298 K): δ (ppm): 7.92 (2H, d, $J_{H2',H4''}$ = 2 Hz, $H_{4''}$), 7.88 (2H, d, $J_{H2,H4}$ = 2 Hz, H_4), 7.73 (2H, dd, $J_{H1',H2'}$ = 8 Hz, $H_{2'}$), 7.72 (2H, dd, $J_{H1,H2}$ = 8 Hz, H_2), 7.32 (2H, d, $H_{1'}$), 7.31 (2H, d, H_1), 7.08 (2H, t, $J_{H8,H7,9}$ = 8 Hz, H_8), 6.97 (2H, dd, $J_{H7,H9}$ = 1.5 Hz, H_9), 6.92 (2H, AB spin system, d, $J_{H10',H11''}$ = 11.5 Hz, $H_{10'}$), 6.87 (2H, AB spin system, d, $H_{11''}$), 6.87 (2H, d, $J_{H8',9''}$ = 8.5 Hz, $H_{8'}$), 6.82 (2H, AB spin system d, $J_{H10,H11}$ = 11.5 Hz, H_{10}), 6.80 (2H, dd, H_7), 6.79 (2H, AB spin system, d, H_{11}), 6.67 (2H, d, $H_{9''}$), 3.98 (6H, s, OCH_3), 3.97 (2H, s, CH_2), 3.96 (6H, s, OCH_3). ^{13}C NMR (125 MHz, $CDCl_3$, 298 K): δ (ppm): 158.25, 157.75, 153.89, 153.84, 151.92, 150.50, 146.14, 145.07, 133.72, 133.65, 131.63, 131.39, 129.92, 129.87, 129.55, 129.49, 129.35, 129.12, 128.53, 126.10, 124.99, 120.80, 119.97, 119.89, 116.24, 116.19, 112.86, 112.54, 56.32, 56.25, 35.53. HRMS (ESI): *m/z* calculated for $C_{61}H_{44}N_4O_8$+H 961.32319, found: 961.32319.

6b. yield 7%; time of reaction 3 weeks; red powder decomposition above 251°C; ^{1}H NMR (500 MHz, $CDCl_3$, 298 K): δ (ppm): 8.78 (1H, d, $J_{H14,H16}$ = 2.5 Hz, H_{14}), 8.24 (1H, dd, $J_{H16,H17}$ = 8.5 Hz, H_{16}), 7.89 (2H, d, $J_{H2',H4''}$ = 2 Hz, $H_{4''}$), 7.88 (2H, d, $J_{H2,H4}$ = 2 Hz, H_4), 7.73 (2H, dd, $J_{H1',H2'}$ = 8 Hz, $H_{2'}$), 7.71 (2H, d, $J_{H1,H2}$ = 8 Hz, H_2), 7.31 (2H, d, $H_{1'}$), 7.29 (2H, d, H_1), 7.14 (1H, d, H_{17}), 7.08 (2H, t, $J_{H8,H7,9}$ = 8 Hz, H_8), 6.97 (2H, dd, $J_{H7,H9}$ = 1.5 Hz, H_9), 6.82 (2H, AB spin system, d, $J_{H10',H11''}$ = 11.5 Hz, $H_{10'}$), 6.82 (2H, AB spin system, d, $J_{H10,H11}$ = 11.5 Hz, H_{10}), 6.79 (2H, dd, H_7), 6.79 (2H, AB spin system, d, $H_{11'}$), 6.75 (1H, s, CH), 6.73 (2H, d, $J_{H8',H9'}$ = 8.5 Hz, $H_{8'}$), 6.72 (2H, AB spin system, H_{11}), 6.33 (2H, d, $H_{9'}$), 3.98 (6H, s, OCH_3), 3.95 (6H, s, OCH_3). ^{13}C NMR (125 MHz, $CDCl_3$, 298 K): δ (ppm): 158.34, 157.74, 154.05, 153.81, 151.90, 151.46, 146.73, 146.66, 145.04, 144.74, 144.22, 135.77, 133.75, 133.42, 131.61, 131.46, 131.07, 130.59, 130.06, 129.61 129.52, 129.37, 129.36, 127.07, 126.55, 126.03, 125.02, 120.81, 120.18, 120.02, 116.22, 115.90, 112.88, 111.97, 56.31, 56.15, 44.56. HRMS (ESI): *m/z* calculated for $C_{67}H_{46}N_6O_{12}$+H 1127.32464, found: 1127.32465.

7. yield 5%; time of reaction 24h; red powder decomposition above 255°C; ^{1}H NMR (500 MHz, $CDCl_3$, 298 K): δ (ppm): 7.92 (4H, d, $J_{H2',H4'}$ = 2 Hz, $H_{4''}$), 7.88 (2H, d, $J_{H2,H4}$ = 2 Hz, H_4), 7.73 (4H, dd, $J_{H1',H2'}$ = 7.5 Hz, $H_{2'}$), 7.72 (2H, dd, $J_{H1,H2}$ = 8 Hz, H_2), 7.32 (4H, d, $H_{1'}$), 7.31 (2H, d, H_1), 7.08 (2H, t, $J_{H8,H7,9}$ = 8 Hz, H_8), 6.97 (2H, dd, $J_{H7,H9}$ = 1.5 Hz, H_9), 6.92 (4H, AB spin system, d, $J_{H10',H11'}$ = 11.5 Hz, $H_{10'}$), 6.87 (4H, AB spin system, d, $H_{11''}$), 6.87 (4H, d, $J_{H8',9'}$ = 8.5 Hz, $H_{8'}$), 6.82 (2H, AB spin system, d, $J_{H10,H11}$ = 11.5 Hz, H_{10}), 6.80 (2H, dd, H_7), 6.79 (2H, AB spin system, d, H_{11}), 6.67 (4H, d, $H_{9''}$), 3.98 (12H, s, OCH_3), 3.97 (4H, s, CH_2), 3.96 (6H, s, OCH_3). HRMS (ESI): *m/z* calculated for $C_{92}H_{67}N_6O_{12}$+H 1447.48114, found: 1447.48115

8. yield 40%; time of reaction 24h; red powder decomposition above 245°C ; ^{1}H NMR (500 MHz, $CDCl_3$, 298 K): δ (ppm): 7.92 (8H, d, $J_{H2',H4'}$ = 2 Hz, $H_{4''}$), 7.88 (2H, d, $J_{H2,H4}$ = 2 Hz, H_4), 7.74 (8H, dd, $J_{H1',H2'}$ = 8 Hz, $H_{2'}$), 7.72 (2H, dd, $J_{H1,H2}$ = 8 Hz, H_2), 7.33 (8H, d, $H_{1'}$), 7.32 (2H, d, H_1), 7.07 (2H, t, $J_{H8,H7,9}$ = 8 Hz, H_8), 6.96 (2H, dd, $J_{H7,H9}$ = 1.5 Hz, H_9), 6.92 (8H, AB spin system, d, $J_{H10',H11'}$ = 11.5 Hz, $H_{10'}$), 6.91 (8H, AB spin system, d, $H_{11''}$), 6.87 (8H, d, $J_{H8',9'}$ = 8.5 Hz, $H_{8'}$), 6.82 (2H, AB spin system, d, $J_{H10,H11}$ = 11.5 Hz, H_{10}), 6.79 (2H, dd, H_7), 6.79 (2H, AB spin system, d, H_{11}), 6.67 (8H, d, $H_{9''}$), 3.98 (6H, s, OCH_3), 3.98 (8H, s, CH_2), 3.96 (24H, s, OCH_3). ^{13}C NMR (125 MHz, $CDCl_3$, 298 K): δ (ppm): 158.29, 157.79, 153.93, 153.88, 151.96, 150.53, 146.22, 145.15, 133.77, 133.73, 131.66, 131.40, 129.94, 129.86, 129.57, 129.55, 129.36, 129.13, 128.54, 126.13, 124.99, 120.84, 119.96, 119.89, 116.25, 116.18, 112.86, 112.63, 56.32, 56.28, 35.54. HRMS (ESI): *m/z* calculated for $C_{154}H_{110}N_{10}O_{20}$+H 2419.79706, found: 2419.79708.

Table S1. **T**he reaction of methoxy derivative 3-nitrodibenzo[*b*,*f*]oxepines with paraformaldehyde and in the presence of a Lewis acid as the catalyst.

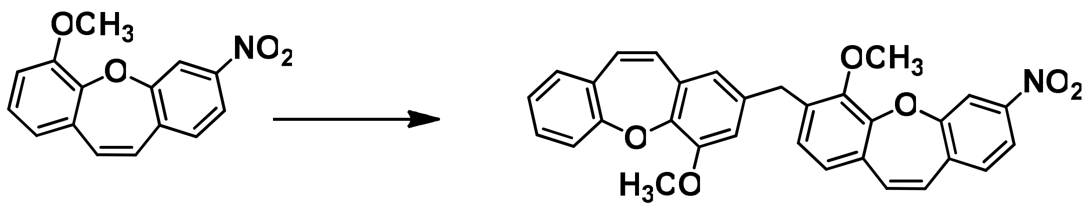

Lewis acid	Conditions	Yield
$BF_3{\cdot}OEt_2$	room temperature, DCM and under argon	**90%**
$ZnCl_2$	room temperature, DCM and under argon	**-**
$AlCl_3$	room temperature, DCM and under argon	**-**
$TiCl_4$	room temperature, DCM and under argon	**mixture**
Scandium triflate	room temperature, DCM and under argon	**mixture**

The structure of (**4c3/4c4**) was determined by gs-HSQC and gs-HMBC spectra. Only one structure of (**4c**) is observed but by NMR spectra is not possible unambiguously to tell which. Based on the chemical shift analysis we postulate the structure of (**4c4**).

4c1

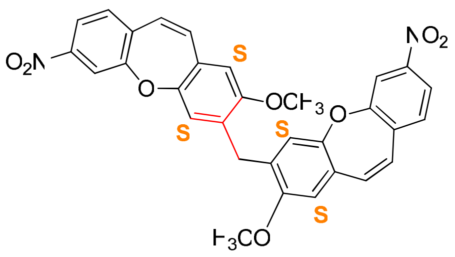

4c2

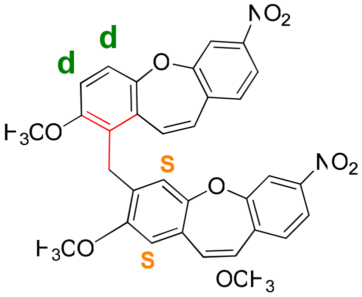

4c3

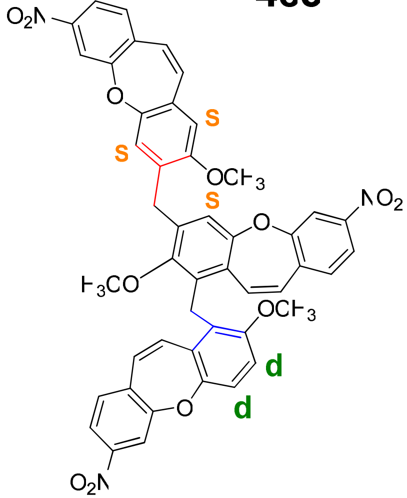

4c4

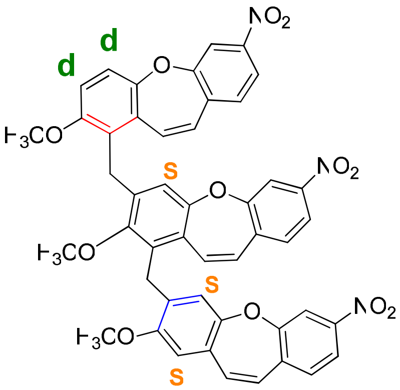

4c5

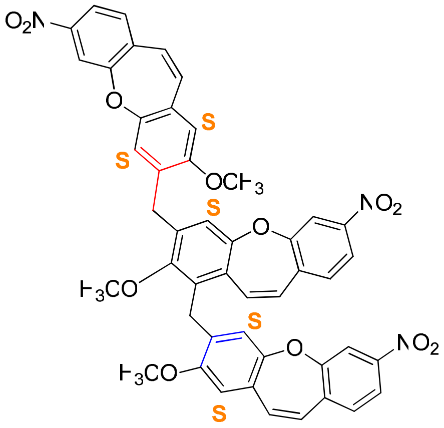

4c6

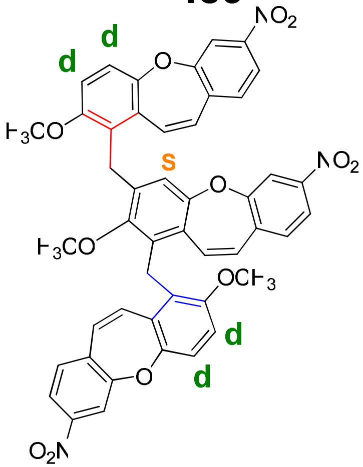

Figure S1. Possible dimmer and trimmer products in the reaction 2-methoxy-7-nitrodibenzo[b,f]oxepine with paraformaldehyde and in the presence of $BF_3{\cdot}OEt_2$ (letter of orange colors- singlet, letter of green color d-doublet in ^{1}H NMR spectrum).

3. NMR spectra of obtained compounds

All the spectra were recorded using a Varian VNMRS spectrometer operating at 11.7 T and Varian Mercury VX 9.4 T magnetic field. Measurements were performed for ca. 1.0 M solutions of all the compounds in DMSO-d_6 or $CDCl_3$. The residual signals of DMSO-d6 (2.54 ppm) and $CDCl_3$ (7.26 ppm) in ^1H NMR and of the DMSO-d_6 signal (40.4 ppm) and of $CDCl_3$ (77.0 ppm) in ^{13}C NMR spectra were used as the chemical shift references. Spin multiplicities are described as s (singlet), d(doublet), t (triplet), q (quartet), m (multiplet), dd (double doublet). Coupling constants are reported in Hertz. All the proton spectra were recorded using the standard spectrometer software and parameters set: acquisition time 3s, pulse angle 30°. The standard measurement parameter set for ^{13}C NMR spectra was: pulse width 7 μs (the 90° pulse width was 12.5 μs), acquisition time 1 s, spectral width 200 ppm, 1000 scans of 32 K data point were accumulated and after zero-filling to 64 K; and the FID signals were subjected to Fourier transformation after applying a 1 Hz line broadening. The ^1H-^{13}Cgs-HSQC and ^1H-^{13}Cgs-HMBC spectra were also recorded using the standard Varian software.

Copies of ^{1}H NMR and ^{13}C NMR spectra of (2a-h)

^{1}H NMR of compound (2a):

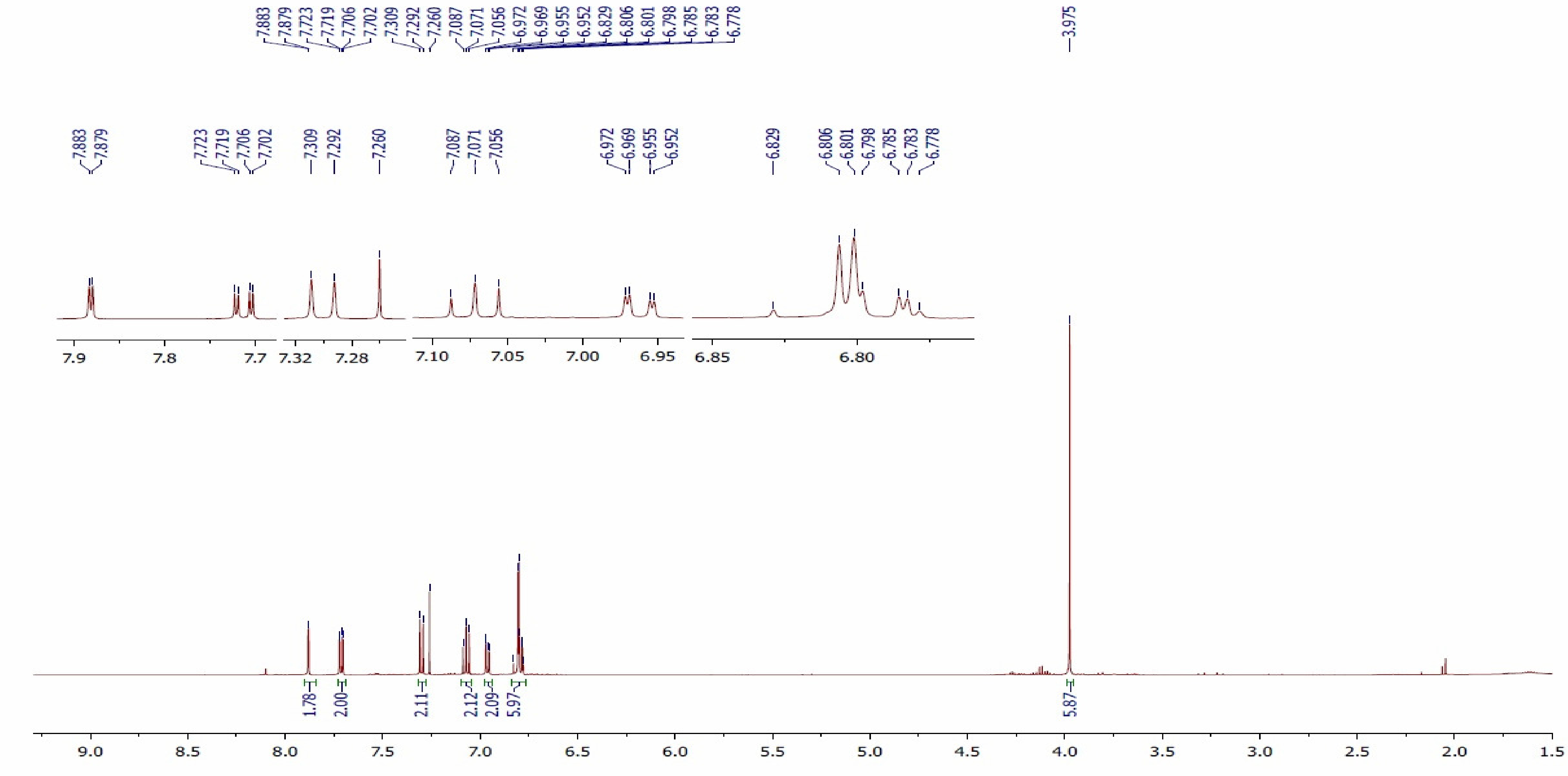

^{13}C NMR of compound (2a):

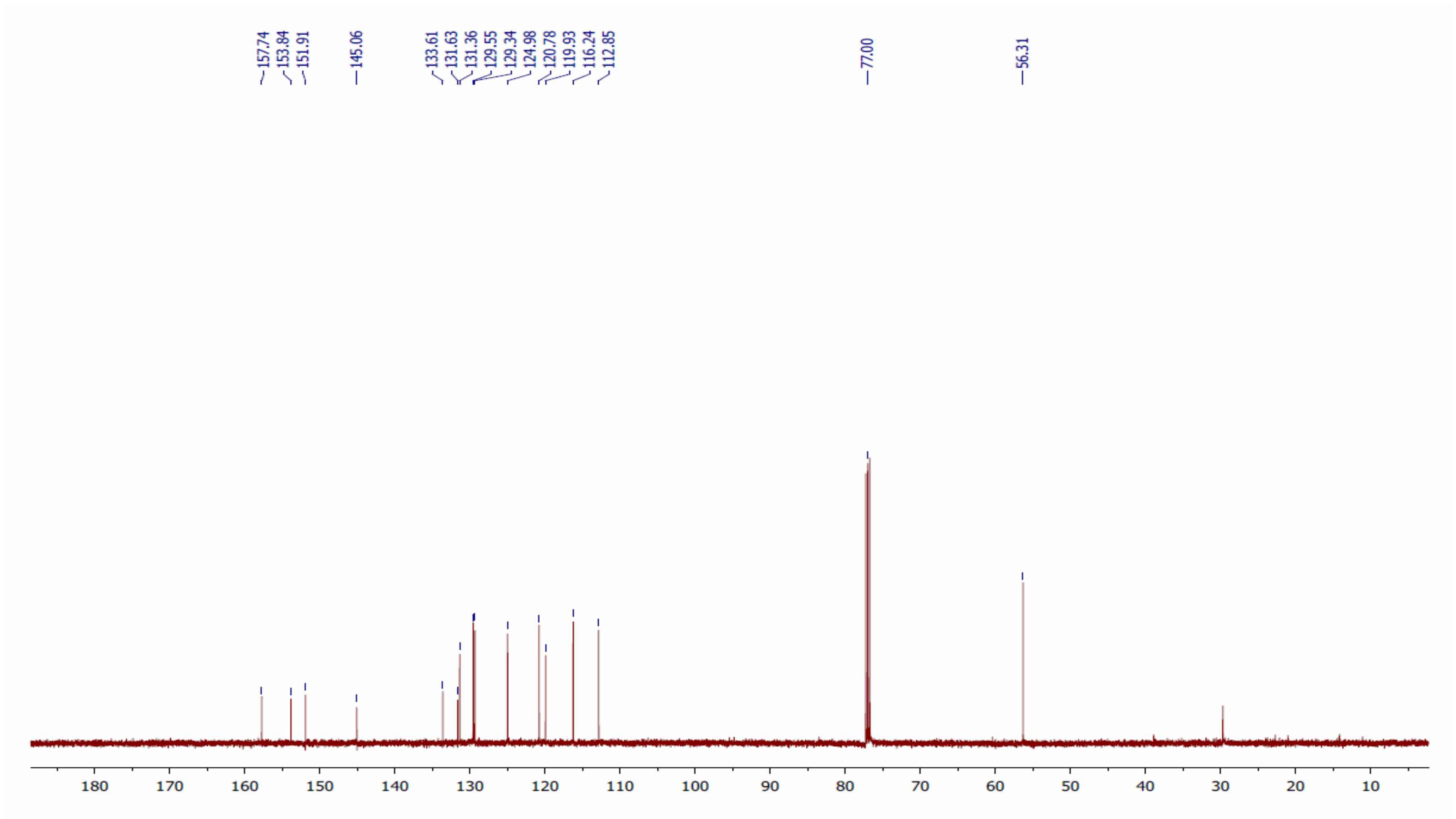

^{1}H NMR of compound (2b):

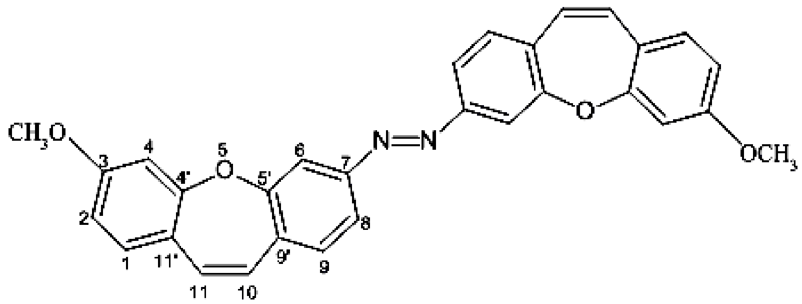

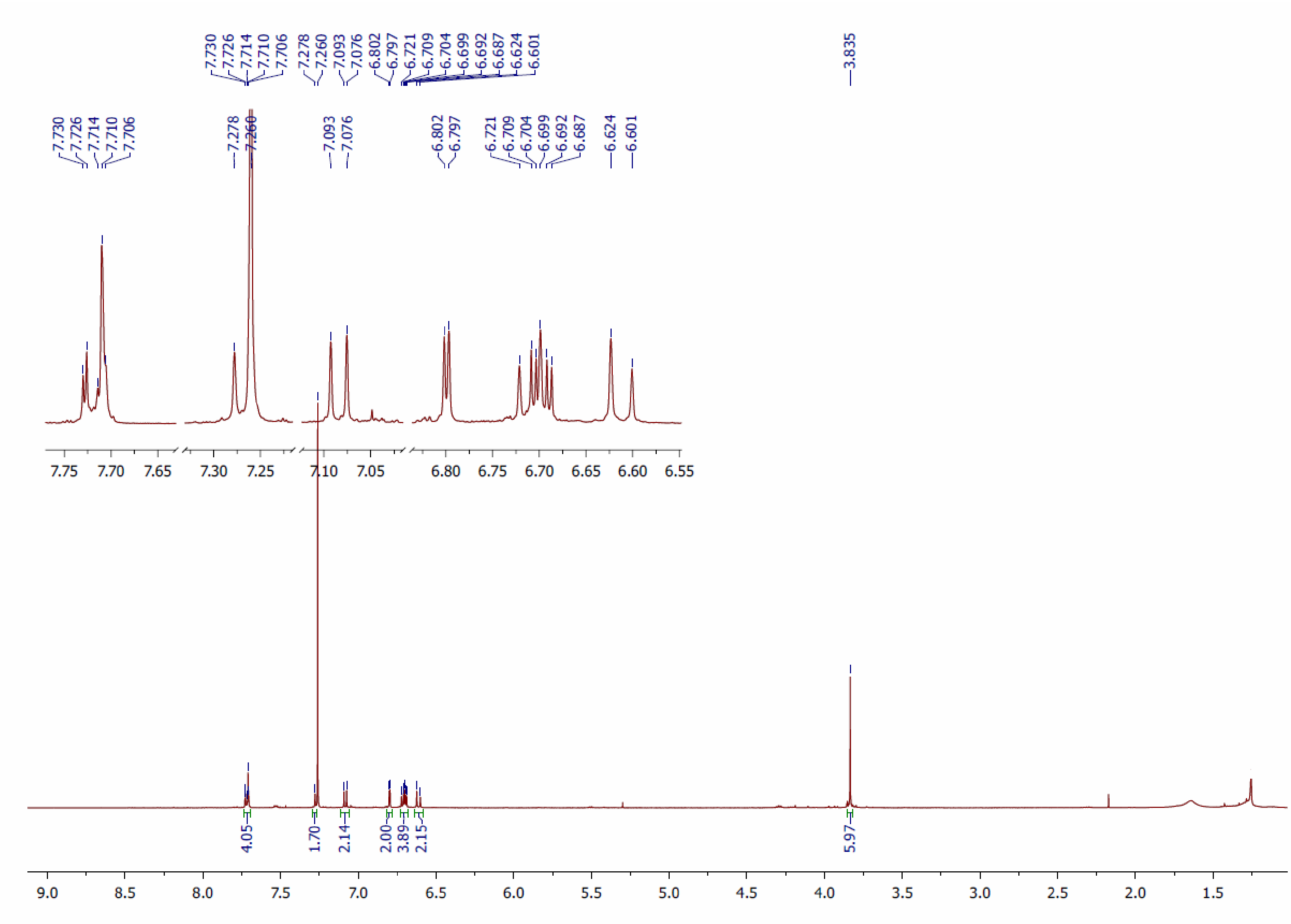
7.730
7.726
7.714
7.710
7.706
7.278
7.260
7.093
7.076
6.802
6.797
6.721
6.709
6.704
6.699
6.692
6.687
6.624
6.601
3.835
7.75 7.70 7.65 7.30 7.25 7.10 7.05 6.80 6.75 6.70 6.65 6.60 6.55
4.05
1.70
2.14
2.00
3.89
2.15
5.97
9.0 8.5 8.0 7.5 7.0 6.5 6.0 5.5 5.0 4.5 4.0 3.5 3.0 2.5 2.0 1.5

^{13}C NMR of compound (2b):

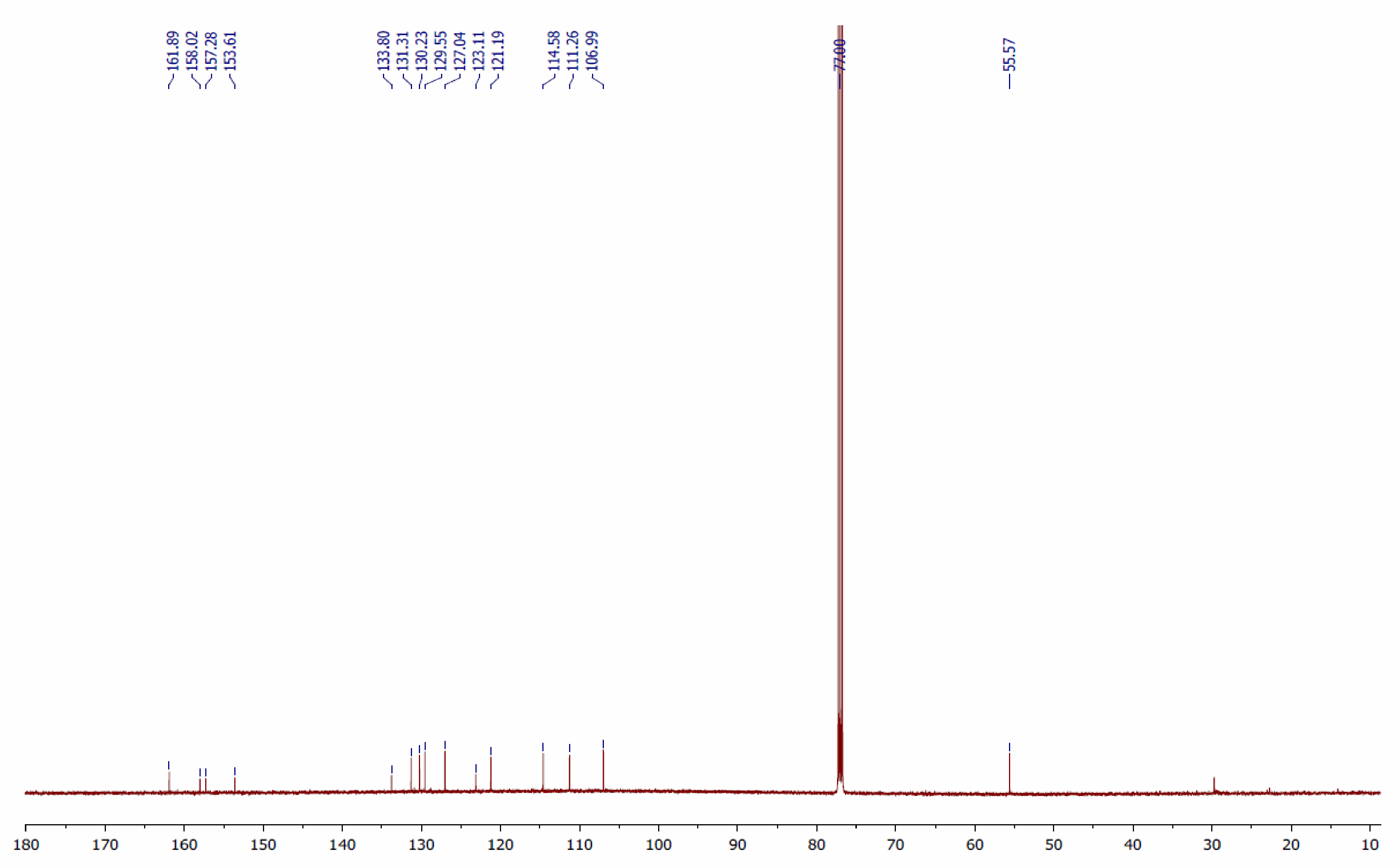

^{1}H NMR of compound (2c):

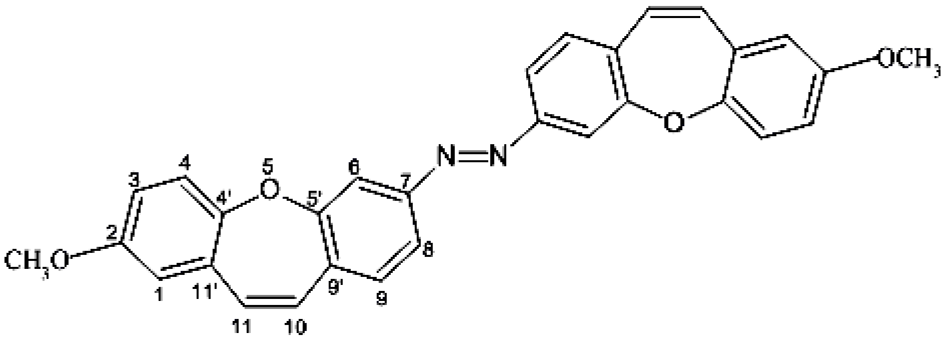

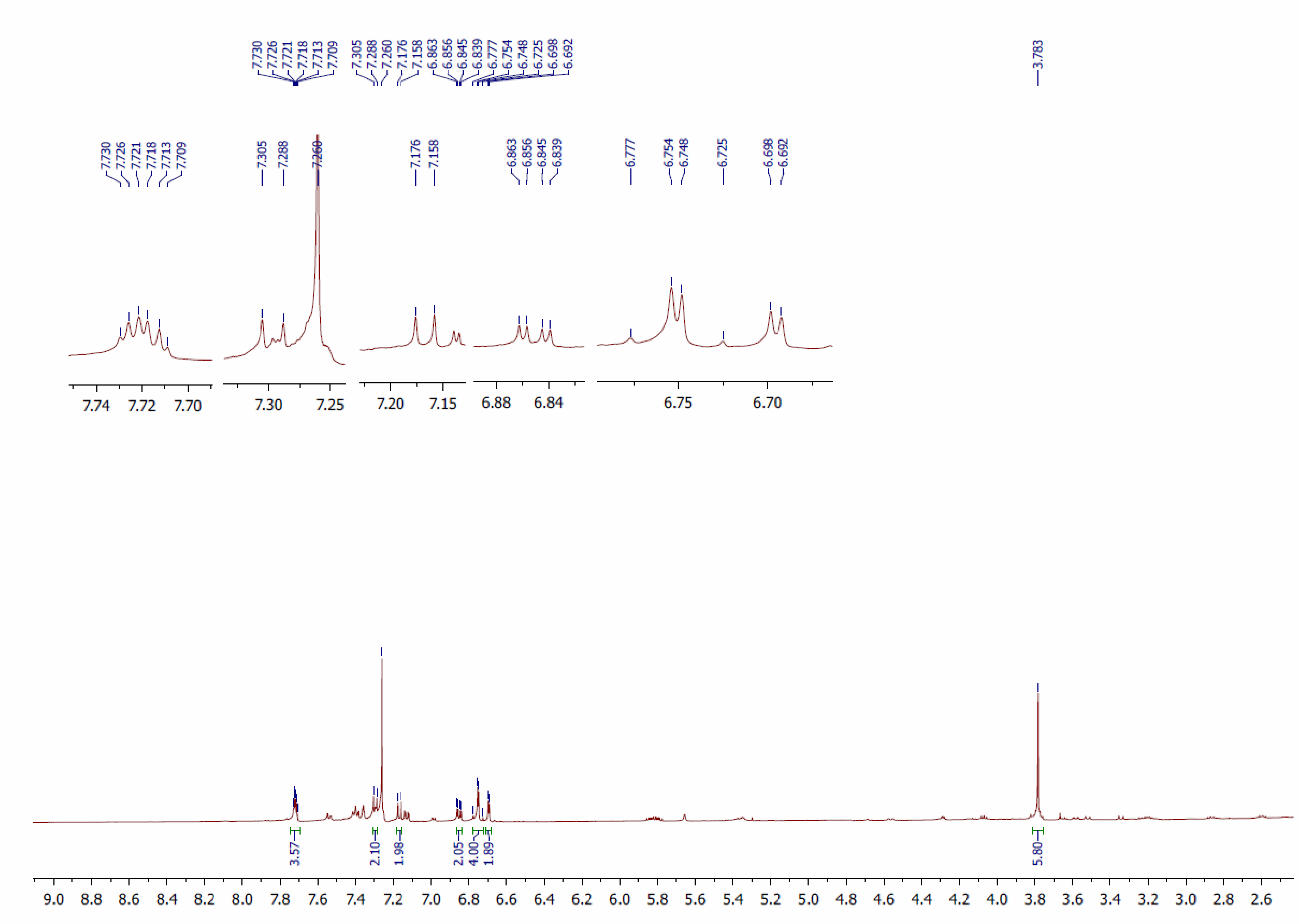
7.730
7.726
7.721
7.718
7.713
7.709
7.305
7.288
7.260
7.176
7.158
6.863
6.856
6.845
6.839
6.777
6.754
6.748
6.725
6.698
6.692
3.783
3.57
2.10
1.98
2.05
4.00
1.89
5.80

^{13}C NMR of compound (2c):

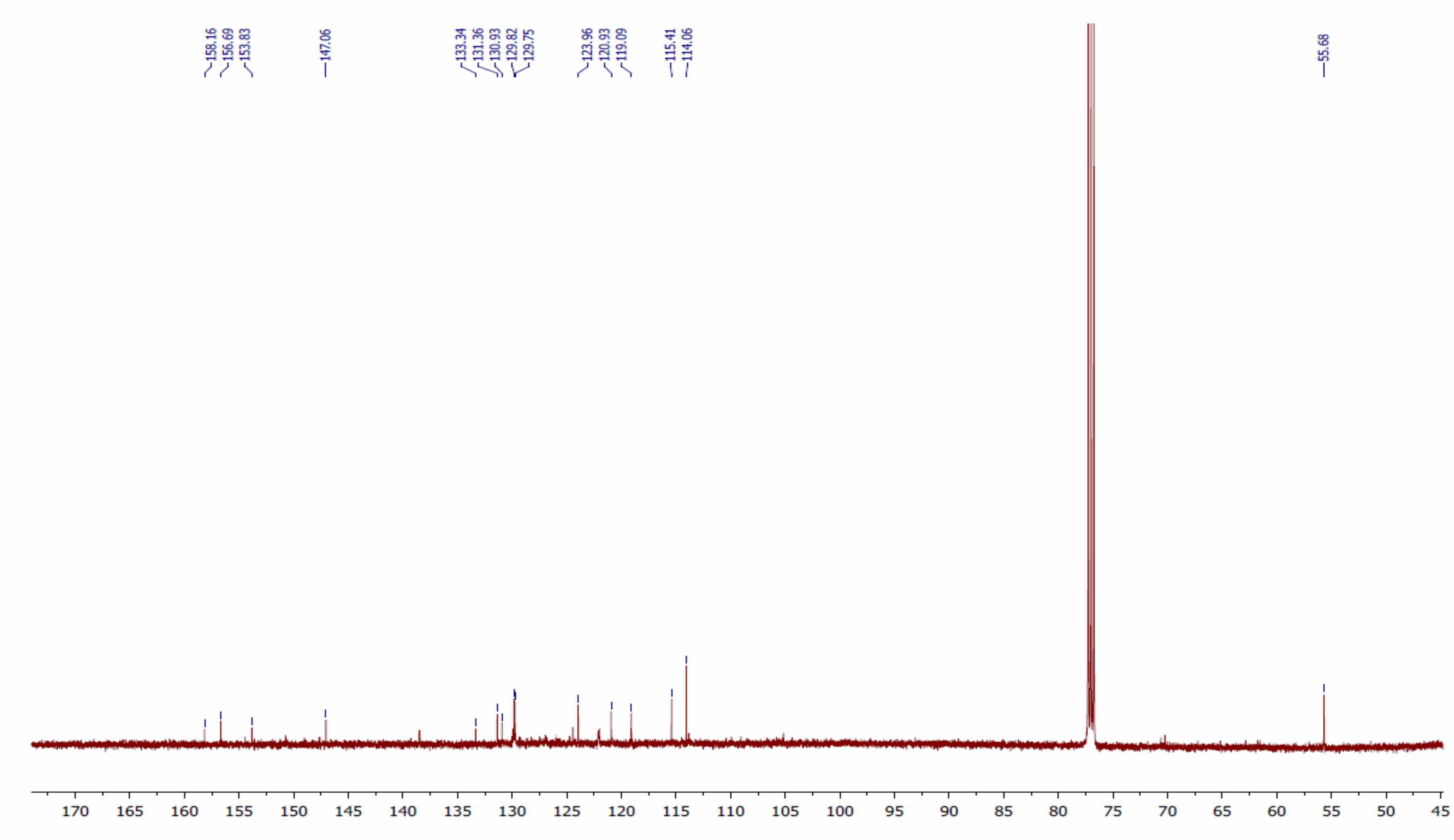

^{1}H NMR of compound (2d):

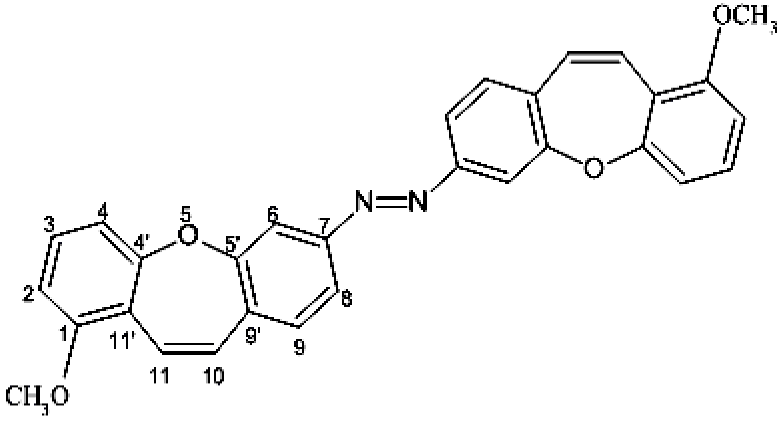

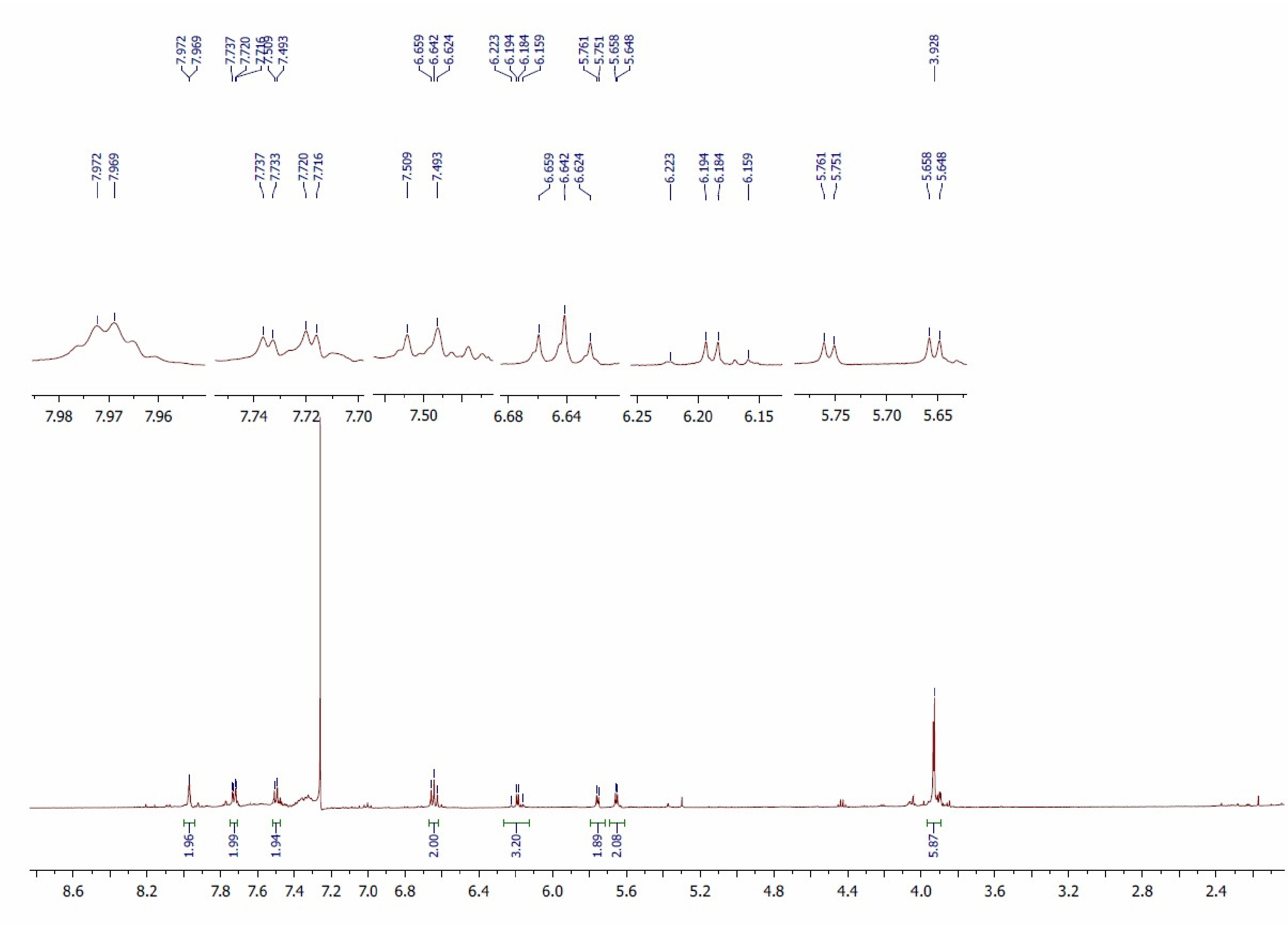
7.972
7.969
7.737
7.720
7.716
7.509
7.493
6.659
6.642
6.624
6.223
6.194
6.184
6.159
5.761
5.751
5.658
5.648
3.928
1.96
1.99
1.94
2.00
3.20
1.89
2.08
5.87

^{13}C NMR of compound (2d):

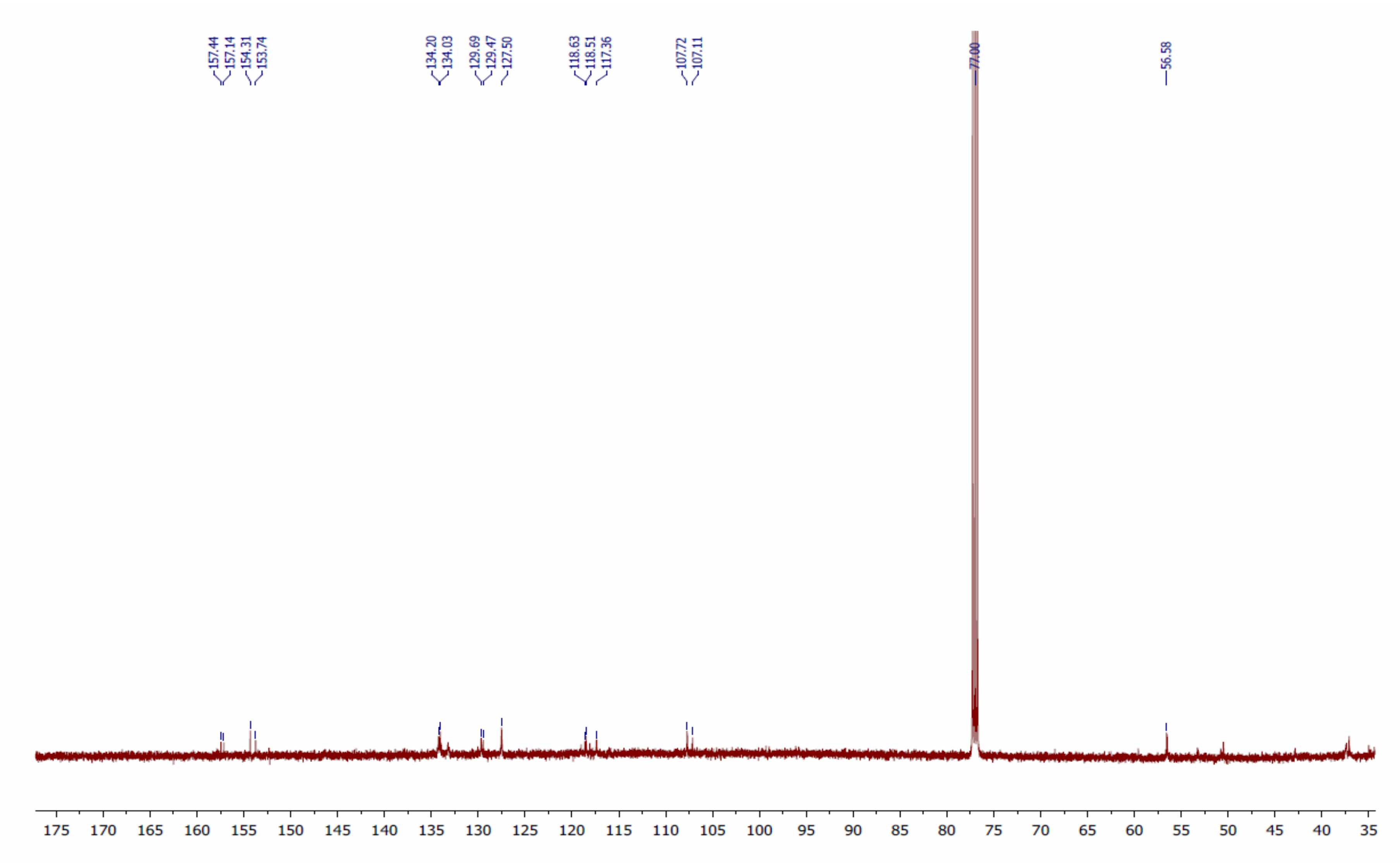

^{1}H NMR of compound (2e):

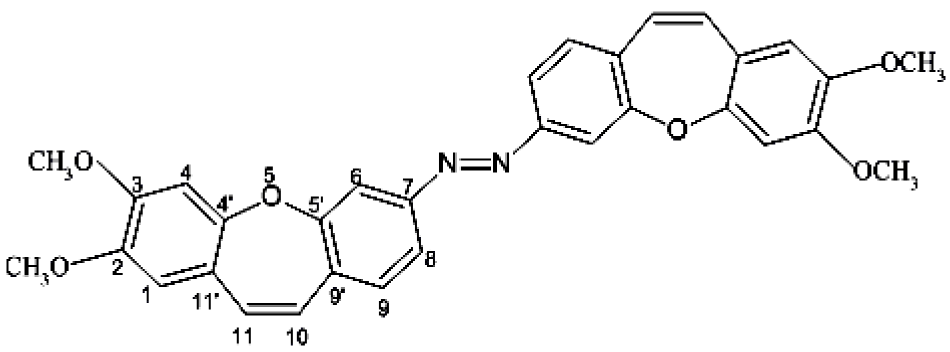

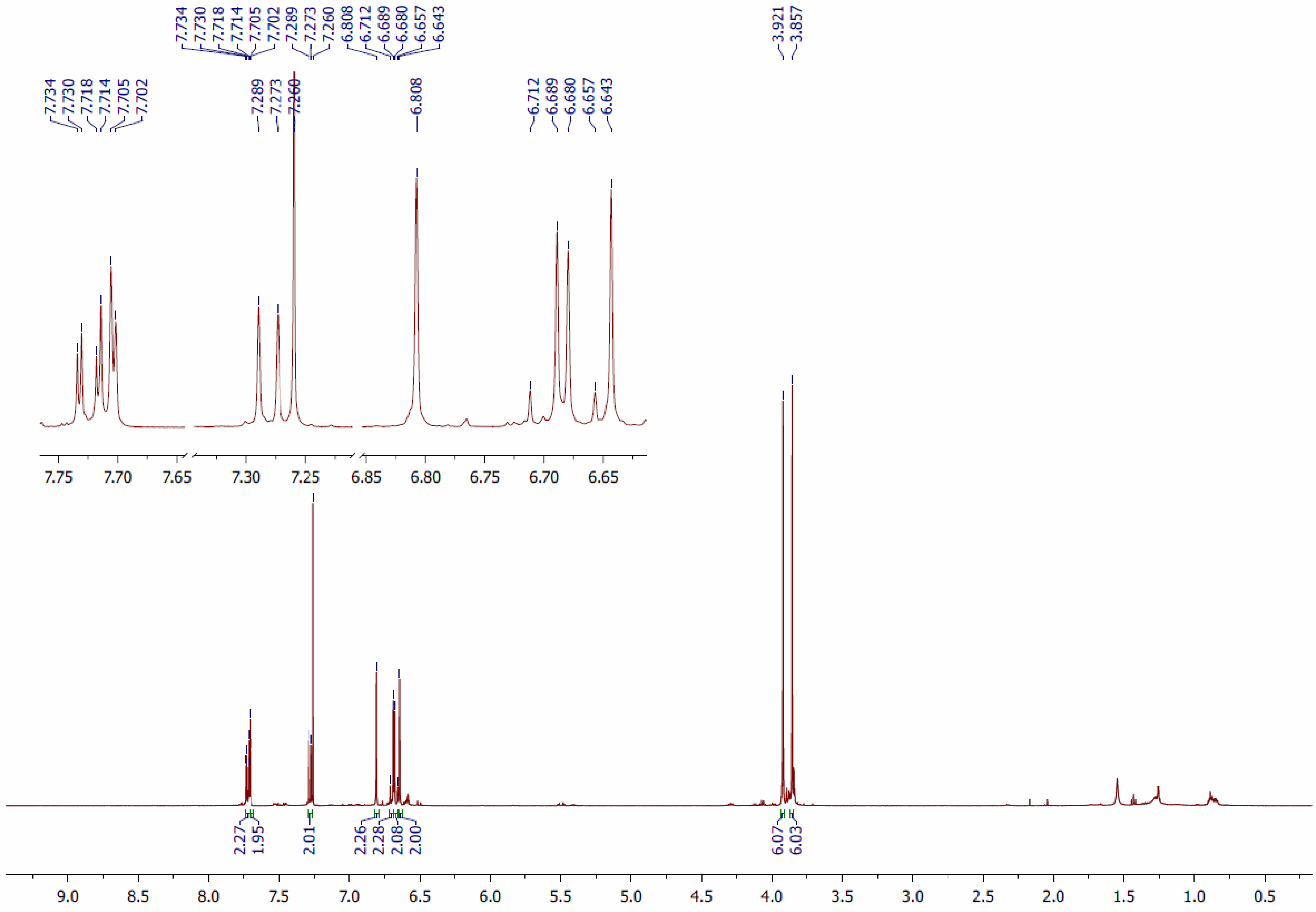
7.734
7.730
7.718
7.714
7.705
7.702
7.289
7.273
7.260
6.808
6.712
6.689
6.680
6.657
6.643
3.921
3.857
2.27
1.95
2.01
2.26
2.28
2.08
2.00
6.07
6.03
9.0 8.5 8.0 7.5 7.0 6.5 6.0 5.5 5.0 4.5 4.0 3.5 3.0 2.5 2.0 1.5 1.0 0.5
7.75 7.70 7.65 7.30 7.25 6.85 6.80 6.75 6.70 6.65

^{13}C NMR of compound (2e):

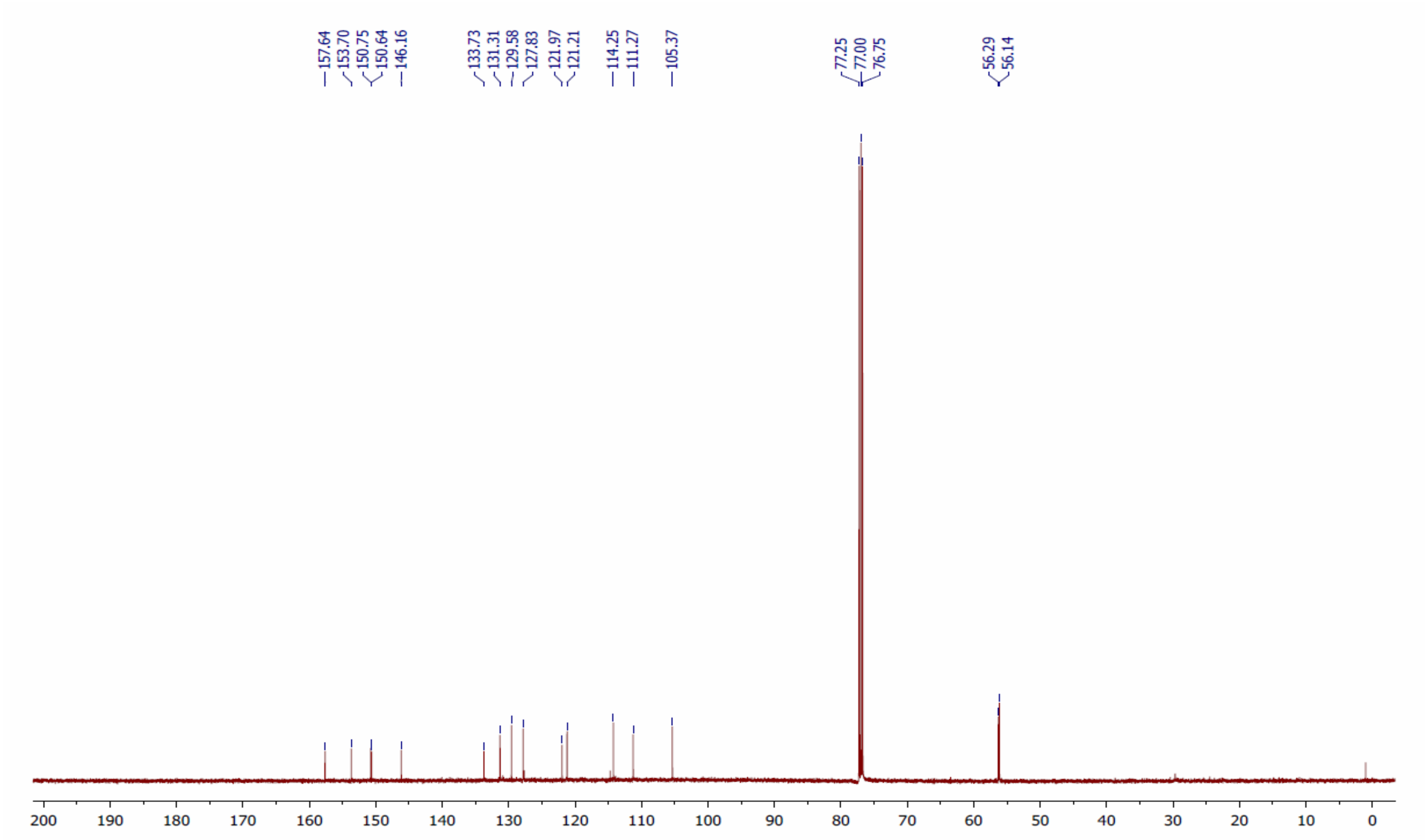

^{1}H NMR of compound (2f):

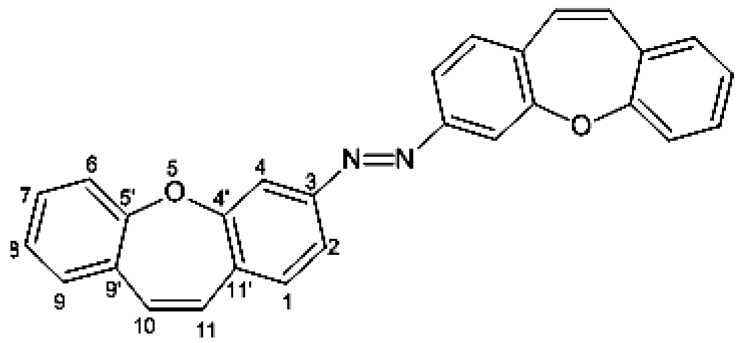

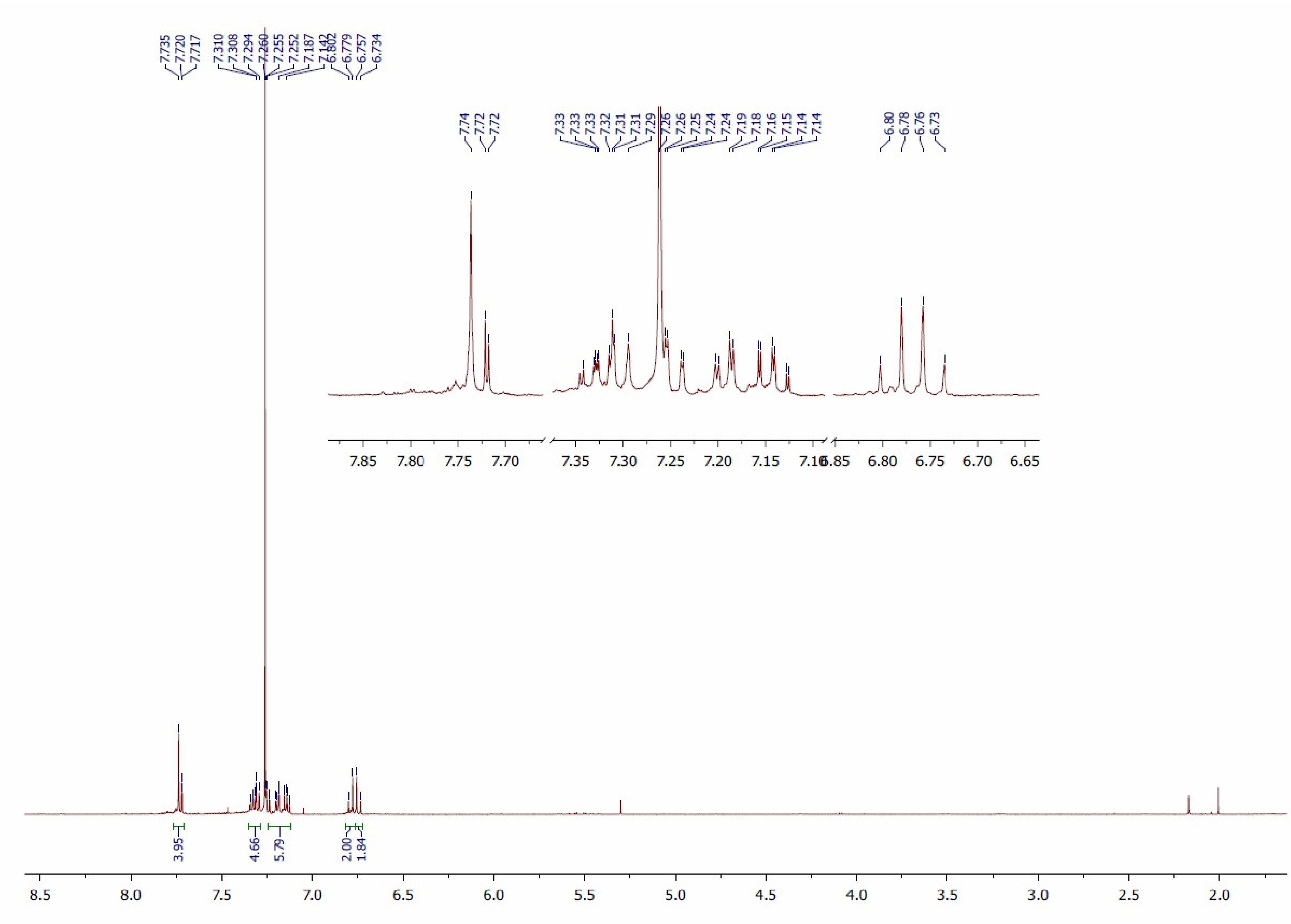
7.735
7.720
7.717
7.310
7.308
7.294
7.260
7.255
7.252
7.187
7.142
6.802
6.779
6.757
6.734
3.95
4.66
5.79
2.00
1.84
8.5 8.0 7.5 7.0 6.5 6.0 5.5 5.0 4.5 4.0 3.5 3.0 2.5 2.0

^{13}C NMR of compound (2f):

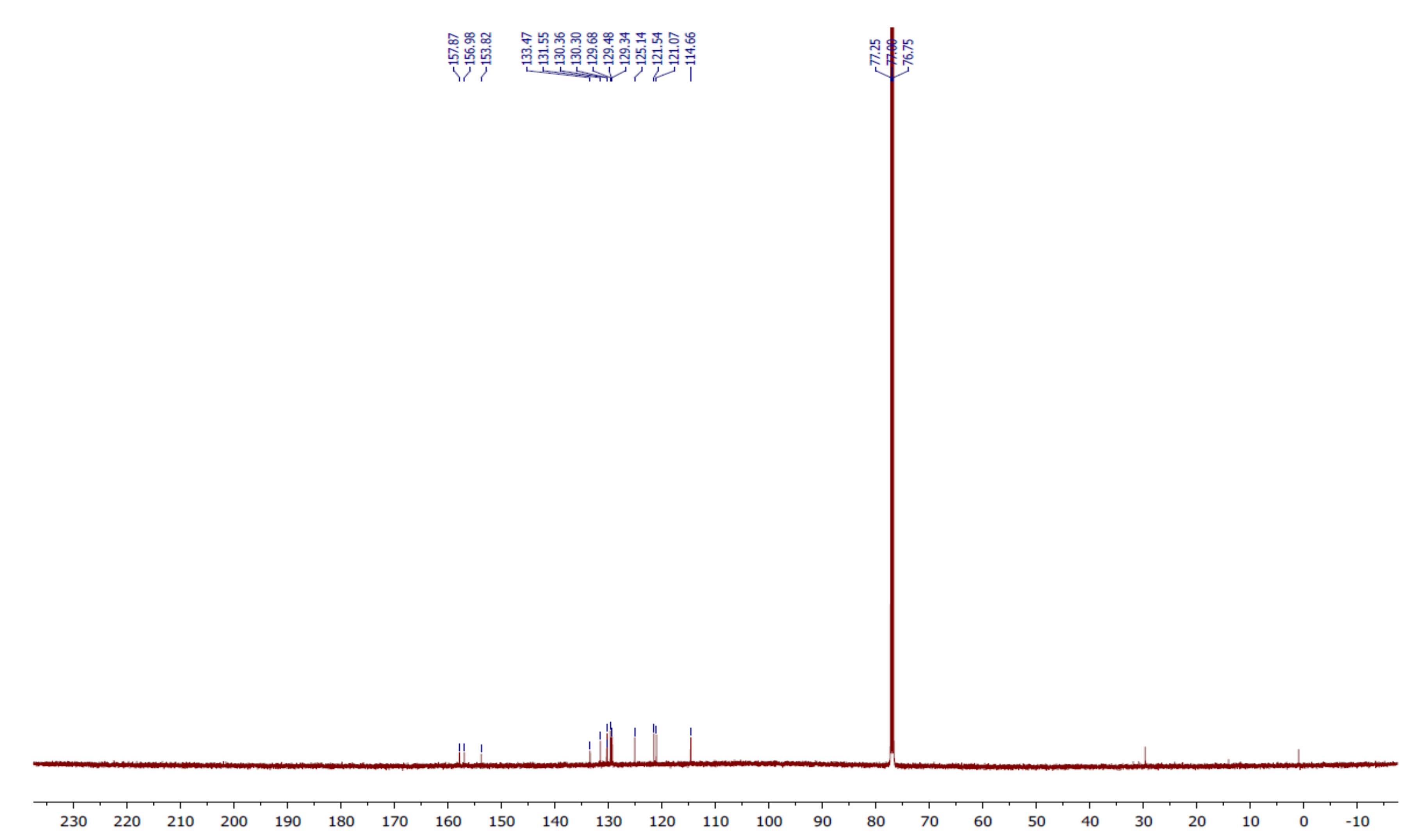

^{1}H NMR of compound (2g)

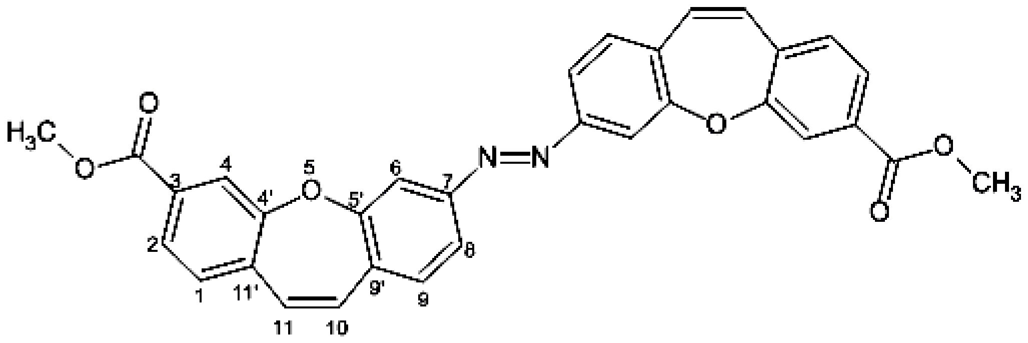

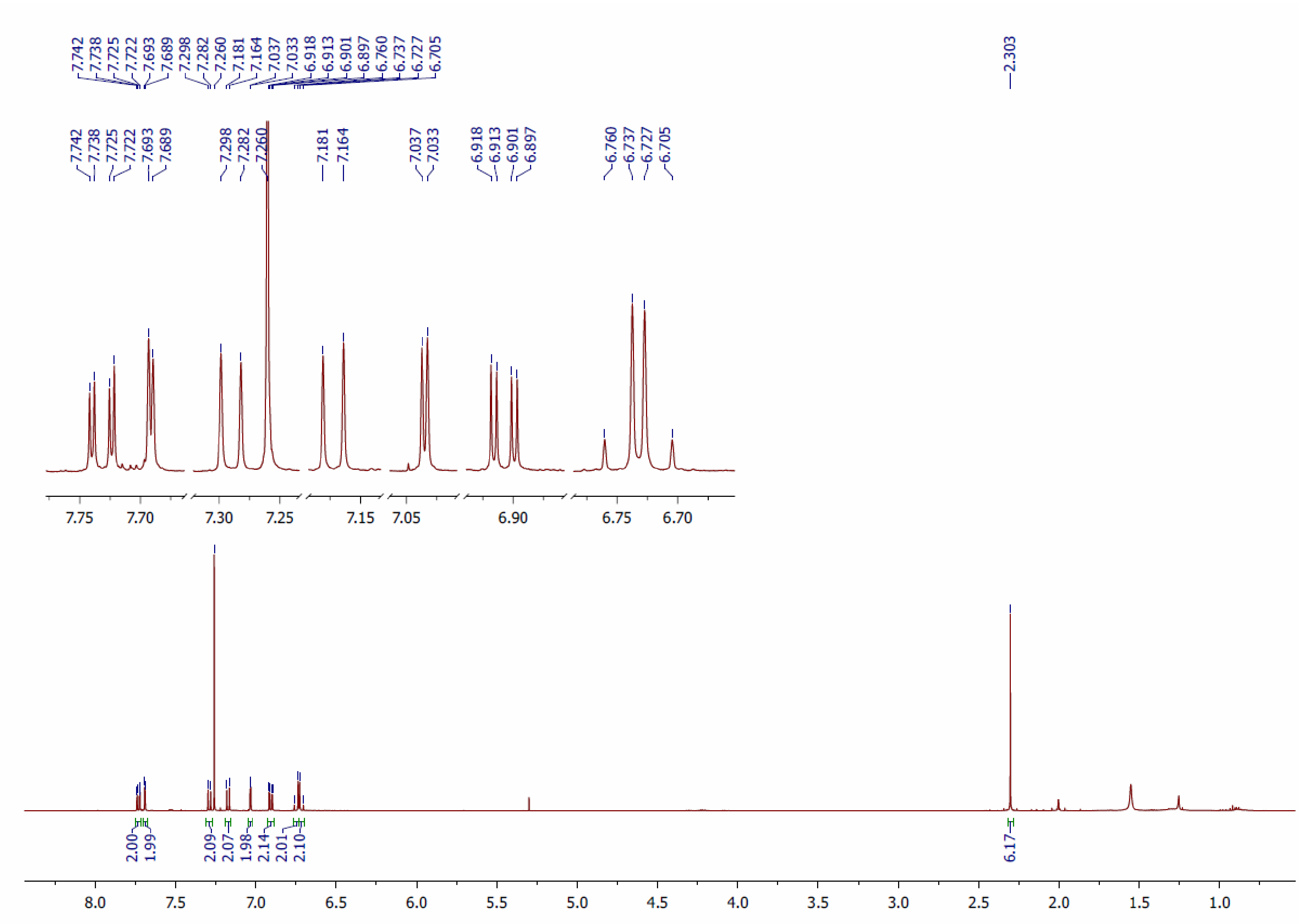
7.742
7.738
7.725
7.722
7.693
7.689
7.298
7.282
7.260
7.181
7.164
7.037
7.033
6.918
6.913
6.901
6.897
6.760
6.737
6.727
6.705
2.303
2.00
1.99
2.09
2.07
1.98
2.14
2.01
2.10
6.17

^{13}C NMR of compound (2g)

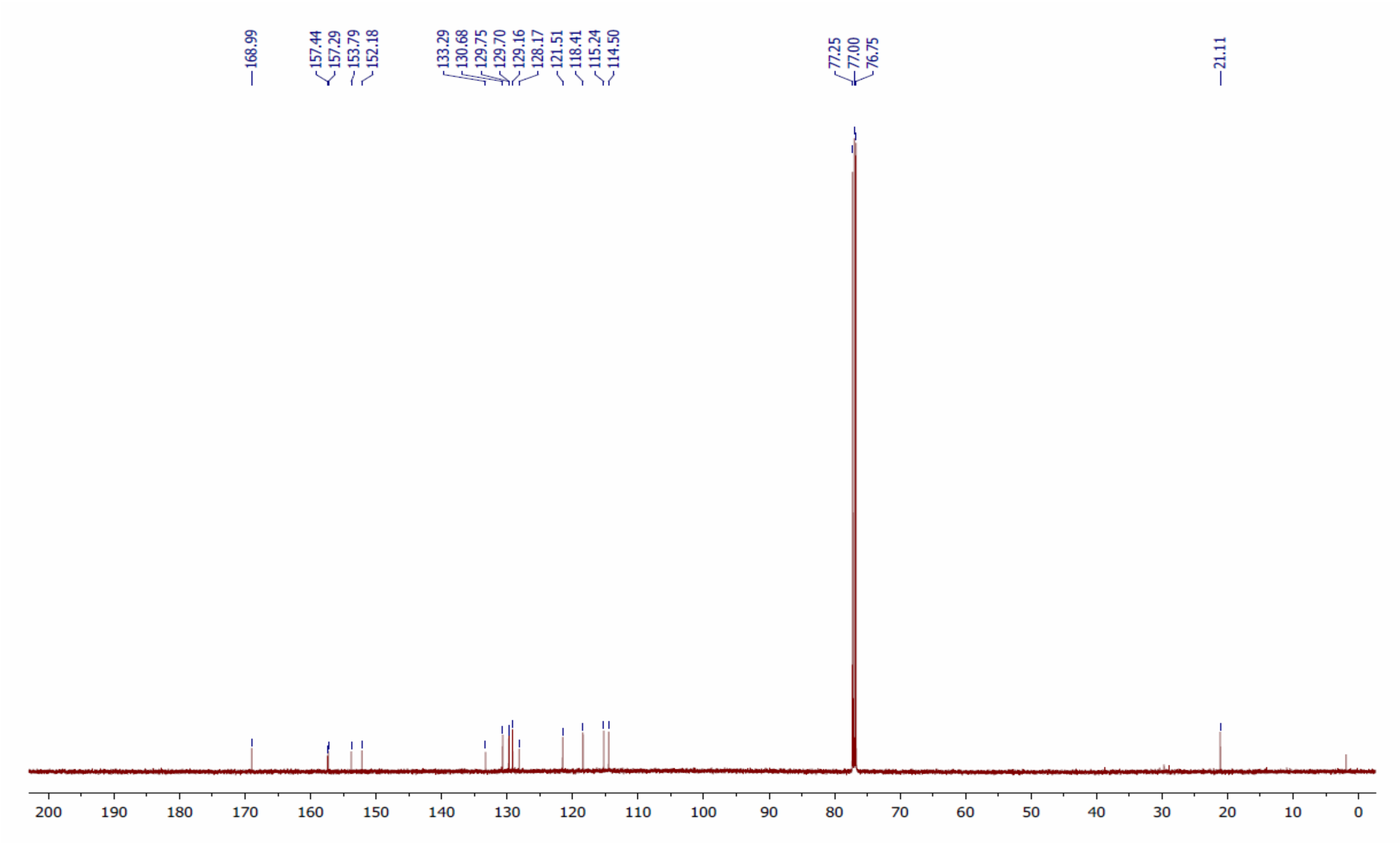

^{1}H NMR of compound (2h):

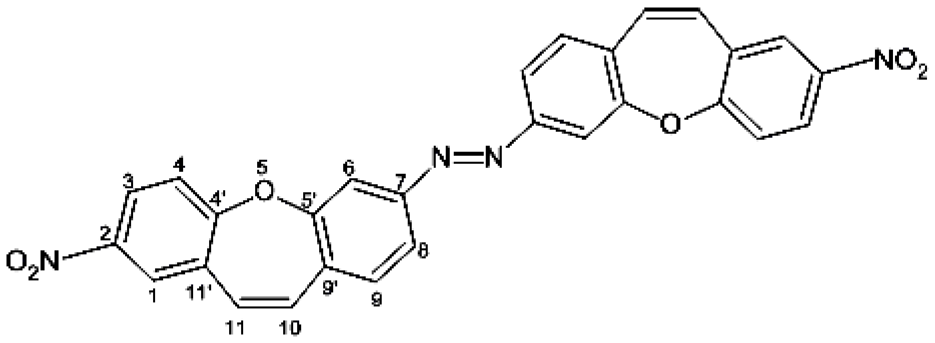

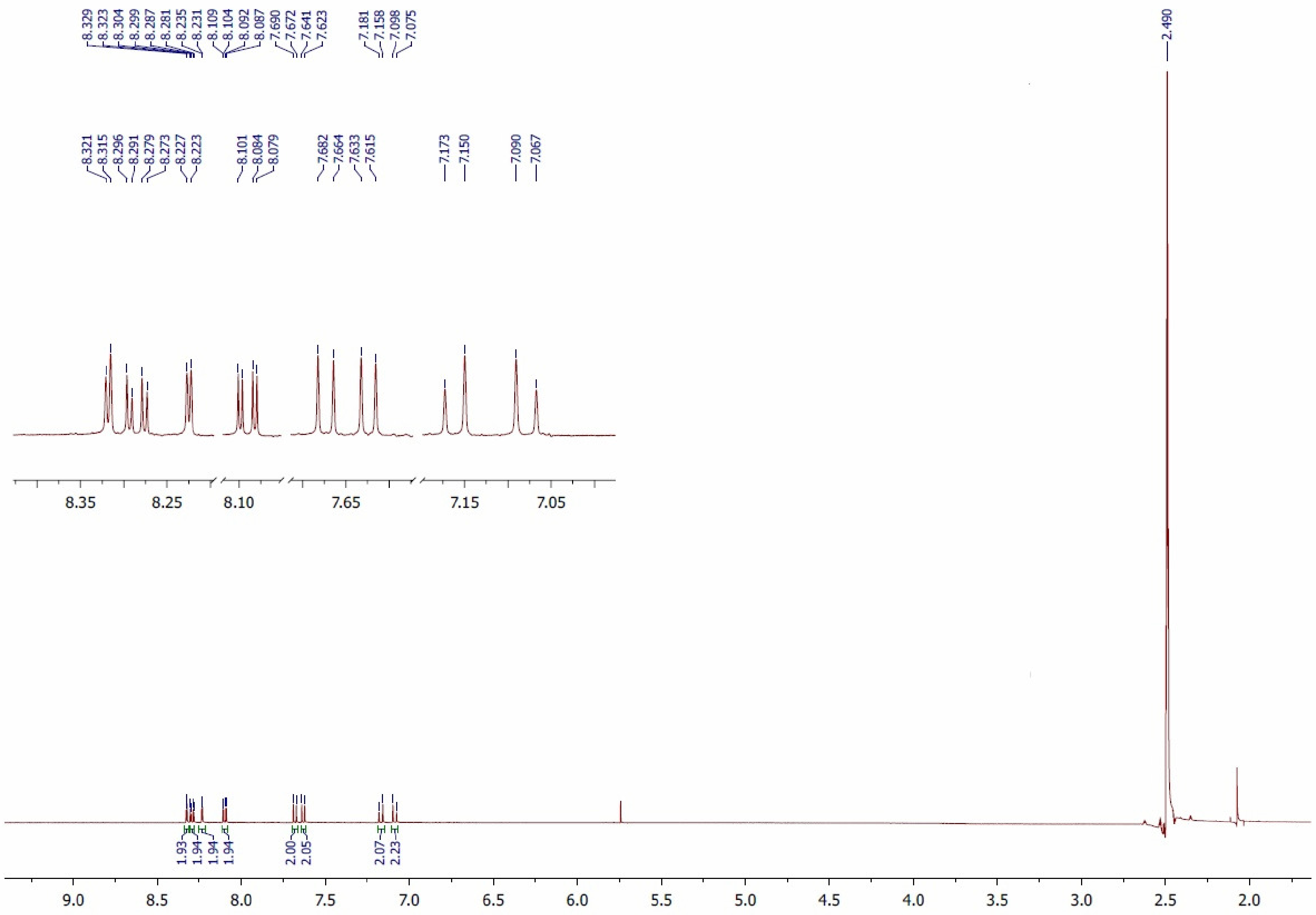
8.329
8.323
8.304
8.299
8.287
8.281
8.235
8.231
8.109
8.104
8.092
8.087
7.690
7.672
7.641
7.623
7.181
7.158
7.098
7.075
2.490
8.321
8.315
8.296
8.291
8.279
8.273
8.227
8.223
8.101
8.084
8.079
7.682
7.664
7.633
7.615
7.173
7.150
7.090
7.067
8.35
8.25
8.10
7.65
7.15
7.05
1.93
1.94
1.94
1.94
2.00
2.05
2.07
2.23
9.0
8.5
8.0
7.5
7.0
6.5
6.0
5.5
5.0
4.5
4.0
3.5
3.0
2.5
2.0

^{13}C NMR of compound (2h):

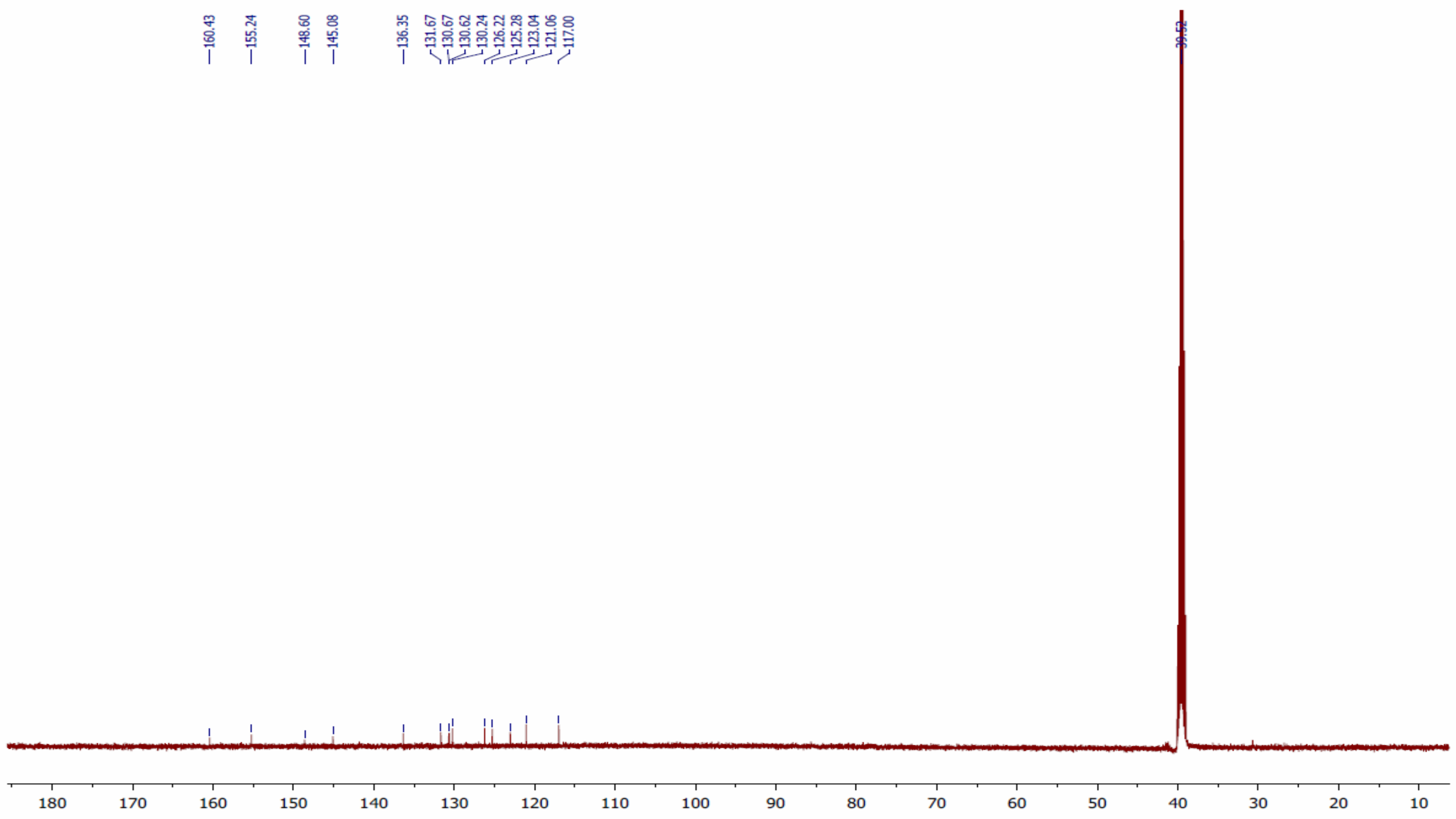

Copies of ^{1}H NMR and ^{13}C NMR spectra of (4c-e)

4a:

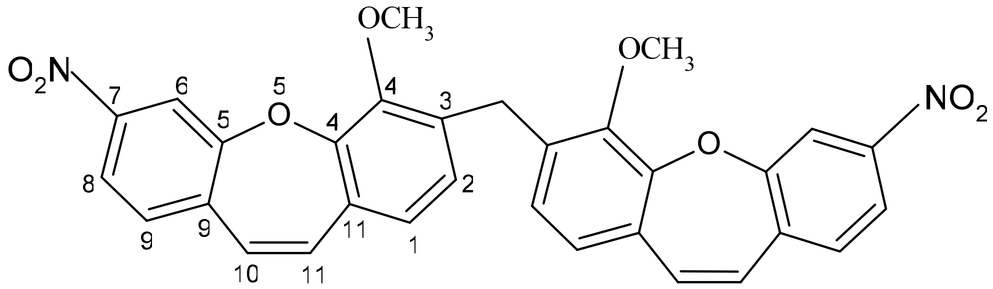

Characterization and spectra can be found in P. Tobiasz, M. Poterała, E. Jaśkowska, H. Krawczyk, *RSC Adv.*, 2018, 8, 30678-30682

^{1}H NMR of compound (4c4):

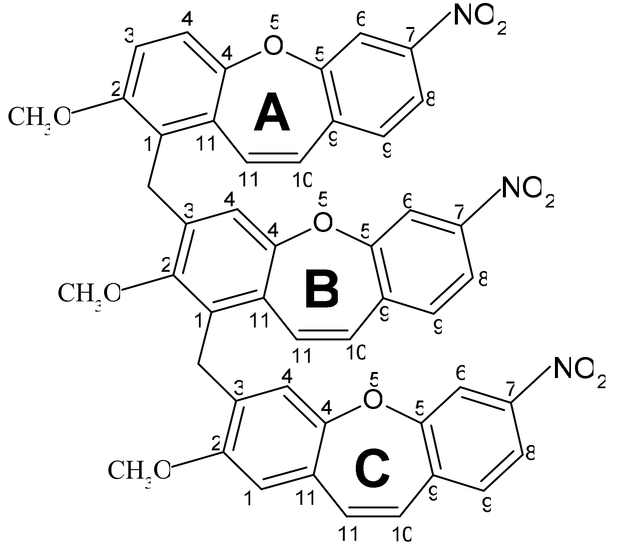

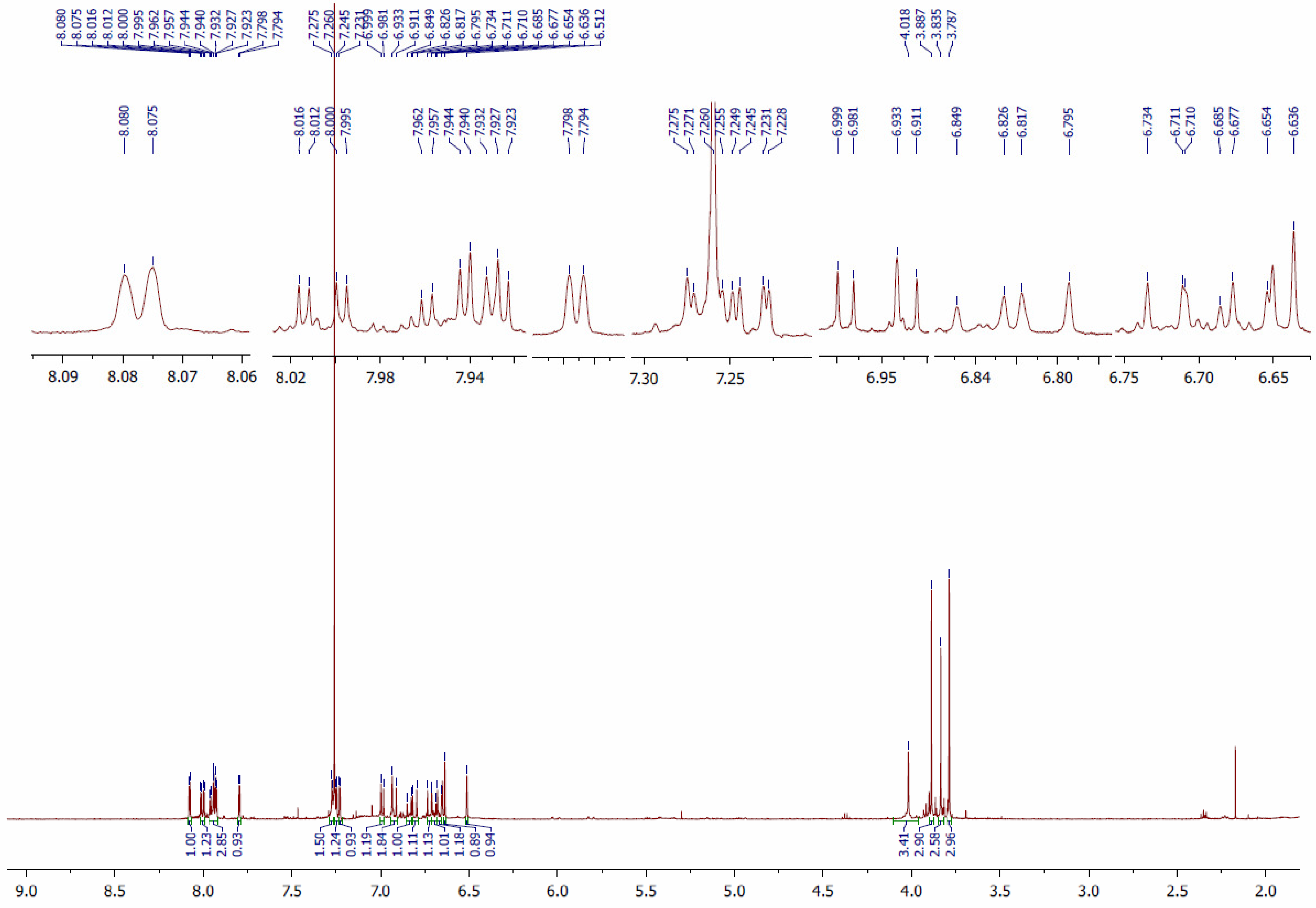

^{13}C NMR of compound (4c4):

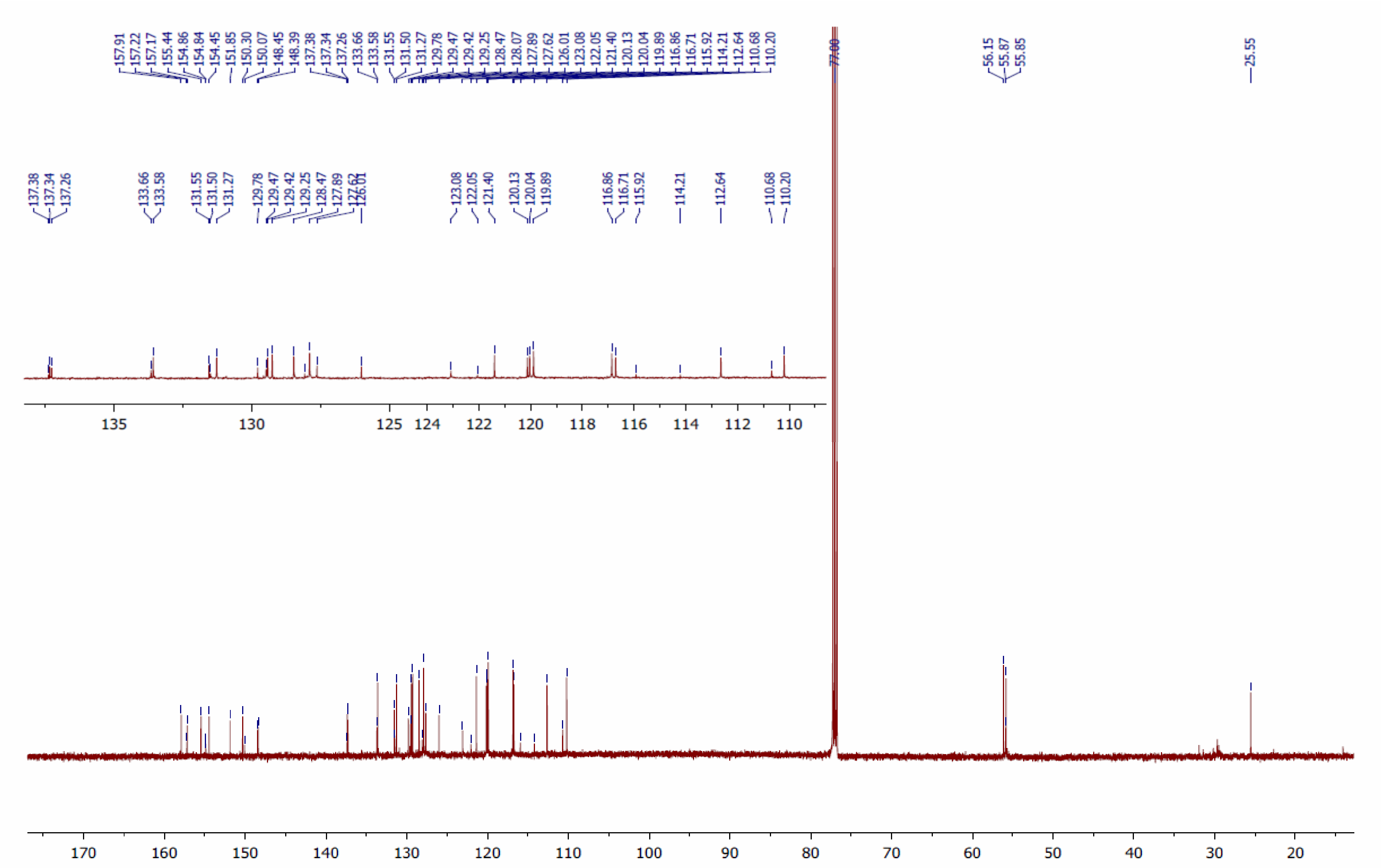

¹H NMR of compound (4d):

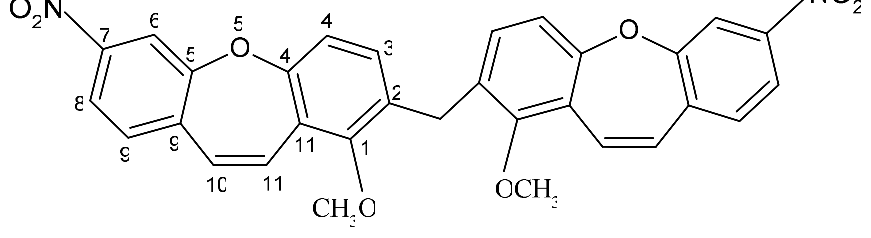

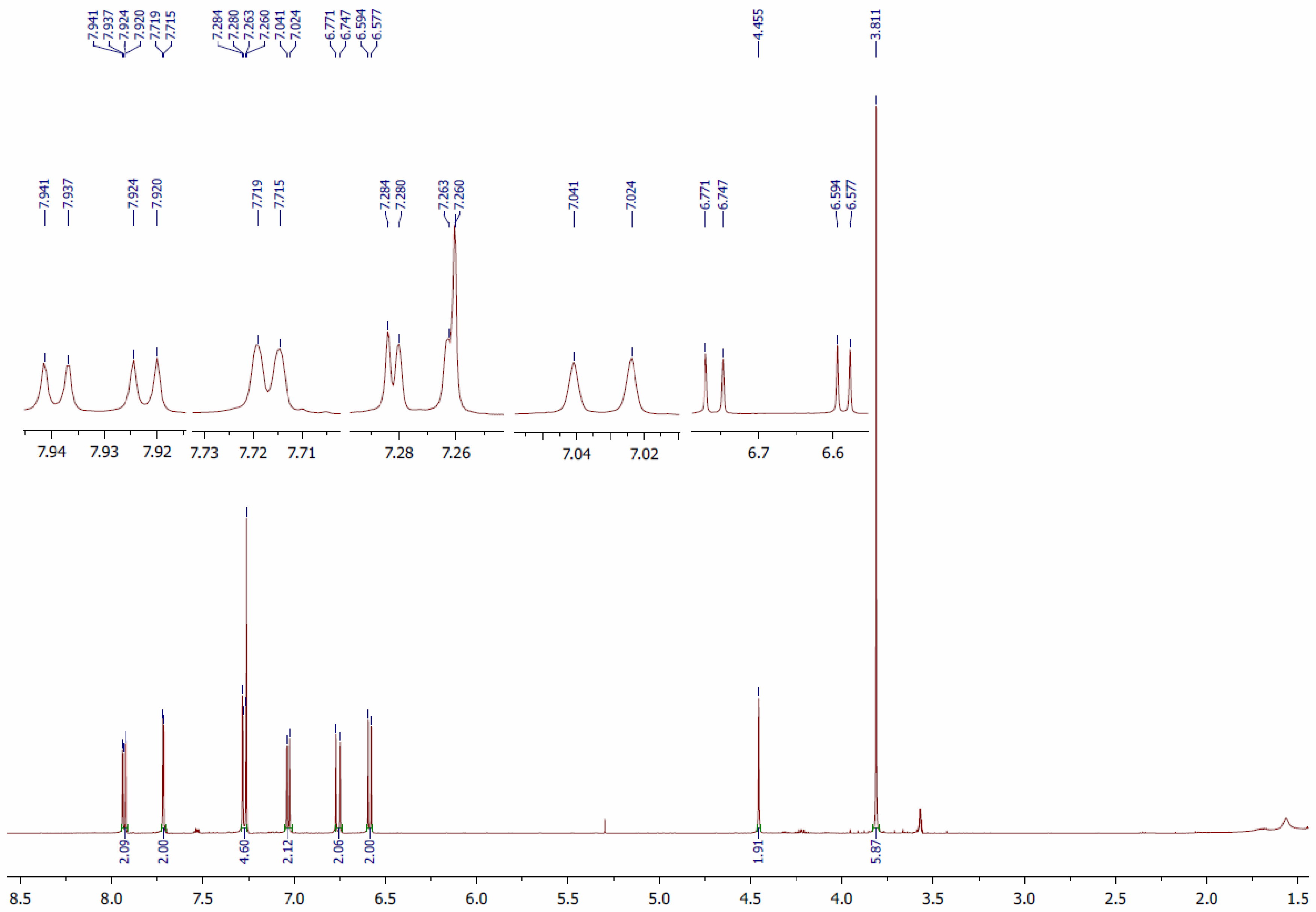
7.941
7.937
7.924
7.920
7.719
7.715
7.284
7.280
7.263
7.260
7.041
7.024
6.771
6.747
6.594
6.577
4.455
3.811
2.09
2.00
4.60
2.12
2.06
2.00
1.91
5.87
8.5
8.0
7.5
7.0
6.5
6.0
5.5
5.0
4.5
4.0
3.5
3.0
2.5
2.0
1.5

^{13}C NMR of compound (4d):

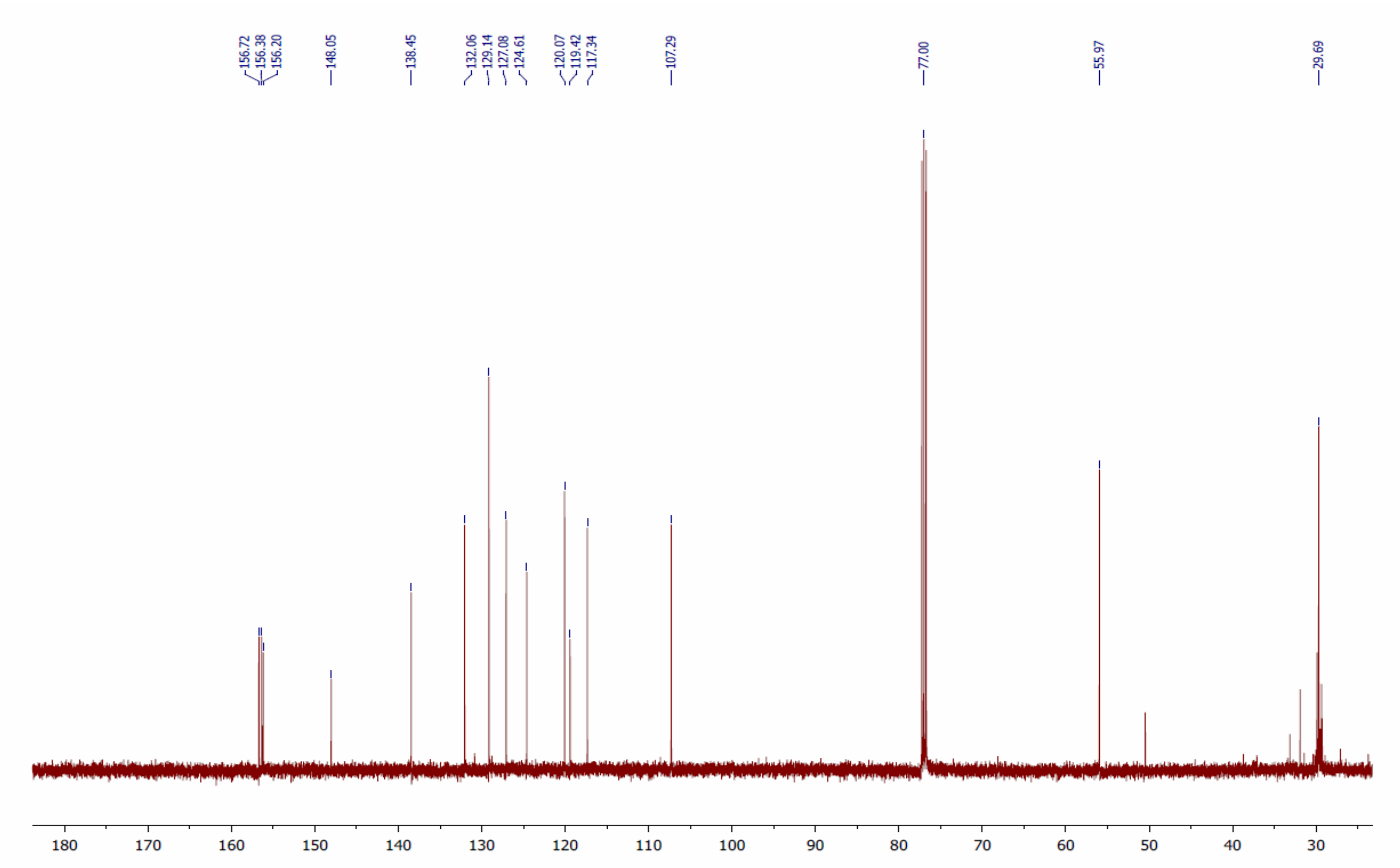

^{1}H NMR of (4e) mixture:

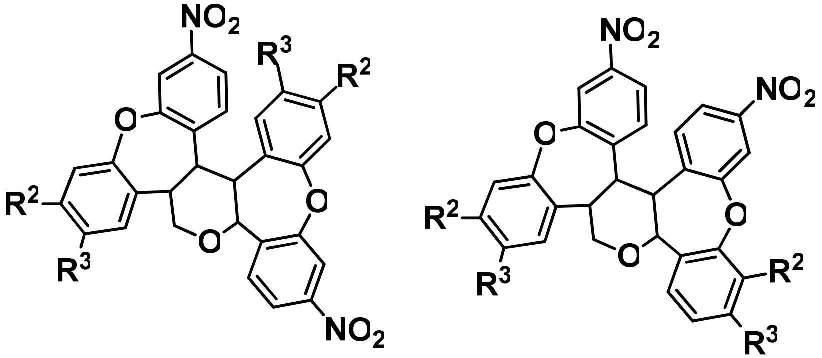

4e

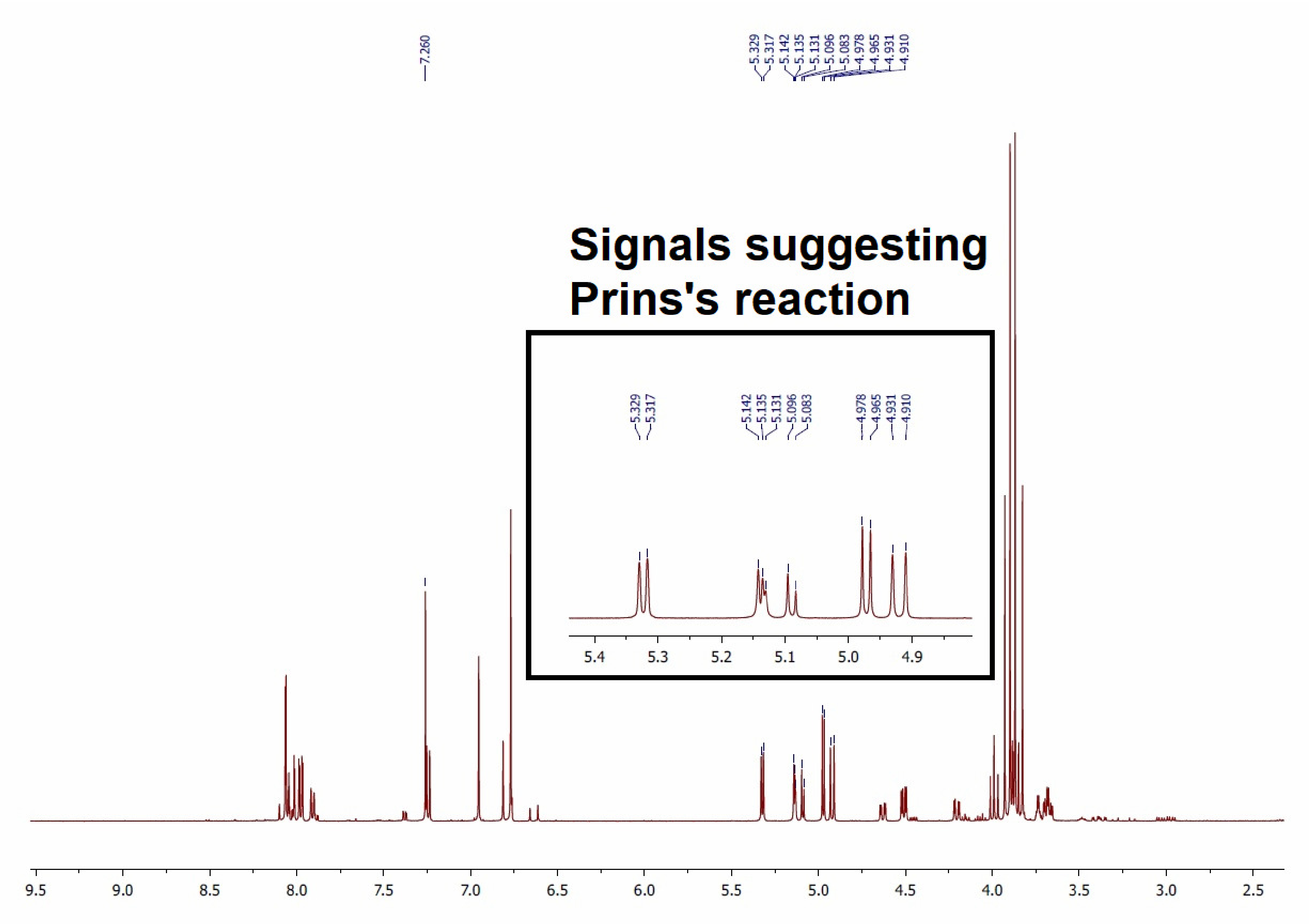
7.260
5.329
5.317
5.142
5.135
5.131
5.096
5.083
4.978
4.965
4.931
4.910
Signals suggesting Prins's reaction
5.4
5.3
5.2
5.1
5.0
4.9
9.5
9.0
8.5
8.0
7.5
7.0
6.5
6.0
5.5
5.0
4.5
4.0
3.5
3.0
2.5

Copies of ^{1}H NMR and ^{13}C NMR spectra of (5a-f)

5a:

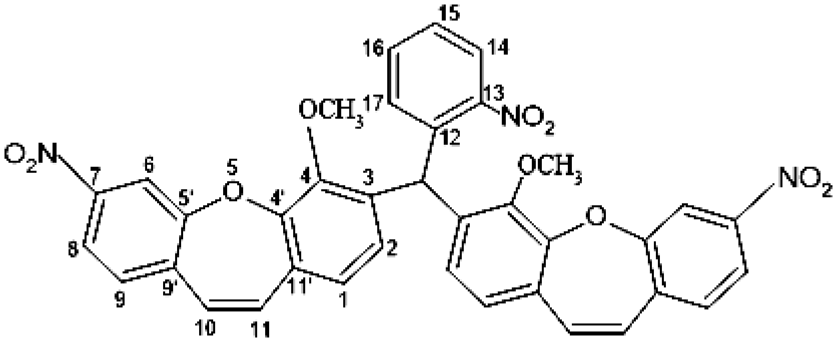

^{1}H NMR of compound (5a): (500 MHz, CDCl3, 298 K): δ (ppm): 8.19 (2H, d, $J_{H6,H8}$ = 2.5 Hz, H_6), 8.00 (1H, dd, $J_{H14,H15}$ = 8.5 Hz, $J_{H14,H16}$ = 2 Hz, H_{14}), 8.00 (2H, d, $J_{H8,H9}$ = 8.5 Hz, H_8), 7.34-7.32 (1H, m, H_{16}), 7.30 (2H, d, H_9), 7.11 (2H, d, $J_{H1,H2}$ = 8.5 Hz, H_1), 7.03 (2H, AB spin system, d, $J_{H10,H11}$ = 11.5 Hz, H_{11}), 6.93-6.83 (2H, m, H_{15}, H_{17}), 6.82 (2H, AB spin system, d, H_{10}), 6.67 (2H, d, H_2), 6.66 (1H, s, CH), 3.94 (6H, s, OCH_3).

^{13}C NMR of compound (5a): (125 MHz, CDCl3, 298 K): δ (ppm): 157.53 (C5'), 151.51 (C4), 148.56 (C13), 147.18 (C7), 146.10 (C4'), 145.21 (C12), 137.36 (C9'), 135.40 (C16), 133.20 (C17), 130.31 (C10),129.98 (C11) 128.84 (C3), 128.66 (C11'), 128.52 (C9), 126.56 (C15), 124.21 (C2), 120.11 (C14), 120.05 (C8), 117.45 (C6), 112.81 (C1), 56.14 (OCH_3), 40.78 (CH).

HRMS (ESI): *m/z* calculated for $C_{37}H_{25}N_3O_{10}$+H 672.16127, found: 672.16127.
Light yellow powder.

^{1}H NMR of compound (5b):

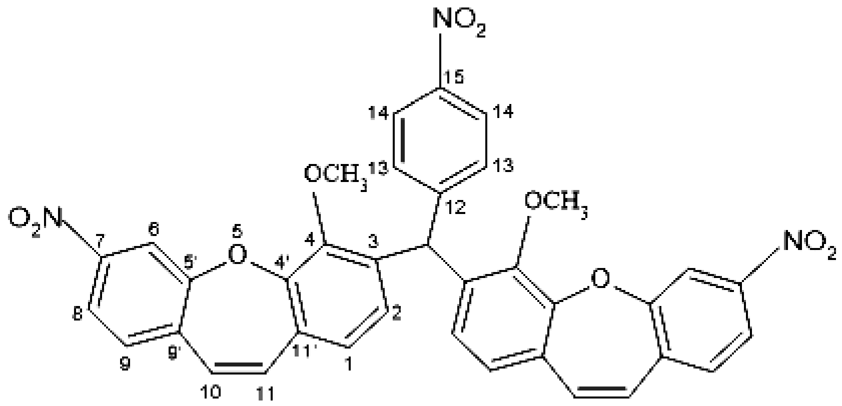

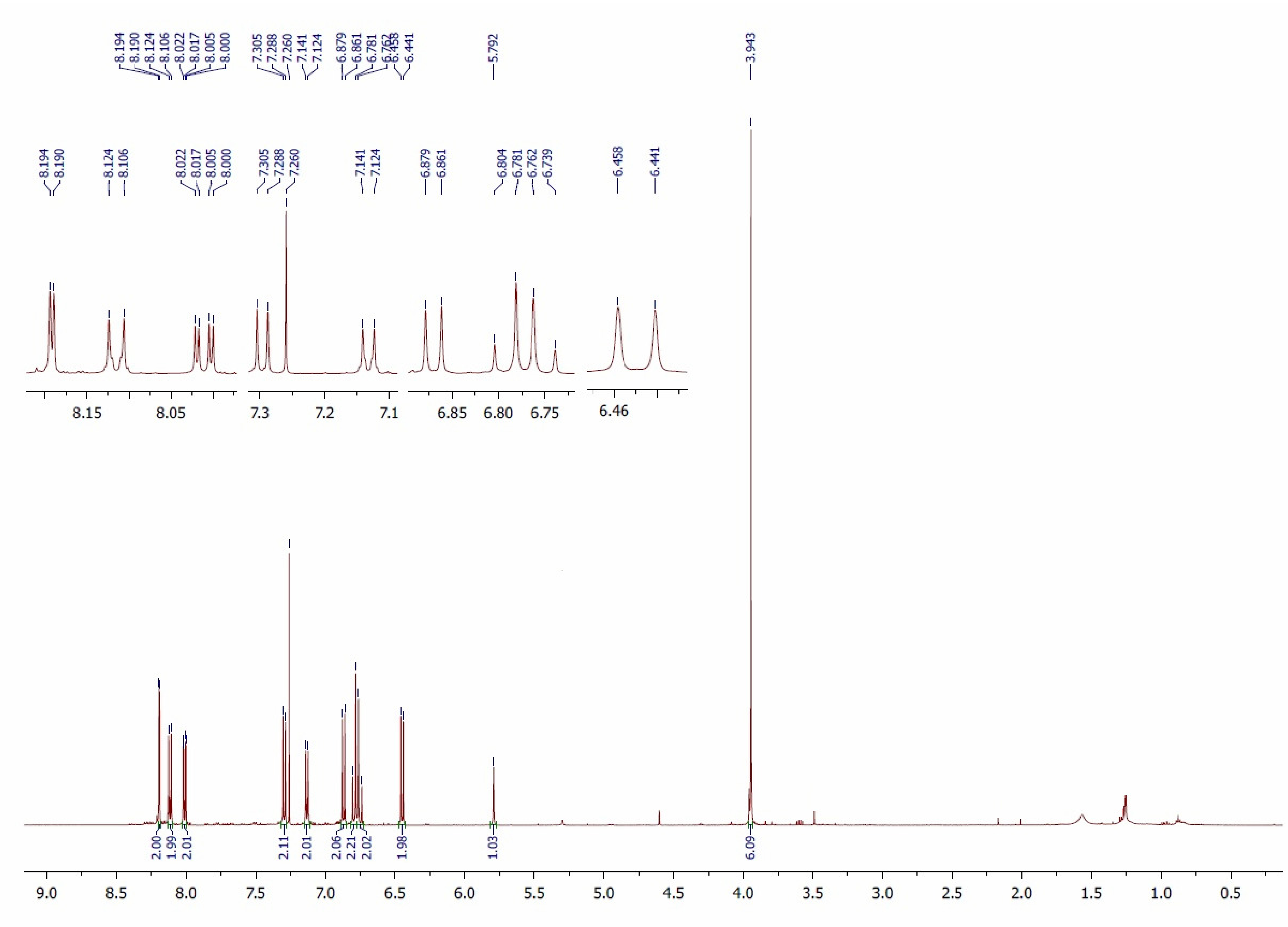
8.194
8.190
8.124
8.106
8.022
8.017
8.005
8.000
7.305
7.288
7.260
7.141
7.124
6.879
6.861
6.804
6.781
6.762
6.739
6.458
6.441
5.792
3.943
2.00
1.99
2.01
2.11
2.01
2.06
2.21
2.02
1.98
1.03
6.09

^{13}C NMR of compound (5b):

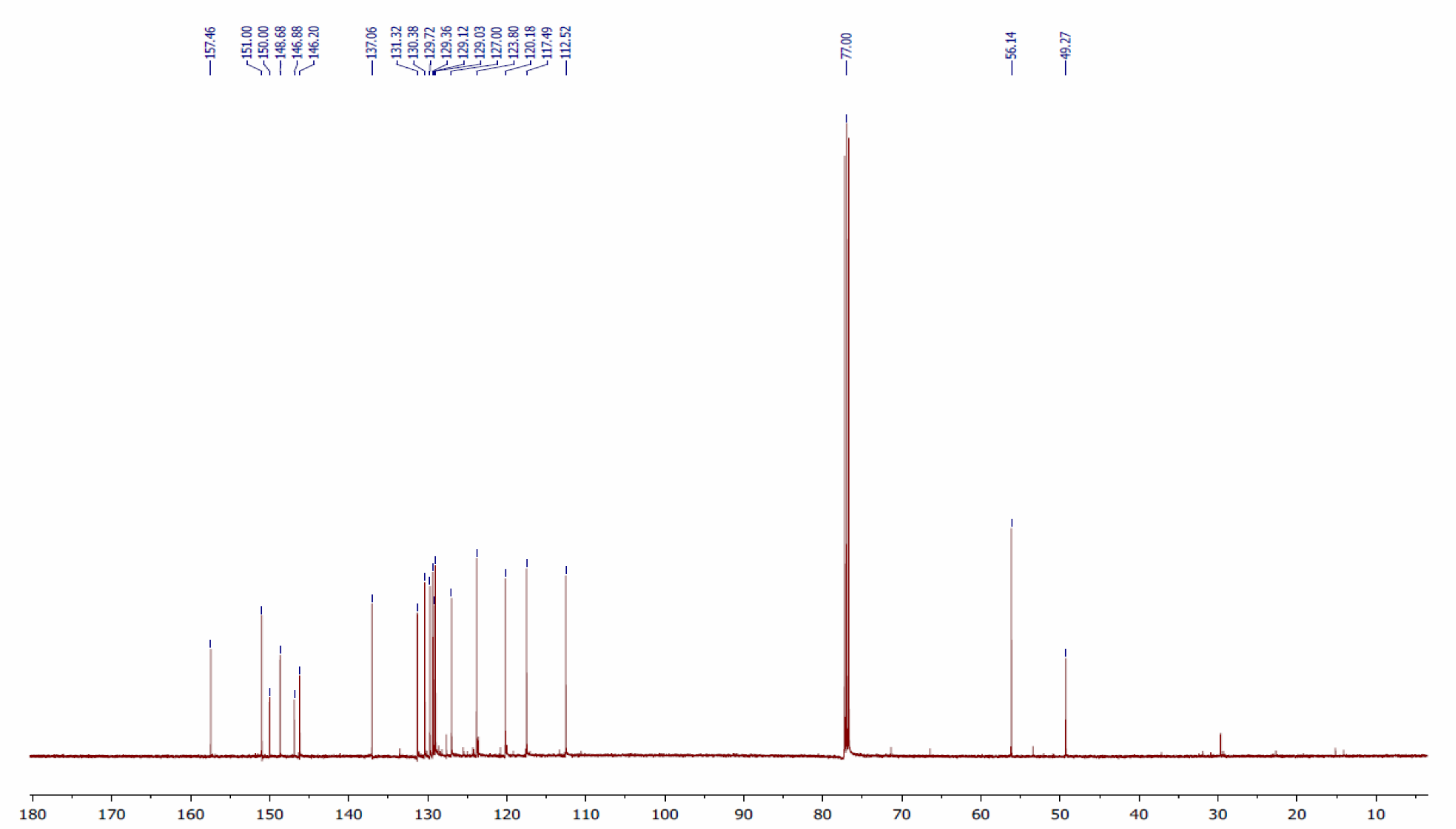

^{1}H NMR of compound (5c):

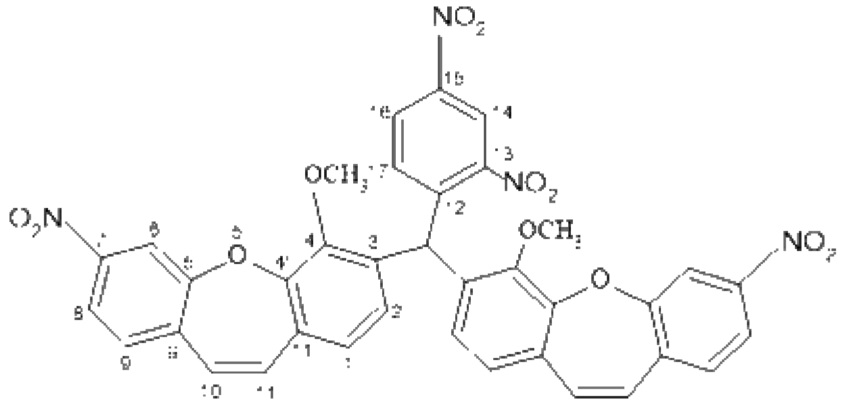

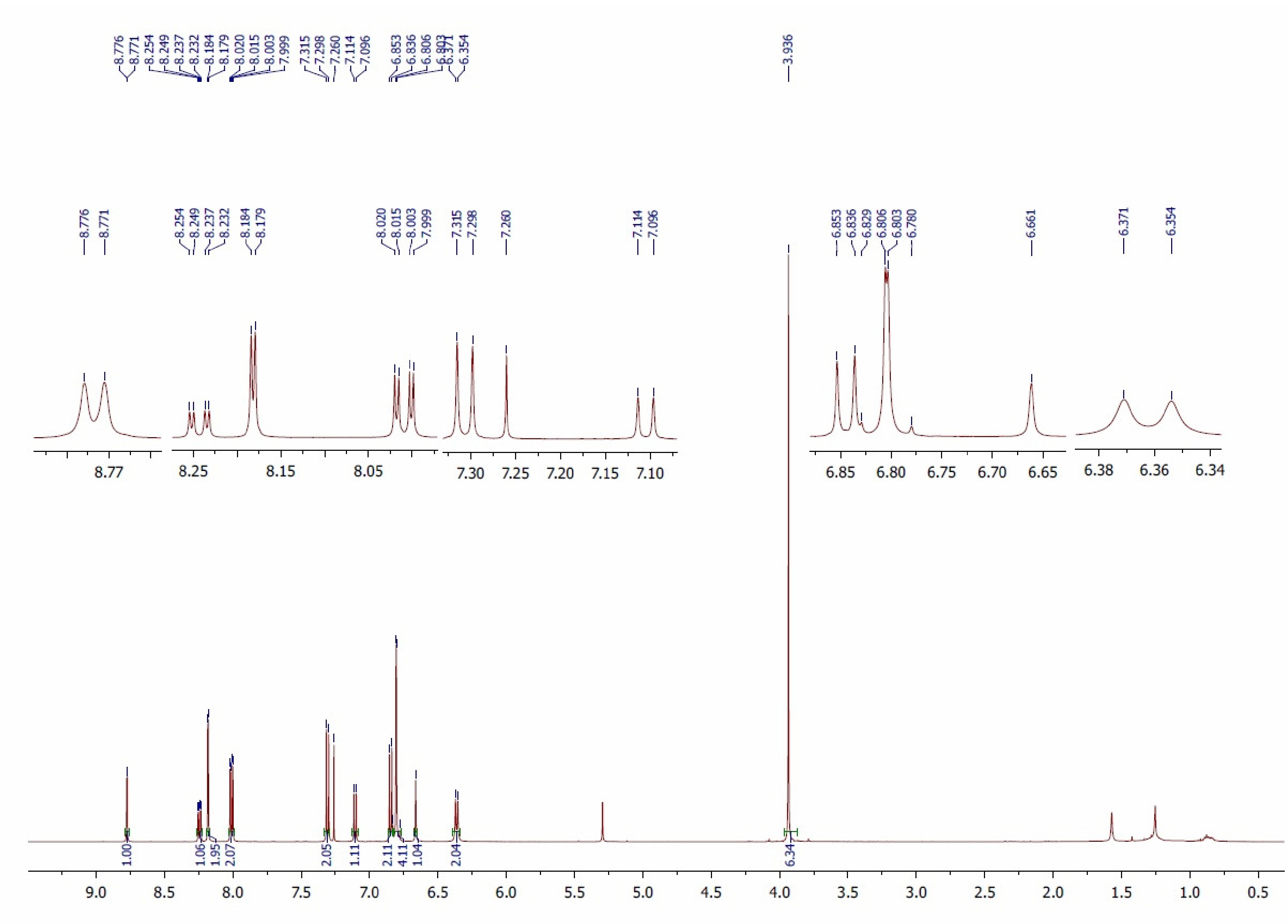
8.776
8.771
8.254
8.249
8.237
8.232
8.184
8.179
8.020
8.015
8.003
7.999
7.315
7.298
7.260
7.114
7.096
6.853
6.836
6.829
6.806
6.803
6.780
6.661
6.371
6.354
3.936
1.00
1.06
1.95
2.07
2.05
1.11
2.11
4.11
1.04
2.04
6.34
9.0 8.5 8.0 7.5 7.0 6.5 6.0 5.5 5.0 4.5 4.0 3.5 3.0 2.5 2.0 1.5 1.0 0.5

^{13}C NMR of compound (5c):

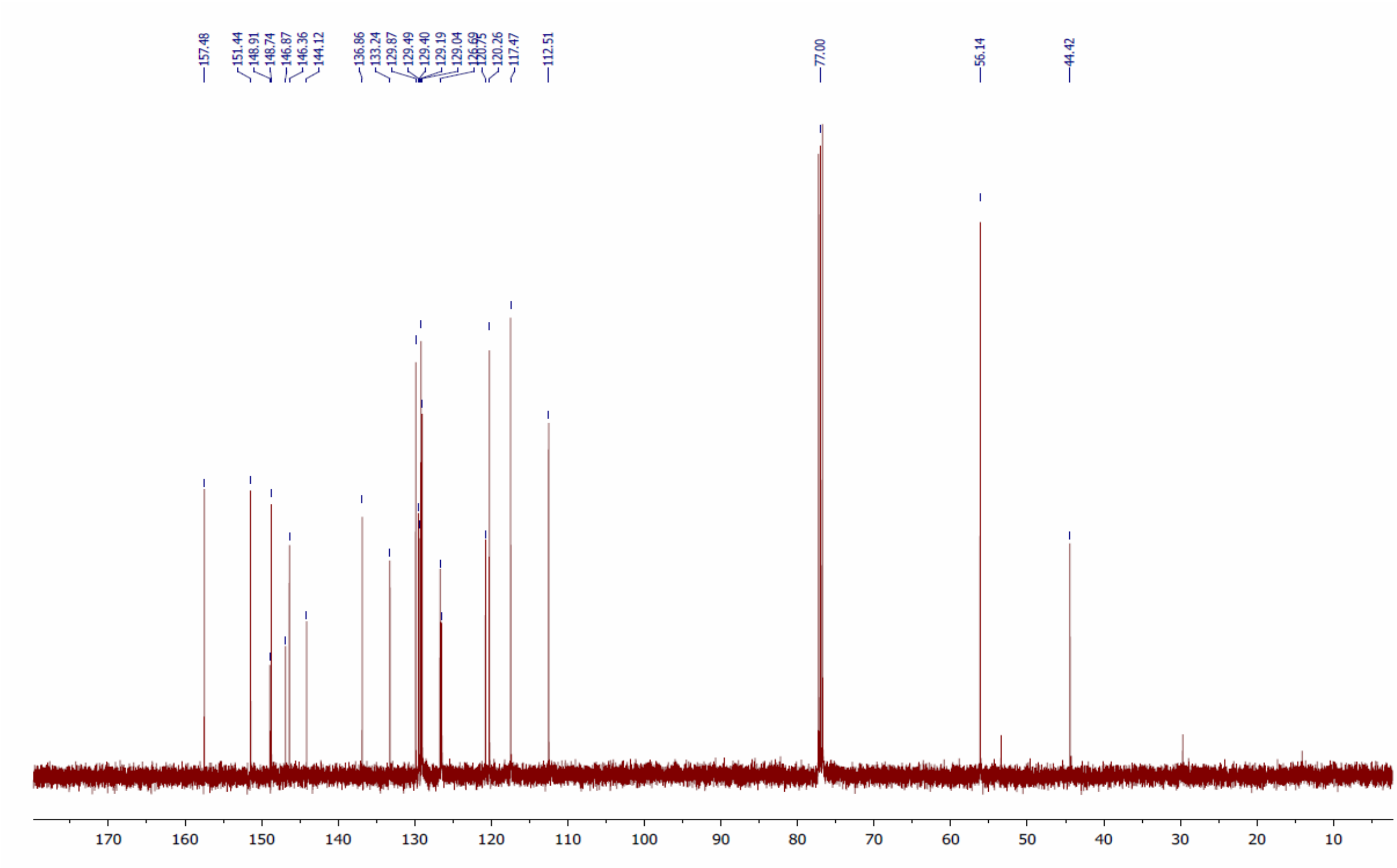

^{1}H NMR of compound (5d):

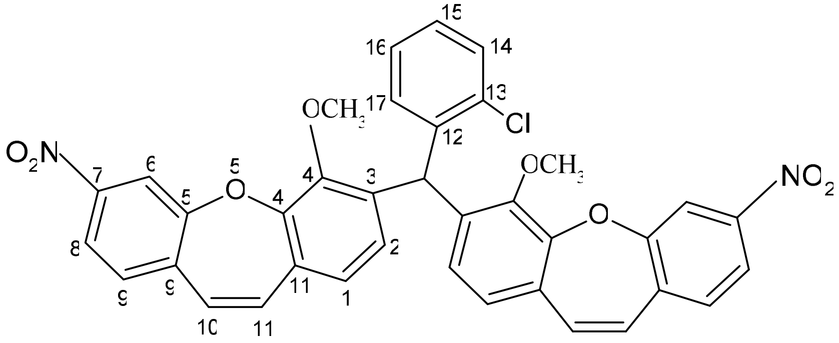

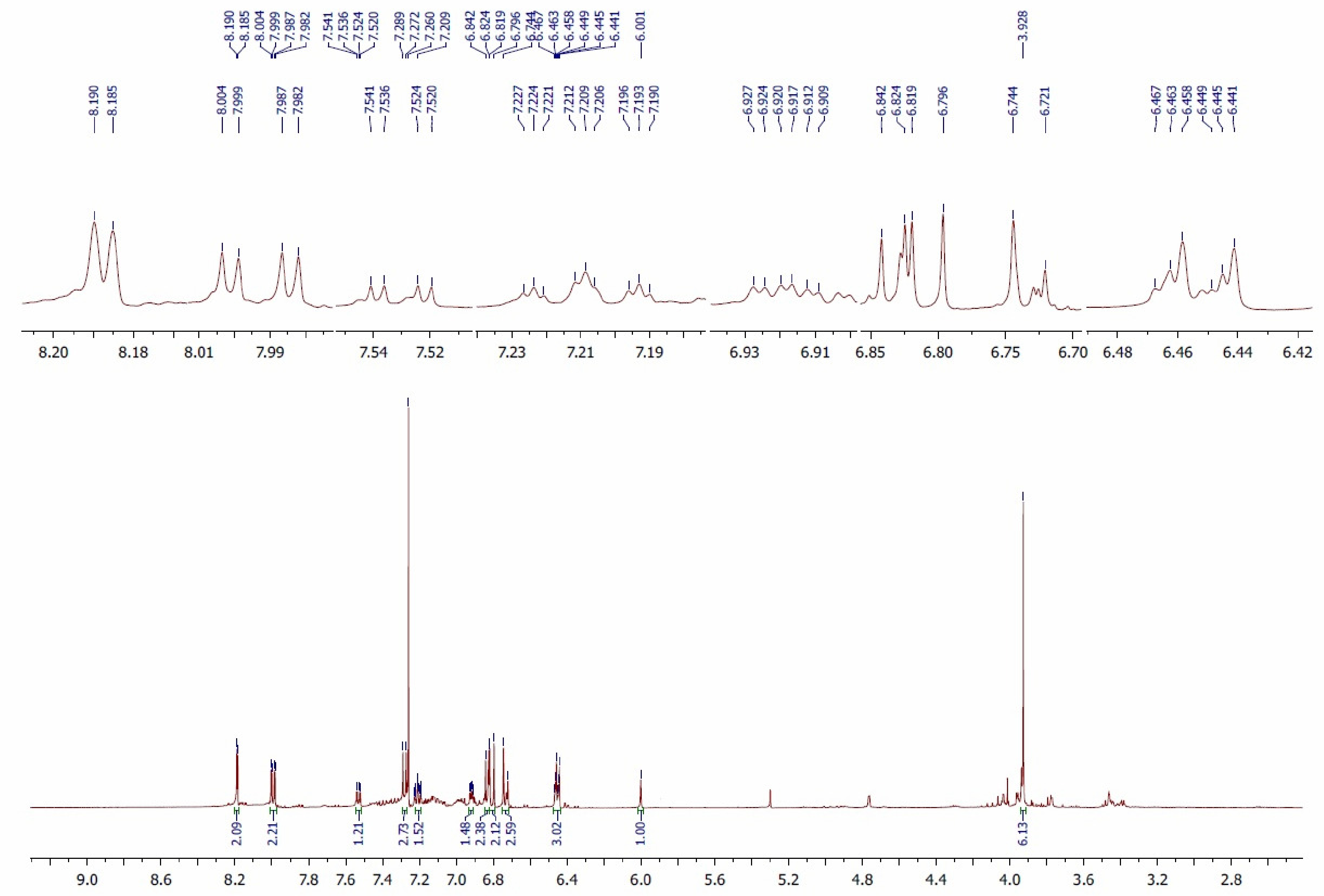

^{13}C NMR of compound (5d):

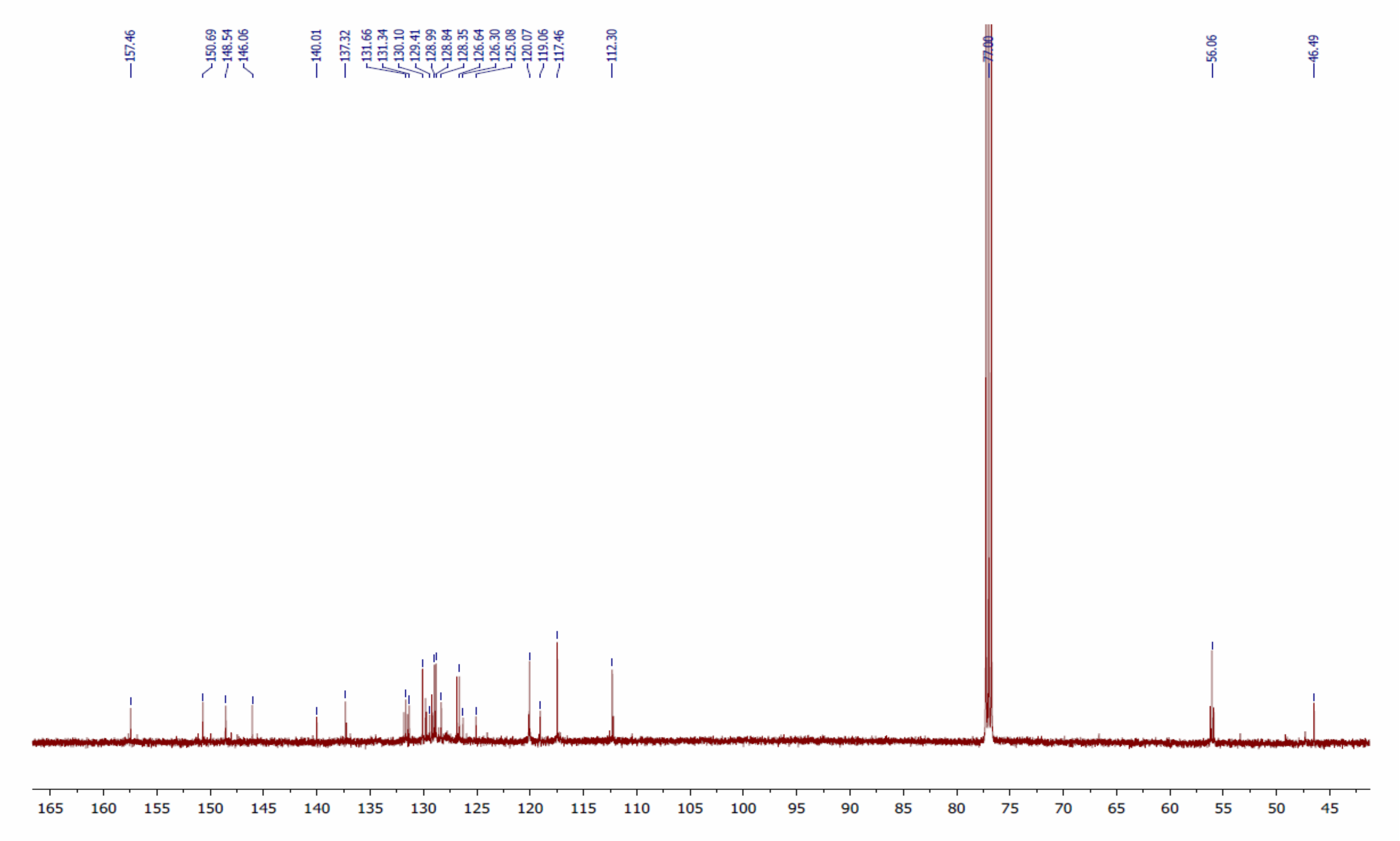

^{1}H NMR of compound (5e):

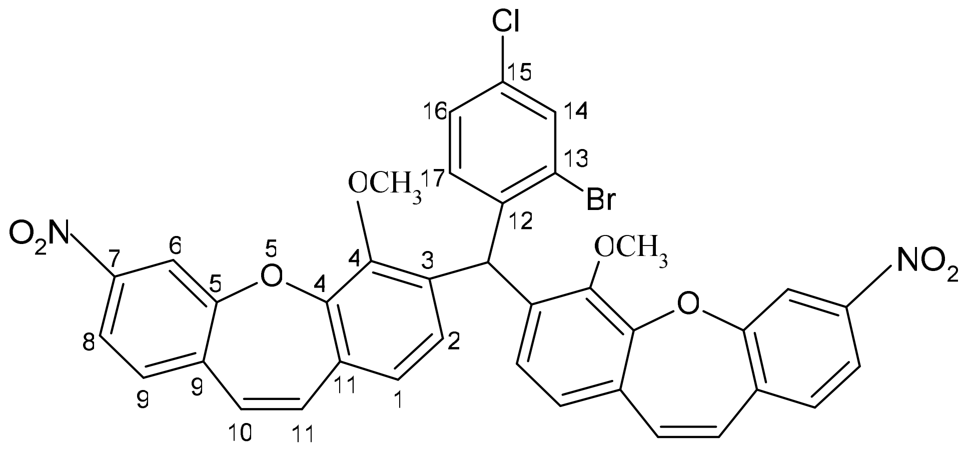

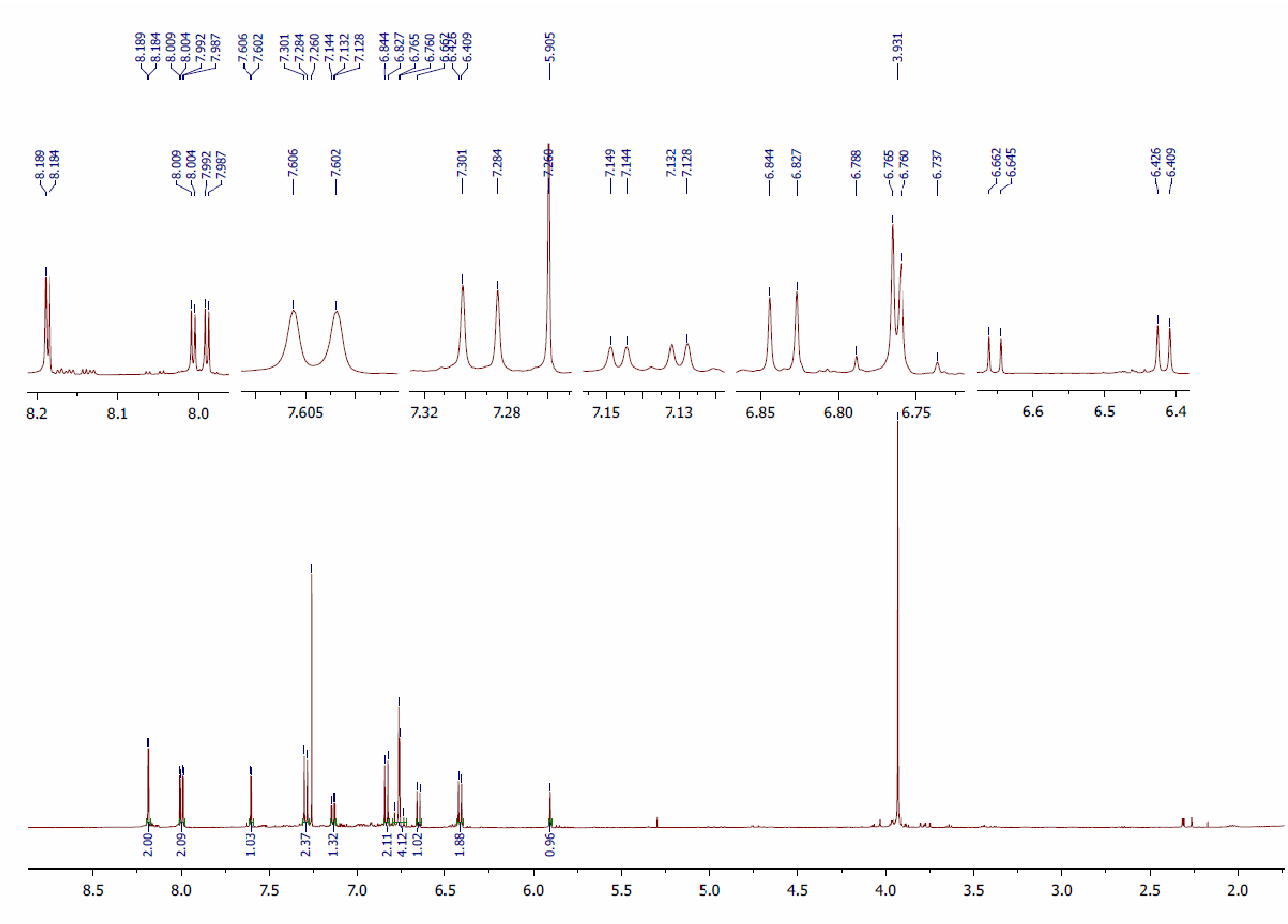
8.189 8.184 8.009 8.004 7.992 7.987 7.606 7.602 7.301 7.284 7.260 7.144 7.132 7.128 6.844 6.827 6.765 6.760 6.662 6.426 6.409
5.905
3.931
2.00 2.09 1.03 2.37 1.32 2.11 4.12 1.02 1.88 0.96

^{13}C NMR of compound (5e):

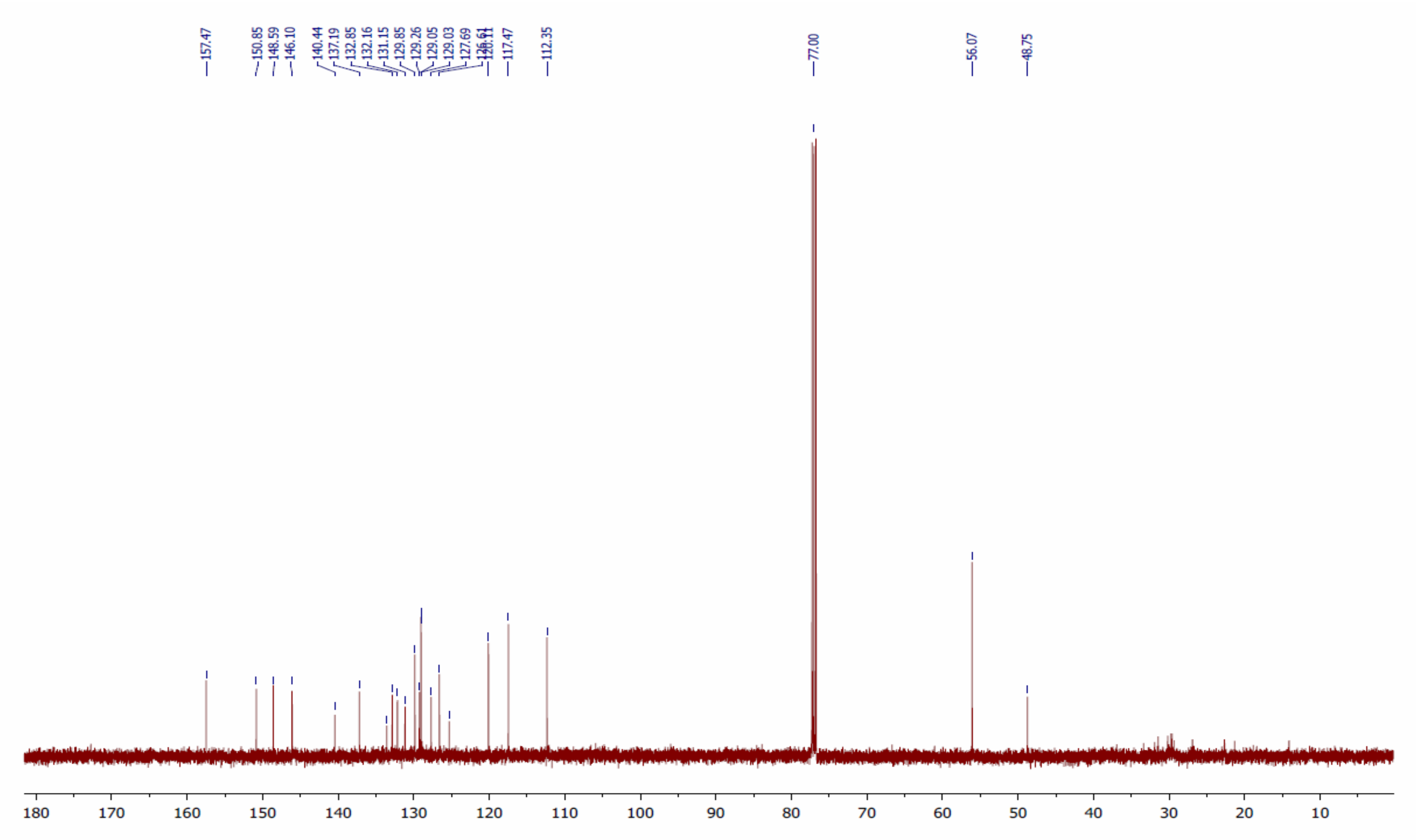

^{1}H NMR of compound (5f):

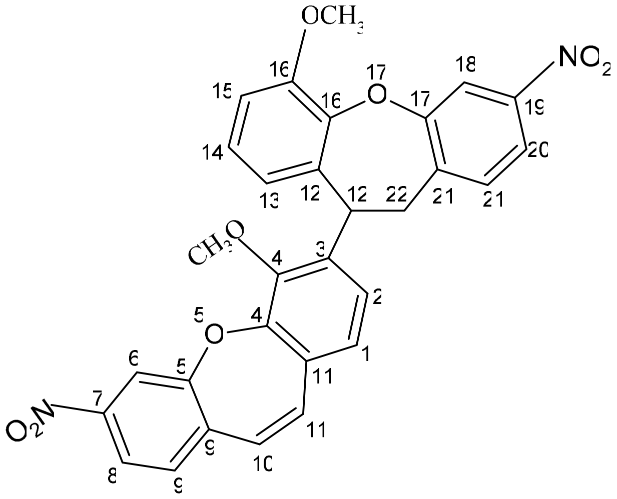

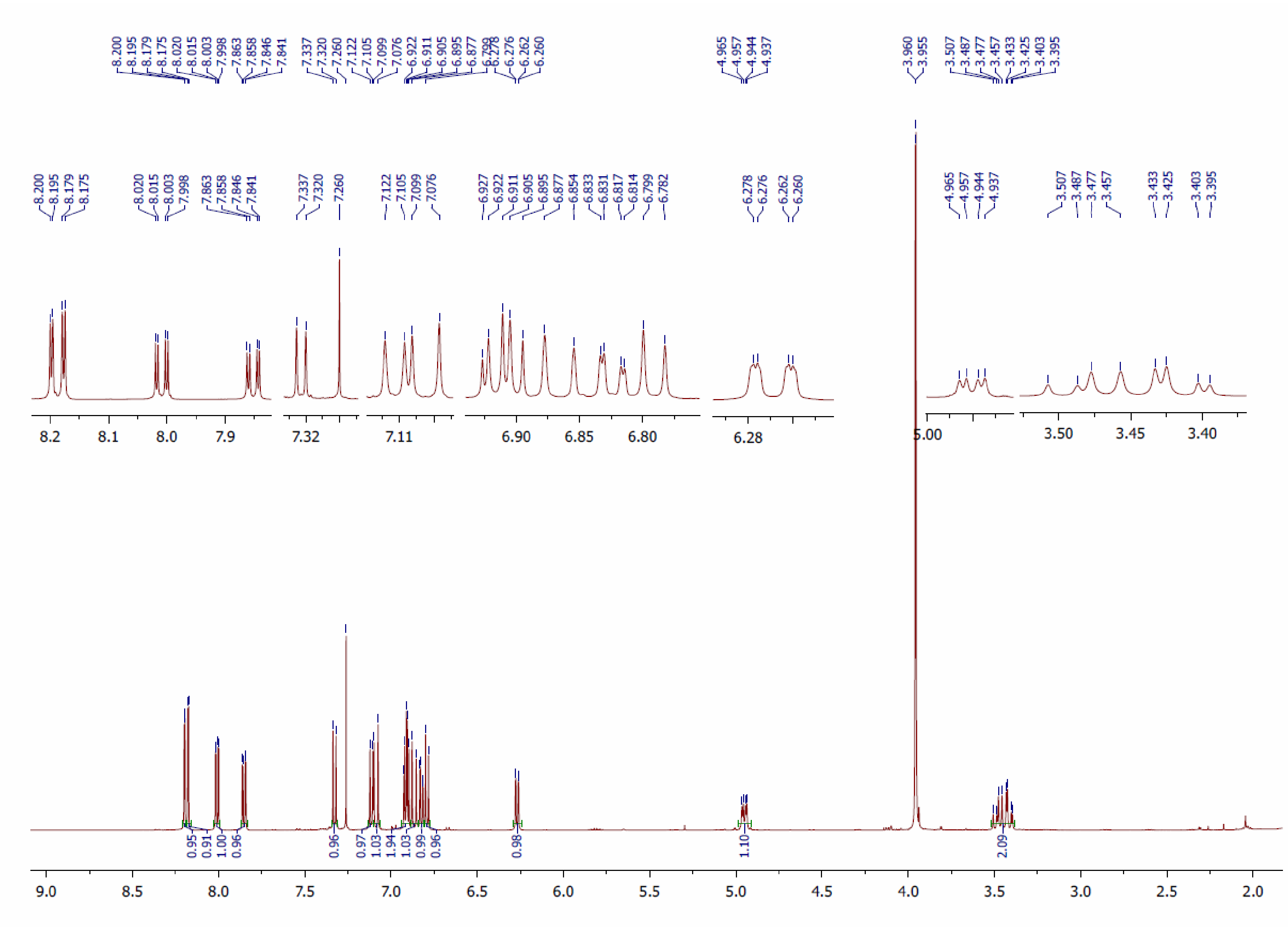
8.200 8.195 8.179 8.175 8.020 8.015 8.003 7.998 7.863 7.858 7.846 7.841 7.337 7.320 7.260 7.122 7.105 7.099 7.076 6.922 6.911 6.905 6.895 6.877 6.799 6.278 6.276 6.262 6.260
4.965 4.957 4.944 4.937
3.960 3.955
3.507 3.487 3.477 3.457 3.433 3.425 3.403 3.395
0.95 0.91 1.00 0.96 0.96 0.97 1.03 1.94 1.03 0.99 0.96 0.98 1.10 2.09
9.0 8.5 8.0 7.5 7.0 6.5 6.0 5.5 5.0 4.5 4.0 3.5 3.0 2.5 2.0

^{13}C NMR of compound (5f):

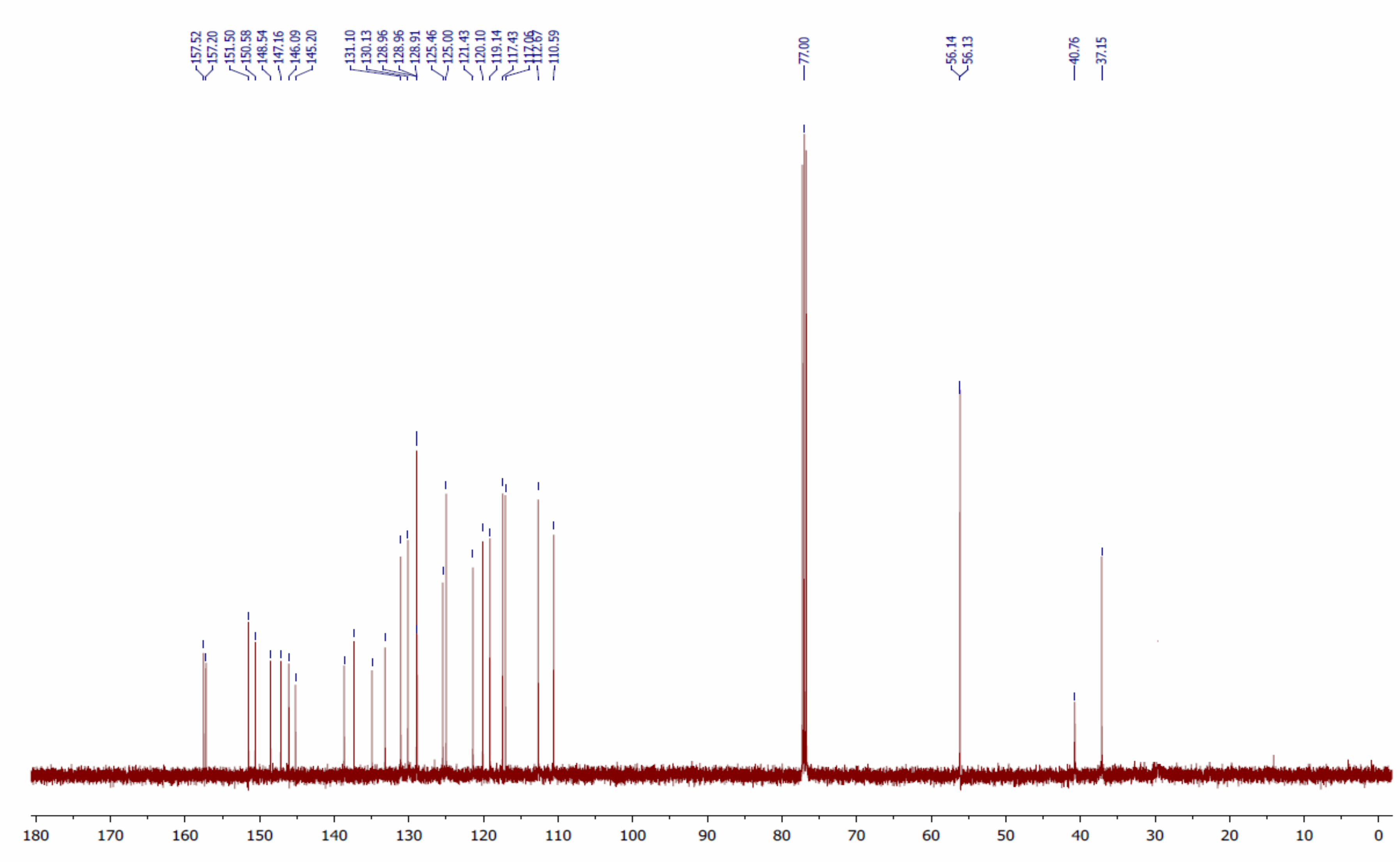

Copies of ^{1}H NMR and ^{13}C NMR spectra of (6a-b)

^{1}H NMR of compound (6a):

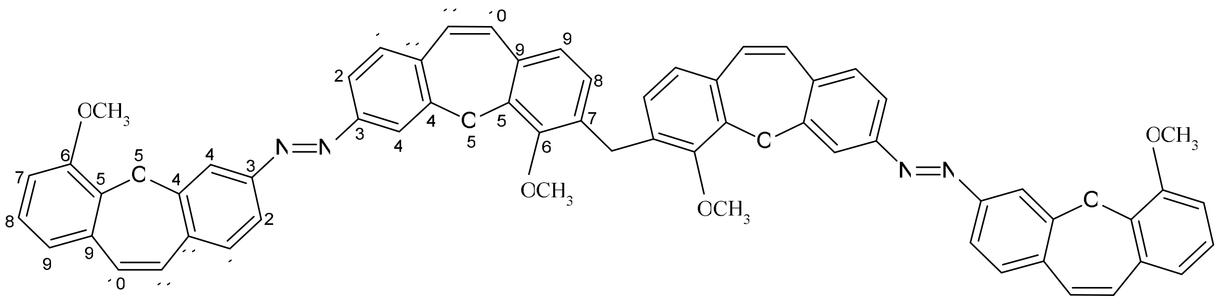

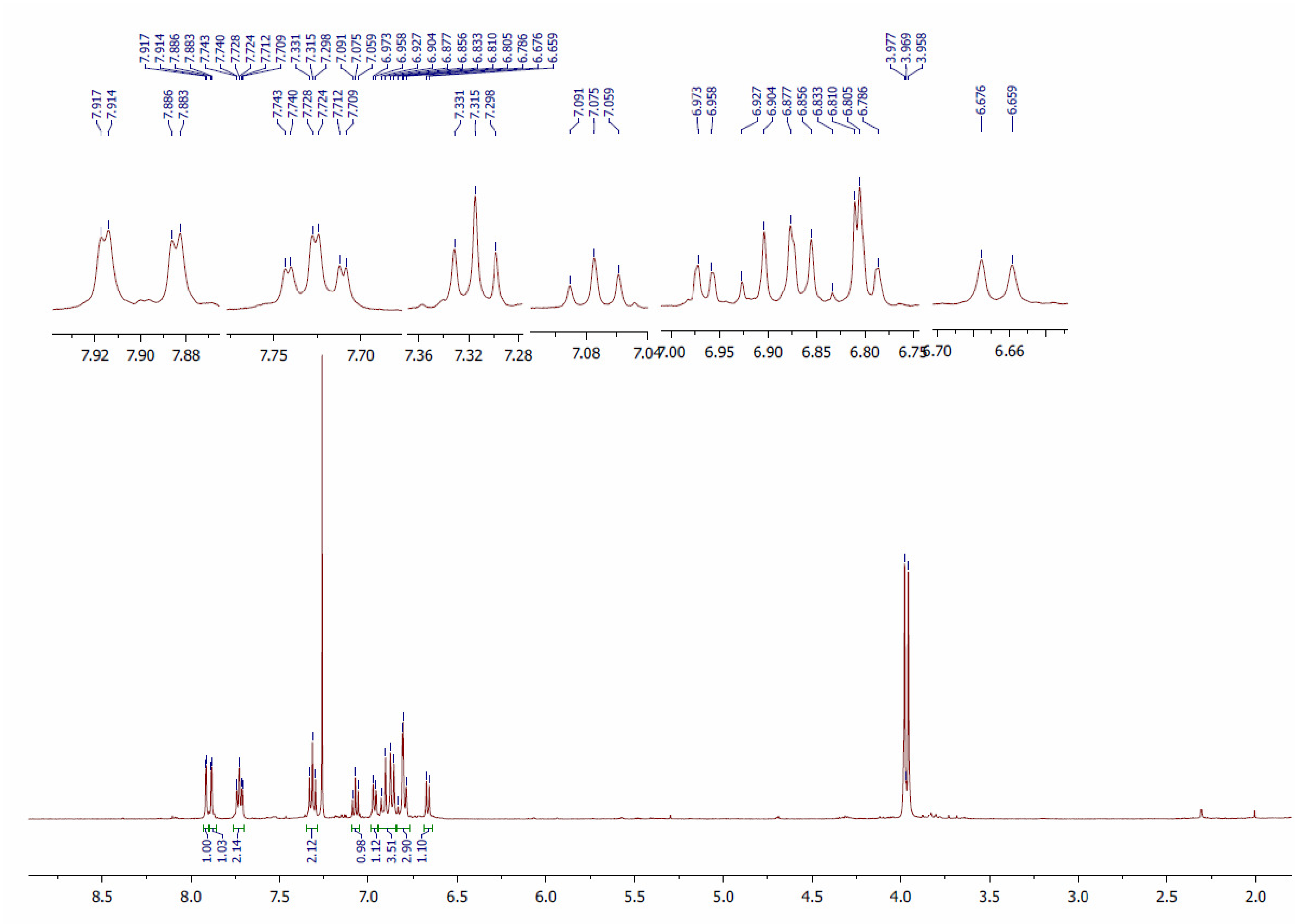
7.917
7.914
7.886
7.883
7.743
7.740
7.728
7.724
7.712
7.709
7.331
7.315
7.298
7.091
7.075
7.059
6.973
6.958
6.927
6.904
6.877
6.856
6.833
6.810
6.805
6.786
6.676
6.659
3.977
3.969
3.958
1.00
1.03
2.14
2.12
0.98
1.12
3.51
2.90
1.10
8.5
8.0
7.5
7.0
6.5
6.0
5.5
5.0
4.5
4.0
3.5
3.0
2.5
2.0

^{13}C NMR of compound (6a):

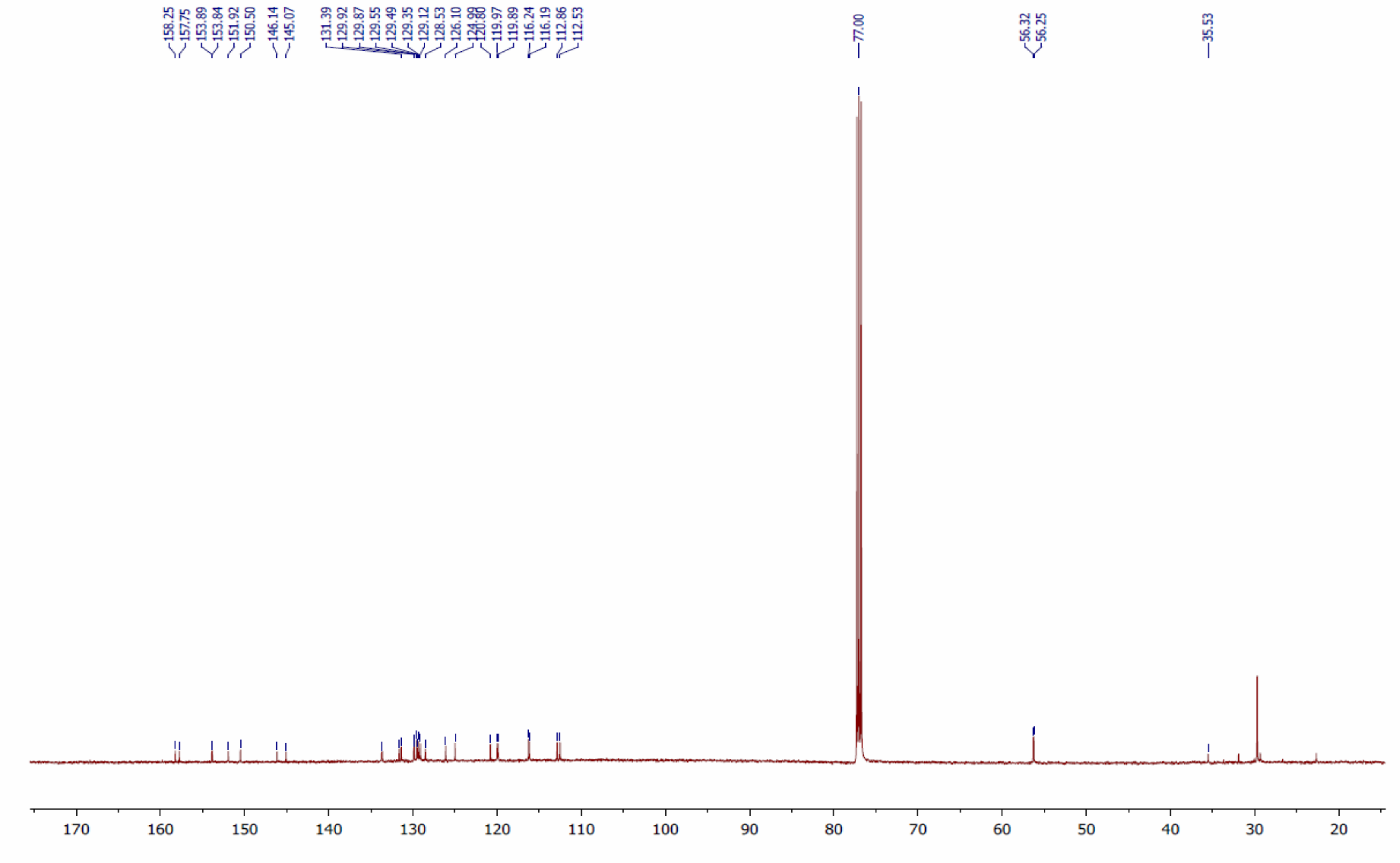

^{1}H NMR of compound (6b):

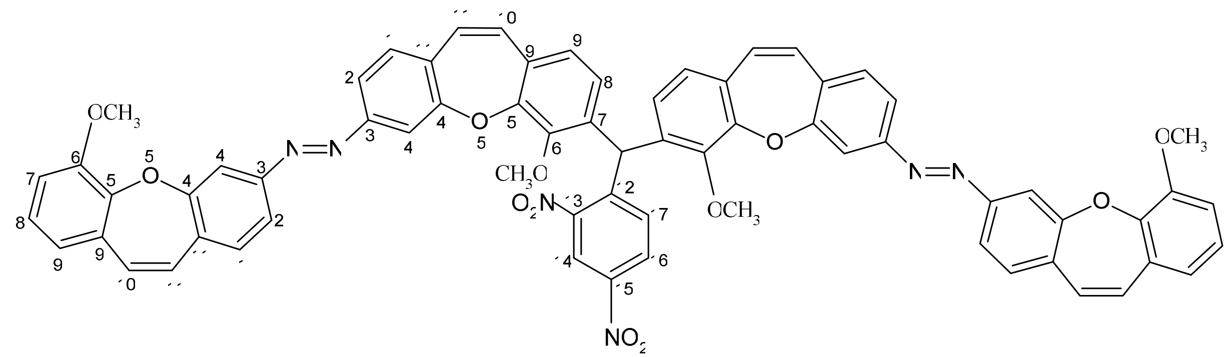

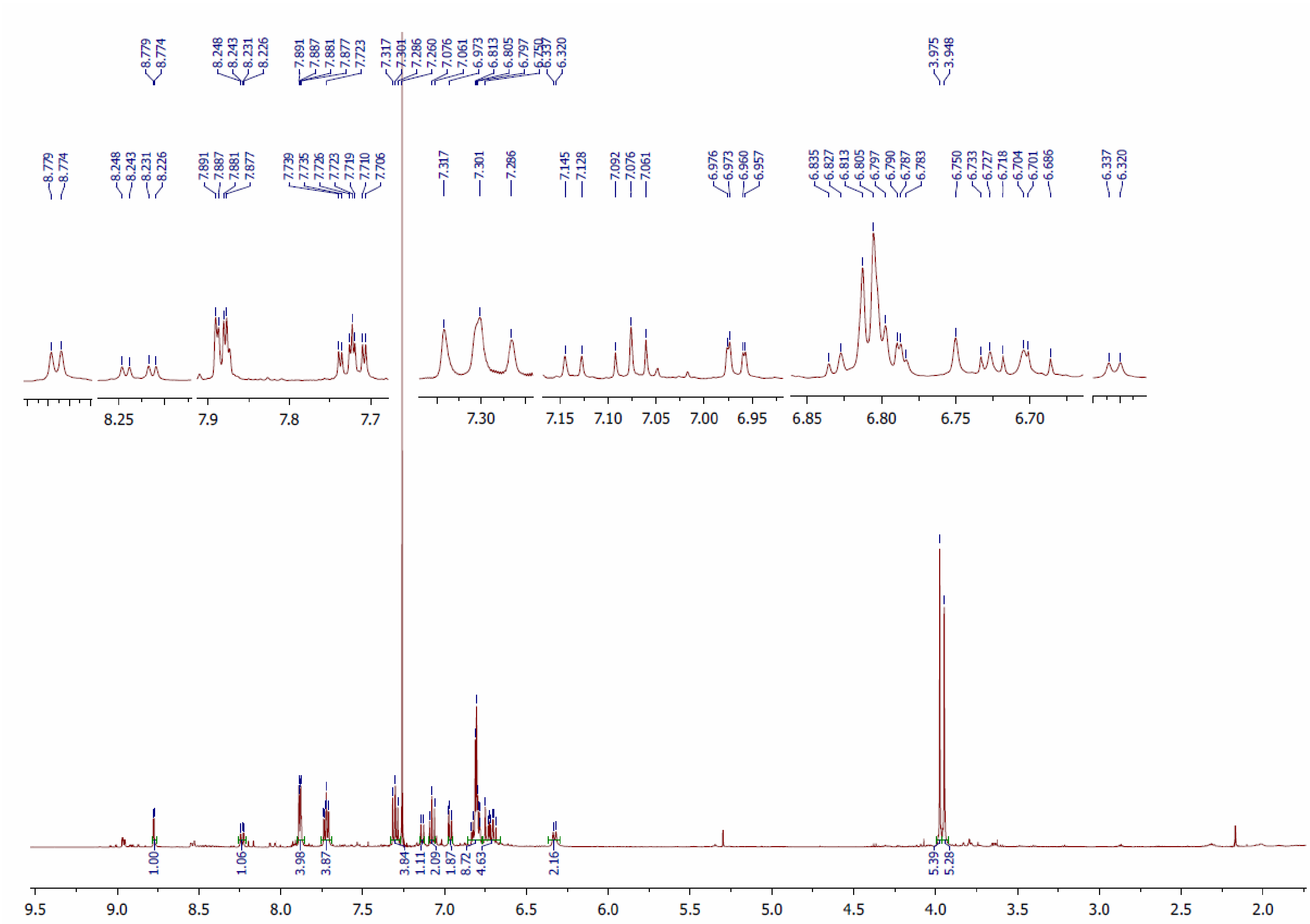
8.779 8.774 8.248 8.243 8.231 8.226 7.891 7.887 7.881 7.877 7.723 7.317 7.301 7.286 7.260 7.076 7.061 6.973 6.813 6.805 6.797 6.750 6.337 6.320 3.975 3.948
8.779 8.774 8.248 8.243 8.231 8.226 7.891 7.887 7.881 7.877 7.739 7.735 7.726 7.723 7.719 7.710 7.706 7.317 7.301 7.286 7.145 7.128 7.092 7.076 7.061 6.976 6.973 6.960 6.957 6.835 6.827 6.813 6.805 6.797 6.790 6.787 6.783 6.750 6.733 6.727 6.718 6.704 6.701 6.686 6.337 6.320
1.00 1.06 3.98 3.87 3.84 1.11 2.09 1.87 8.72 4.63 2.16 5.39 5.28
9.5 9.0 8.5 8.0 7.5 7.0 6.5 6.0 5.5 5.0 4.5 4.0 3.5 3.0 2.5 2.0

^{13}C NMR of compound (6b):

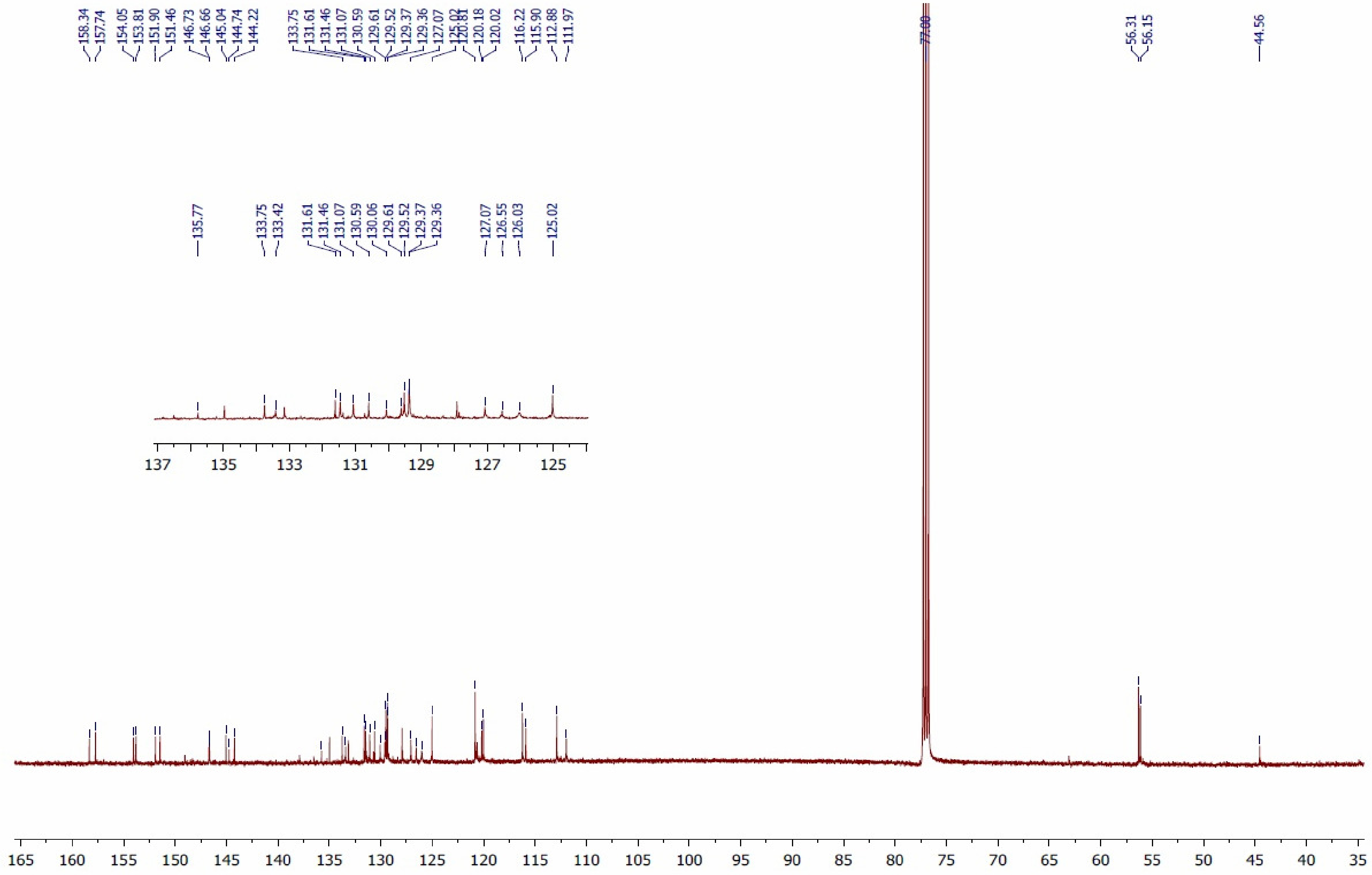

^{1}H NMR of compound (7):

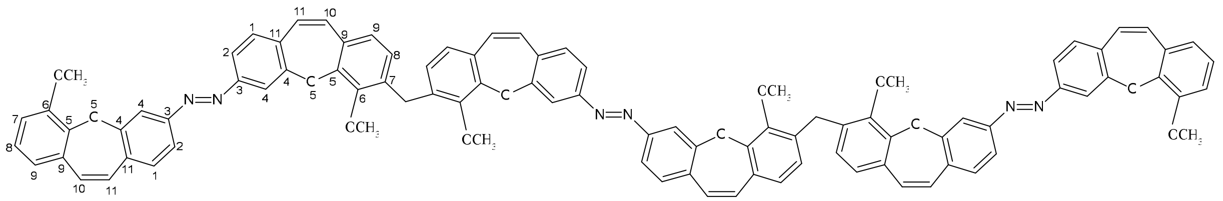

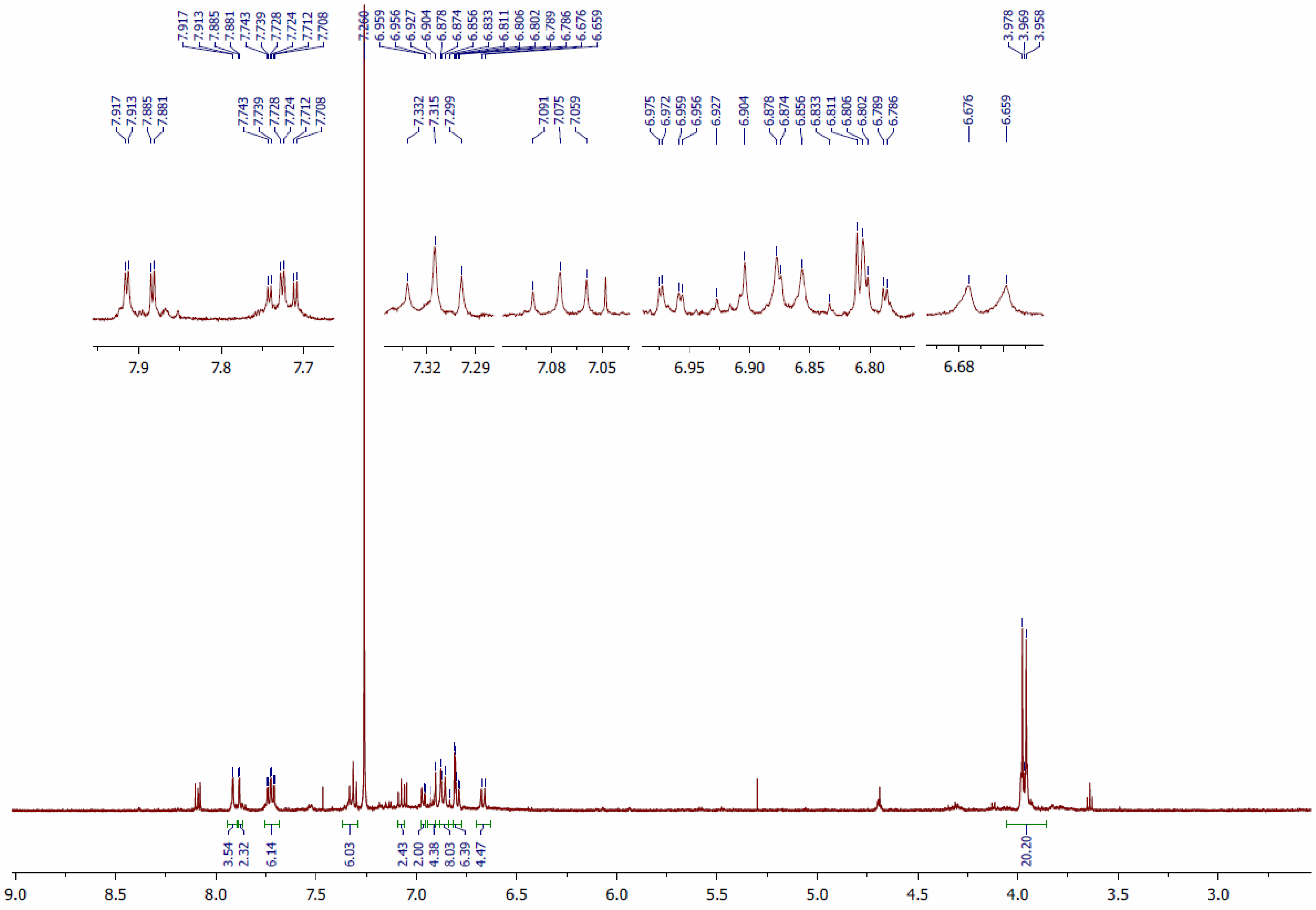

7.917
7.913
7.885
7.881
7.743
7.739
7.728
7.724
7.712
7.708
7.260
6.959
6.956
6.927
6.904
6.878
6.874
6.856
6.833
6.811
6.806
6.802
6.789
6.786
6.676
6.659
3.978
3.969
3.958
7.332
7.315
7.299
7.091
7.075
7.059
6.975
6.972
6.959
6.956
6.927
6.904
6.878
6.874
6.856
6.833
6.811
6.806
6.802
6.789
6.786
6.676
6.659
3.54
2.32
6.14
6.03
2.43
2.00
4.38
8.03
6.39
4.47
20.20

^{1}H NMR of compound (8):

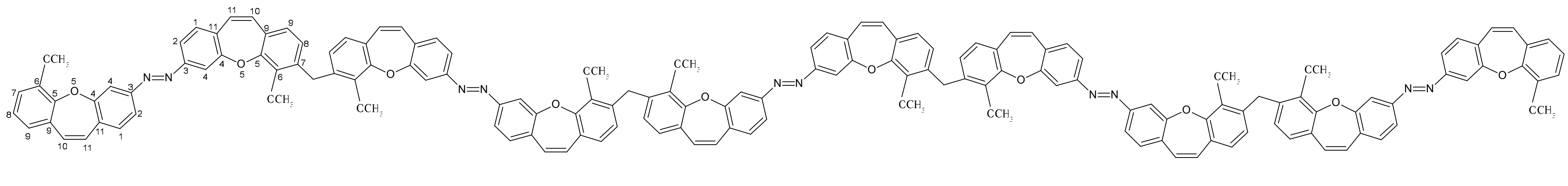

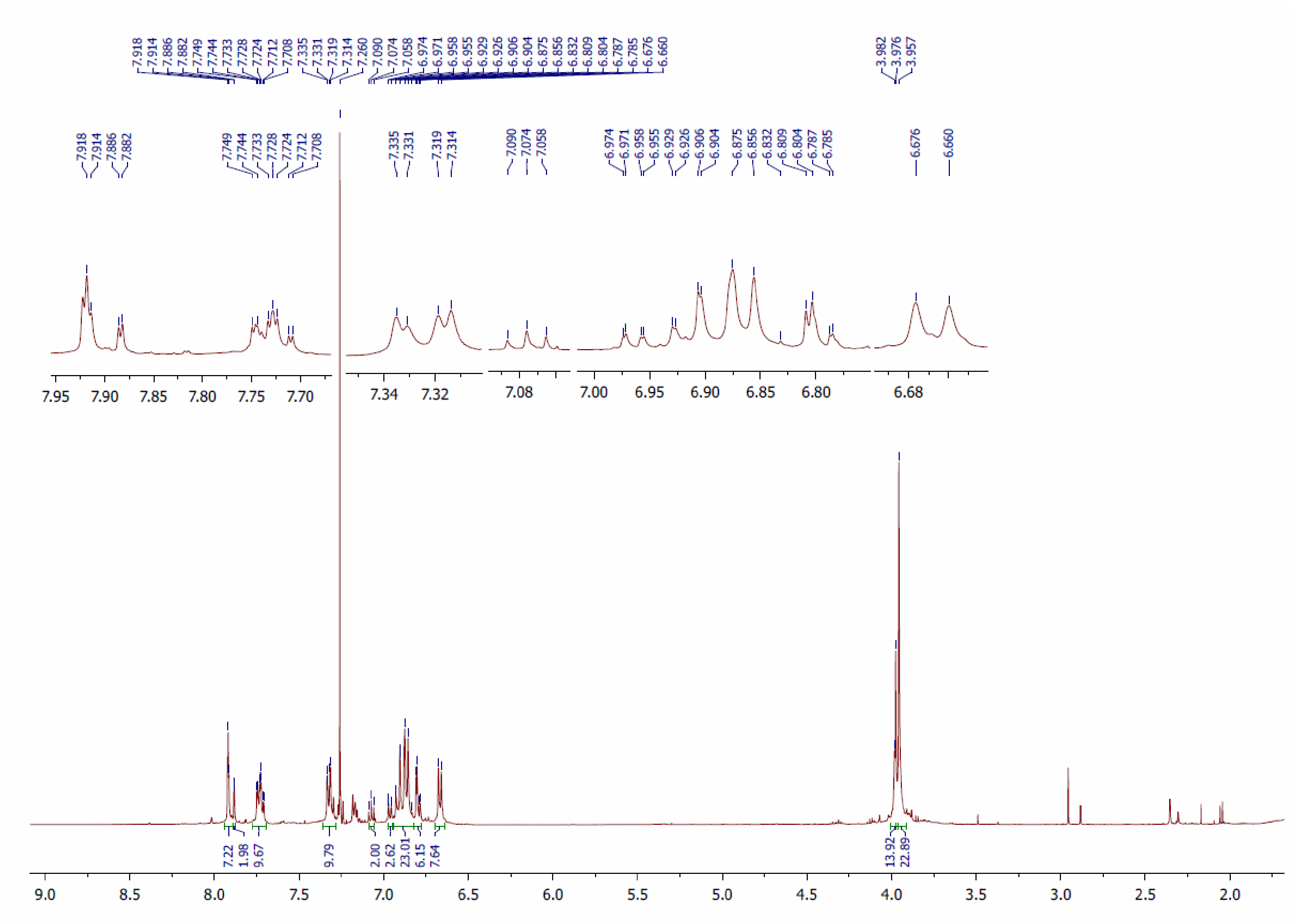

^{13}C NMR of compound (8):

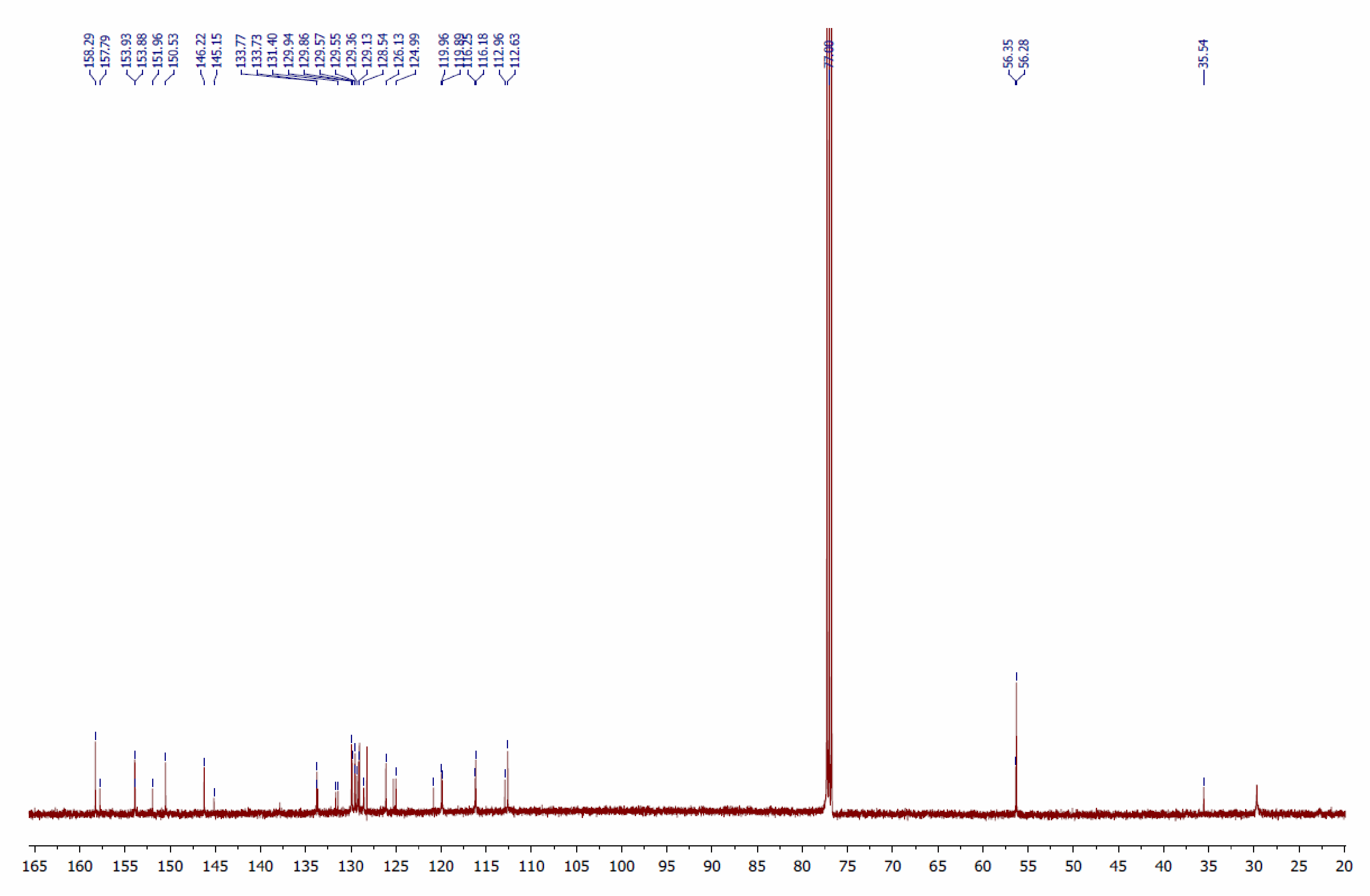

Table S2. The maximum for transitions π–π* of compounds (**2a,2b,2e-2g**).

compound		λ max [nm]
**2a**	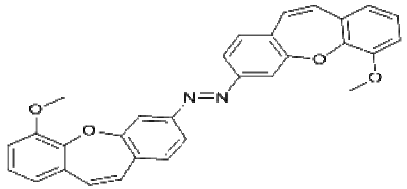	409
**2b**	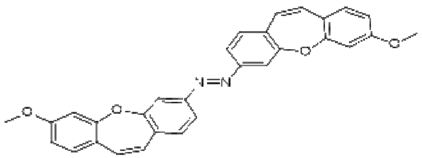	434
**2e**	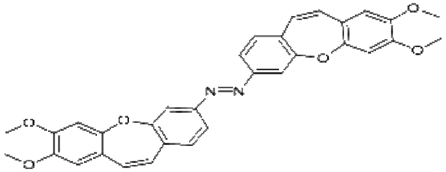	314, 446
**2f**	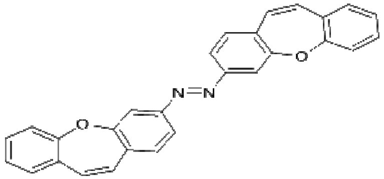	371
**2g**	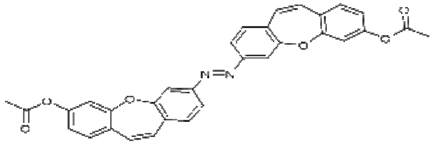	400

References

----

[1] M.J. Frisch, G.W. Trucks, H.B. Schlegel, G.E. Scuseria, M.A. Robb, J.R. Cheeseman, J.A. Montgomery, T. Vreven, K.N. Kudin, J.C. Burant, J.M. Millam, S.S. Iyengar, J.Tomasi, V. Barone, B. Mennucci, M. Cossi, G. Scalmani, N. Rega, G.A. Petersson, H. Nakatsuji, M. Hada, M. Ehara, K. Toyota, R. Fukuda, J. Hasegawa, M. Ishida, T.Nakajima, Y. Honda, O. Kitao, H. Nakai, M. Klene, X. Li, J.E. Knox, H.P. Hratchian, J.B. Cross, V. Bakken, C. Adamo, J. Jaramillo, R. Gomperts, R.E. Stratmann, O. Yazyev, A.J. Austin, R. Cammi, C. Pomelli, J.W. Ochterski, P.Y. Ayala, K. Morokuma, G.A. Voth, P. Salvador, J.J. Dannenberg, V.G. Zakrzewski, S. Dapprich, A.D. Daniels, M.C. Strain, O. Farkas, D.K. Malick, A.D. Rabuck, K.Raghavachari, J.B. Foresman, J.V. Ortiz, Q. Cui, A.G. Baboul, S. Clifford, J. Cioslowski, B.B. Stefanov, G. Liu, A. Liashenko, P. Piskorz, I. Komaromi, R.L. Martin, D.J. Fox, T. Keith, M.A. Al-Laham, C.Y. Peng, A. Nanayakkara, M. Challacombe, P.M.W. Gill, B. Johnson, W. Chen, M.W. Wong, C. Gonzalez, J.A. Pople, Gaussian Inc., Wallingford, CT, **2004** (Gaussian 03, Revision E.01).

[2] J. Tomasi, B. Mennucci, R. Cammi, Quantum mechanical continuum solvation models, *Chem. Rev*. **2005**, *105*, 2999.
